# Effects of the Wastewater Flow Rate on Interactions between the Genus *Nitrosomonas* and Diverse Populations in an Activated Sludge Microbiome

**DOI:** 10.1264/jsme2.ME18108

**Published:** 2018-12-22

**Authors:** Takashi Narihiro, Masaru Konishi Nobu, Tomoyuki Hori, Tomo Aoyagi, Yuya Sato, Tomohiro Inaba, Hidenobu Aizawa, Hideyuki Tamaki, Hiroshi Habe

**Affiliations:** 1 Bioproduction Research Institute, National Institute of Advanced Industrial Science and Technology (AIST) Central 6, 1–1–1, Higashi, Tsukuba, Ibaraki 305–8566 Japan; 2 Environmental Management Research Institute, National Institute of Advanced Industrial Science and Technology (AIST) 16–1, Onogawa, Tsukuba, Ibaraki 305–8569 Japan

**Keywords:** activated sludge, microbial community, network analysis, *Nitrosomonas*, *Thiothrix*

## Abstract

The present study characterized the interactions of microbial populations in activated sludge systems during the operational period after an increase in the wastewater flow rate and consequential ammonia accumulation using a 16S rRNA gene sequencing-based network analysis. Two hundred microbial populations accounting for 81.8% of the total microbiome were identified. Based on a co-occurrence analysis, *Nitrosomonas*-type ammonia oxidizers had one of the largest number of interactions with diverse bacteria, including a bulking-associated *Thiothrix* organism. These results suggest that an increased flow rate has an impact on constituents by changing ammonia concentrations and also that *Nitrosomonas*- and *Thiothrix*-centric responses are critical for ammonia removal and microbial community recovery.

Activated sludge systems, a representative biological treatment technology, are widely used to remediate municipal and industrial wastewaters. Activated sludge consists of a number of microorganisms with metabolic activities to remove carbon and nitrogen in wastewaters (15, 27, 28, 32). By the early 2000s, the predominant and functionally important microorganisms in these systems were identified using 16S rRNA-targeted molecular approaches, such as clone libraries and fluorescence *in situ* hybridization (42, 44). In the past several years, comprehensive surveys on the microbiomes of full-scale systems have been conducted by employing high-throughput DNA sequencing technology (9, 11, 13, 18, 20, 23, 25, 39, 46). These studies identified common microbial constituents in these systems and reported the effects of seasonal/regional variations on microbiome assemblages. However, limited information is currently available on the effects of changes in operating conditions (*e.g*., the organic loading rate, wastewater type, and aeration rate) on microbiomes in full-scale activated sludge systems. Without an understanding of sludge microbiome dynamics under disturbed conditions, the development of strategies to maintain treatment efficiency and stability may be difficult. In the present study, we investigated temporal changes in an activated sludge microbiome after an increase in the wastewater flow rate through 16S rRNA gene iTag sequencing with a special focus on the microorganisms associated with ammonia oxidation and filamentous sludge bulking.

Activated sludge samples were collected from two full-scale conventional activated sludge tanks in different flow lines treating municipal sewage wastewater in Japan (named AB1 and AB2 with a working volume of 9,000 m^3^ each). Samples were collected at six time points in duplicate after increasing the wastewater flow rate and stored at −20°C prior to DNA extraction. BOD, ammonia, nitrite, and nitrate in the sludge water were analyzed by standard methods for wastewater analyses (19). DNA was extracted from sludge samples by a direct lysis protocol that includes bead beating, phenol-chloroform extraction, and ethanol precipitation (30, 31, 37). 16S rRNA genes were amplified with the primer set Univ515F/Univ806R and Q5 DNA polymerase (New England Biolabs Japan, Tokyo, Japan) (1, 38). MiSeq sequencing was performed using v2 chemistry (Illumina, San Diego, CA, USA). Raw paired-end 16S rRNA gene reads were assembled and screened with mothur 1.35.1 using sequence length (≥200 nt) and quality score (≥30) cut-offs (40). Screened sequence data were grouped into OTUs with the UCLUST algorithm using a 97% sequence identity cut-off (8). Representative sequences for each OTU were aligned using PyNAST (4), chimeric sequences were removed using ChimeraSlayer (12), and phylogeny was assigned using blast retained on Greengenes database ver. 13_8 (29). Chao1 and coverage values were calculated by QIIME 1.9.1 (5). Weighted UniFrac distances were used for a principal coordinate analysis (PCoA) (26). Spearman’s rank correlation coefficients *rs* were calculated using PAST software (14). A co-occurrence network analysis was performed with significant interactions (*rs*>0.609 and *P* value<0.001), and the network was visualized by Cytoscape version 3.2.1 (43). A circular bar plot was illustrated using ggplot2 (45) and R studio ver. 1.1.45 (https://www.rstudio.com/) under R ver. 3.5.0 (17). To investigate the relationships between the relative abundances of OTUs and physicochemical parameters, a redundancy analysis (RDA) was performed using CANOCO software version 5 (Microcomputer Power, Ithaca, NY, USA) (24).

The operational performance of two activated sludge systems treating municipal sewage wastewater is shown in Fig. 1 and S1. AB1 and AB2 tanks achieved high BOD removal efficiency (>95%) during the period monitored. These tanks were continuously operated with a sewage wastewater flow rate of *ca*. 26,000 m^3^ d^−1^ before Nov. 14 and after Nov. 22. Between Nov. 15 and 21, the flow rate increased to more than 31,810 m^3^ d^−1^ (up to 37,150 and 37,320 m^3^ d^−1^ in the AB1 and AB2 tanks, respectively) due to periodic maintenance of the final sedimentation tank. Although the flow rate decreased to the levels of standard operational conditions after Nov. 22, the concentrations of nitrogen constituents increased and fluctuated, *i.e*., ammonia concentrations increased to 1.2–6.4 mg L^−1^ between Dec. 8 and Jan. 18 and decreased to <0.9 mg L^−1^ on Jan. 25, nitrite concentrations were 2.1–7.0 mg L^−1^ in late November and remained at low levels of <2.1 mg L^−1^ by Jan. 25, and nitrate accumulated to concentrations of 11–20 mg L^−1^ between Dec. 7 and Jan. 5 and then returned to the level of standard operational conditions on Jan. 25 (<8.1 mg L^−1^; Fig. 1B).

To elucidate the effects of changes in the wastewater flow rate on the composition of the activated sludge microbiome, 16S rRNA gene iTag libraries were constructed for sludge samples collected from the AB1 and AB2 tanks between Dec. 7 and Jan. 25 (Tables S1 and S2). A total of 2,030,063 reads of 16S rRNA genes were obtained and assigned to 37,151 OTUs. High Good’s coverage values (>96%) suggested that the OTUs obtained adequately estimated the diversity of the activated sludge microbiome. Bacterial taxa commonly associated with activated sludge (11, 13, 20, 25, 46), *Alphaproteobacteria*, *Betaproteobacteria*, *Gammaproteobacteria*, *Deltaproteobacteria*, and *Bacteroidetes*, were detected in all samples and accounted for >72.2% of the total population (Fig. 1C). A comparison of microbiome compositions between samples using a weighted UniFrac-based 3D principal coordinate analysis clearly showed that compositions shifted with time (Fig. S2). These results suggest that particular microbial populations may have been affected by changes in the wastewater flow rate and resulting accumulation of nitrogen constituents in AB1 and AB2.

To analyze the interactions between major microbial constituents, we focused on the top 200 abundant OTUs accounting for 81.8% of the total microbiome dataset across 24 sludge samples from AB1 and AB2. We found two OTUs (AB23287 and AB38882) related to *Nitrosomonas*-type ammonia oxidizing bacteria (AOB) with a relatively low abundance by Jan. 18 (<0.1% of the total population) and marked increases on Jan. 25 up to the 71^st^–81^st^ percentile, respectively (0.5% of the total microbiome) (Fig. 2, S3A, and S3B, Table S2). The abundance-based network analysis indicated the complex co-occurrence patterns of dominant organisms, including a total of 2,509 (1,252 positive and 1,257 negative) significant interactions in activated sludge samples (Fig. S4A, Table S3). Two *Nitrosomonas*-related OTUs had a large number of degrees (edges) within the network (Fig. 3A and 3B). *Nitrosomonas*-related OTUs shared 26 positive and 27 negative interactions between OTUs belonging to the cultured phyla *Proteobacteria*, *Bacteroidetes*, *Spirochaetes*, *Verrucomicrobia*, *Planctomycetes*, and *Gemmatimonadetes* and uncultivated phyla TM7 (“*Ca*. Saccharibacteria”), OD1 (“*Ca*. Parcubacteria”), and SAR406 (“*Ca*. Marinimicrobia”). Previous studies revealed that nitrifying bacteria forge beneficial interactions with heterotrophic bacteria belonging to the phyla *Proteobacteria* (particularly the classes *Alphaproteobacteria* and *Gammaproteobacteria*), *Bacteroidetes*, and *Chloroflexi* through metabolite exchange (22, 35). Another proteomicbased study demonstrated that the addition of an enrichment culture of heterotrophic bacteria to a pure culture of *Nitrosomonas* induced the up-regulation of the ammonia oxidation pathway (41). Metabolites, such as siderophore (21), acyl-homoserine lactone (3), and amino acids (6, 7), have been shown to increase the growth of *Nitrosomonas* strains. For example, *Nitrosomonas*-type AOB may be stimulated *in situ* by siderophore production by positively interacting with *Myxococcales*-related organisms (OTUs AB38105 and AB35792; Fig. 2 and 3), a feature often observed for members of myxobacteria (10). Based on RDA, the proliferation of *Nitrosomonas*- and *Myxococcales*-type OTUs positively correlated with mixed liquor dissolved oxygen (MLDO) and the solid retention time (SRT), but negatively correlated with reactor ammonia concentrations (Fig. S5). These results suggest that high O_2_ and low ammonia are favorable for the growth of the above OTUs even though both compounds are substrates for *Nitrosomonas*. In addition, arrows of relatively abundant (>0.4% of the total activated sludge microbiome) OTUs having a negative correlation with two *Nitrosomonas*-related OTUs displayed reversed associations with the above parameters. In brief, metabolically diverse bacteria may play a supporting role for the recovery of *Nitrosomonas*-type AOB in the activated sludge system.

The relative abundance of an OTU AB4262 related to *Thiothrix*, which has been identified as a causative agent for filamentous sludge bulking (34), increased up to the 71^st^ percentile (0.32% abundance) of the total microbiome on Jan. 18, and then decreased to less than the 23^rd^ percentile (0.07% abundance) on Jan. 25 (Fig. 2 and S3C, Table S2). Although severe sludge bulking was not observed during the operation period in the AB1 and AB2 tanks (*i.e*., sludge volume indexes (SVI) showed stable values, 112–168; Fig. S1), a previous study reported that >0.19% of the *Thiothrix* population caused increases in suspended solids, which are likely associated with bulking events, and ammonia ion concentrations may have been one of the influential factors for *Thiothrix* proliferation in a full-scale nitrification and denitrification wastewater treatment plant in the U.S. (2). *Thiothrix*-related OTU AB4262 had a positive interaction with *Nitrosomonas*-type OTU AB38882 (Fig. S4B), even though its proliferation in the AB1 tank was earlier than that in the AB2 tank on Jan. 5 (Fig. 2). These results suggest that an increase in the wastewater flow rate and resulting ammonia accumulation trigger a co-increase in *Thiothrix* and *Nitrosomonas* populations. Although a *Thiothrix*-related population in an industrial wastewater treatment plant with bulking issues was shown to be suppressed by the addition of raw feed wastewater and the subsequent proliferation of a number of bacteria (36), interactions between *Thiothrix* and other bacterial constituents in activated sludge microbiomes remain unclear.

In conclusion, high-throughput microbiome profiling combined with a co-occurrence network analysis revealed complex interactions among diverse microorganisms in activated sludge tanks during the period after an increase in the wastewater flow rate. *Nitrosomonas*- and *Thiothrix*-related organisms may be affected by an increase in the flow rate. Although the reason for the proliferation of *Nitrosomonas* and *Thiothrix* organisms cannot currently be fully explained, further studies using metagenomics, metatranscriptomics, and metabolomics (16, 33) will provide more comprehensive information for elucidating the key factors shaping interactions between *Nitrosomonas*, *Thiothrix*, and other microorganisms in the activated sludge microbiome.

The sequence reported in the present study was deposited in the DDBJ database under DDBJ/EMBL/GenBank accession number DRA007026.

## Supplementary Information



## Figures and Tables

**Fig. 1:**
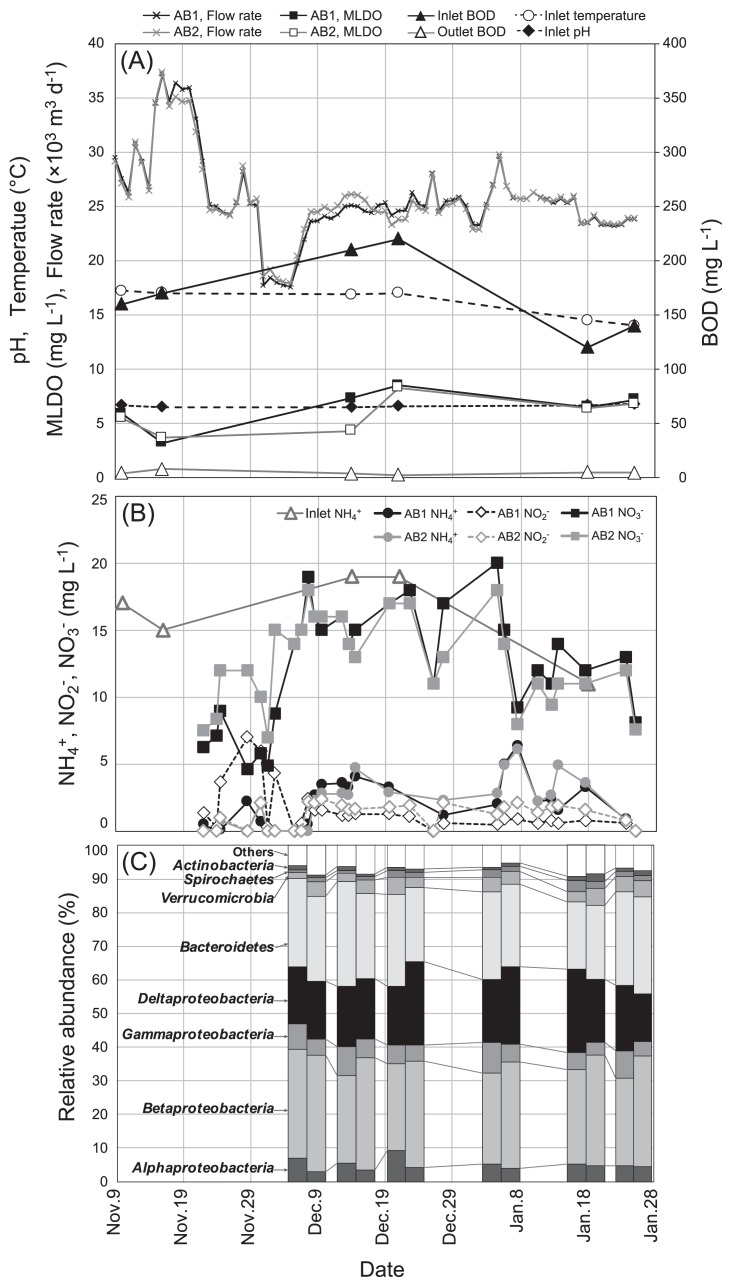
Changes in operational parameters of activated sludge tanks AB1 and AB2 treating municipal sewage. (A) Open circle, inlet temperature (°C); closed diamond, inlet pH; black line with a cross mark, wastewater flow rate (m^3^ d^−1^) in AB1; gray line with a cross mark, wastewater flow rate (m^3^ d^−1^) in AB2; closed triangle, inlet biological oxygen demand (BOD) (mg L^−1^); open triangle, outlet BOD (mg L^−1^); closed square, mixed liquor dissolved oxygen (MLDO) (mg L^−1^) in AB1; open square, MLDO (mg L^−1^) in AB2. (B) Open triangle, inlet ammonia concentration (mg L^−1^); black circle, ammonia concentration (mg L^−1^) in AB1; gray circle, ammonia concentration (mg L^−1^) in AB2; open diamond with a black line, nitrite concentration (mg L^−1^) in AB1; open diamond with a gray line, nitrite concentration (mg L^−1^) in AB2; Black square, nitrate concentration (mg L^−1^) in AB1; gray square, nitrate concentration (mg L^−1^) in AB2. Arrows indicate the periods for sludge sampling from tanks. (C) The bar chart shows microbial population shifts at the phylum/class level. The microbiome compositions of AB1 and AB2 are shown in the left and right bars, respectively, in each period.

**Fig. 2:**
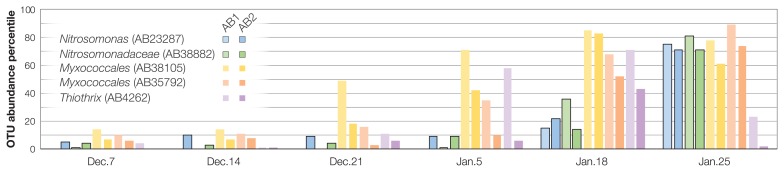
OTU abundance percentile for *Nitrosomonadaceae*-related OTUs AB23287 and AB38882 and positively interacting *Myxococcales* OTUs AB38105 and AB35792 and *Thiothrix* OTU AB4262 across the monitored period. The percentile is calculated as the percentage of the dominant 200 OTUs than which the subject OTU has a greater relative abundance.

**Fig. 3:**
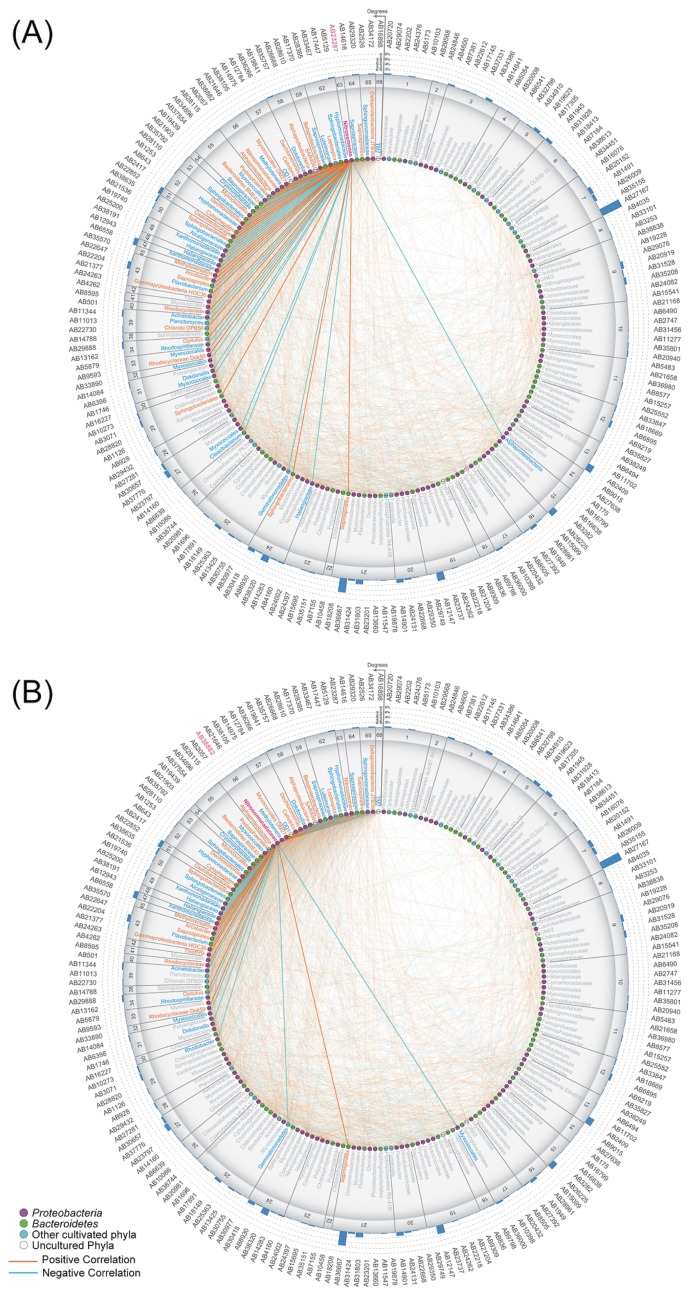
Degree-sorted co-occurrence networks of *Nitrosomonas* OTU AB23287 (A) and *Nitrosomonadaceae* OTU AB38882 (B) in activated sludge tanks AB1 and AB2. Nodes indicate dominant OTUs associated with *Proteobacteria* (magenta), *Bacteroidetes* (green), other cultivated phyla (light blue), and functionally unknown organisms (white), as shown in Table S3. Orange and blue color edges indicate positive and negative correlations, respectively. The circular bar plot shows relative abundances (%) in a total microbiome dataset comprising 24 sludge samples.
