# Supplementary material for: Effects of the Wastewater Flow Rate on Interactions between the Genus Nitrosomonas and Diverse Populations in an Activated Sludge Microbiome
Source: Microbes Environ. 2018 Dec 22;34(1):89–94. doi: 10.1264/jsme2.ME18108 (PMC6440735; doi:10.1264/jsme2.ME18108)
Supplement: Supplementary file 1 [file 34_89_s1.pdf]

Fig.S1

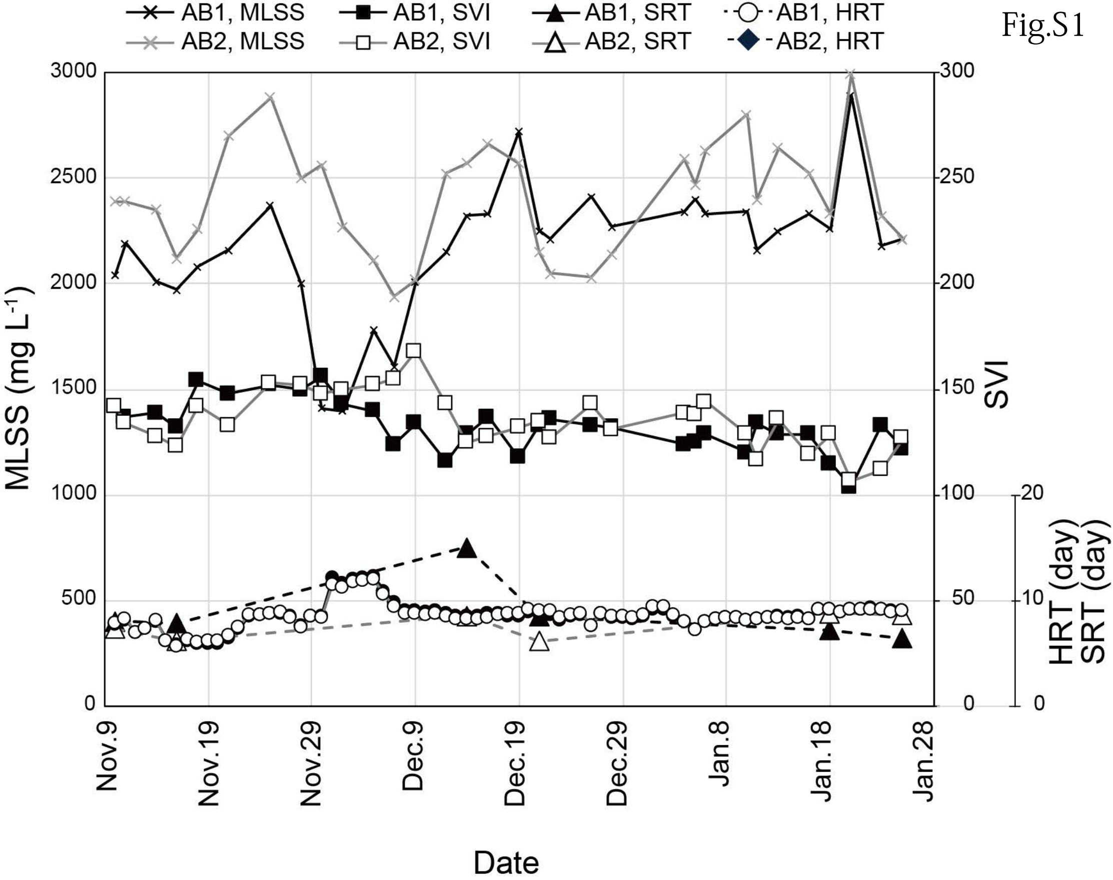
AB1, MLSS
AB1, SVI
AB1, SRT
AB1, HRT
AB2, MLSS
AB2, SVI
AB2, SRT
AB2, HRT
MLSS (mg L-1)
SVI
HRT (day)
SRT (day)
Date
3000
2500
2000
1500
1000
500
0
300
250
200
150
100
50
20
10
Nov.9
Nov.19
Nov.29
Dec.9
Dec.19
Dec.29
Jan.8
Jan.18
Jan.28

Fig.S2

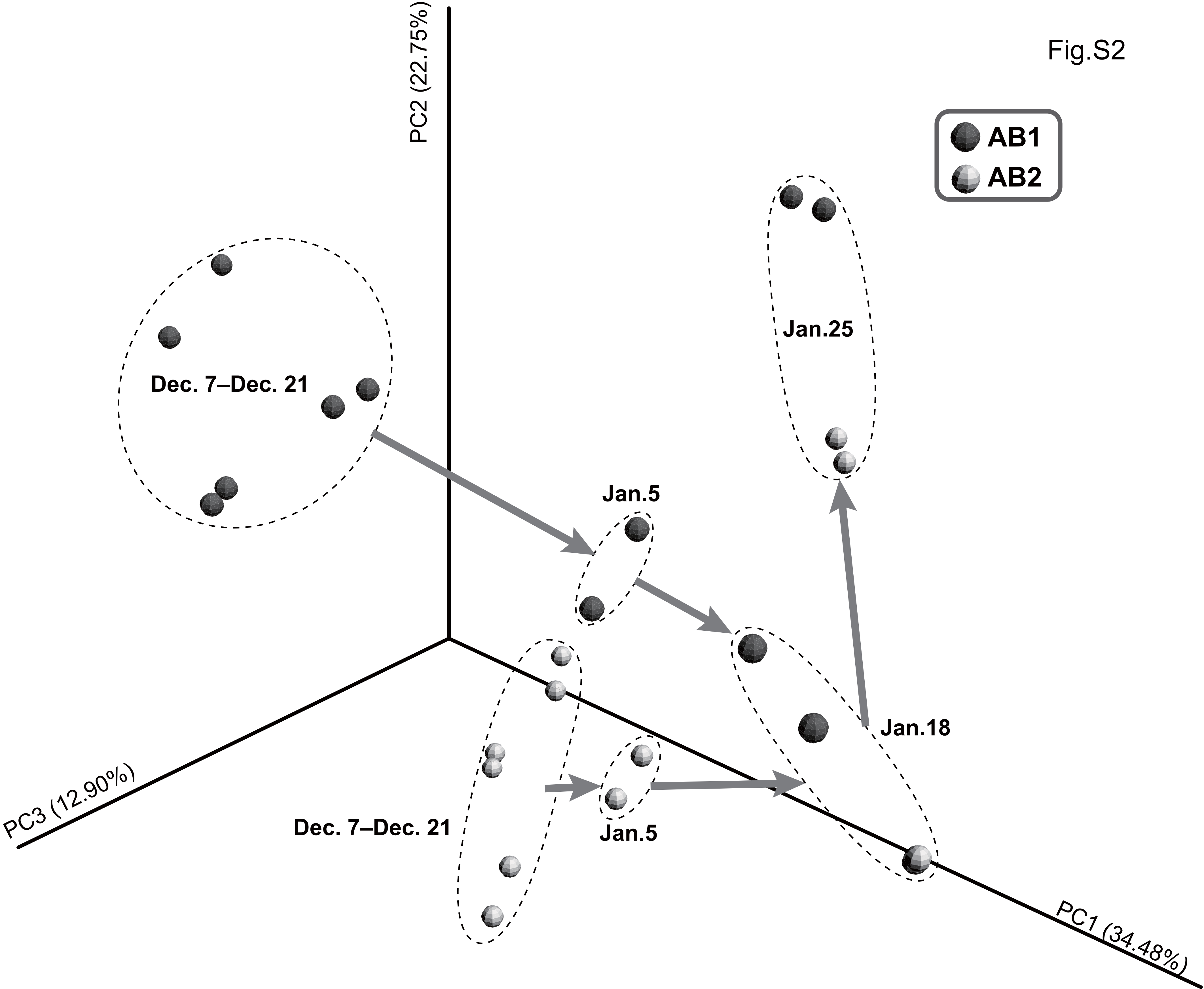

PC2 (22.75%)
PC3 (12.90%)
PC1 (34.48%)
AB1
AB2
Dec. 7–Dec. 21
Jan.5
Jan.18
Jan.25
Dec. 7–Dec. 21
Jan.5

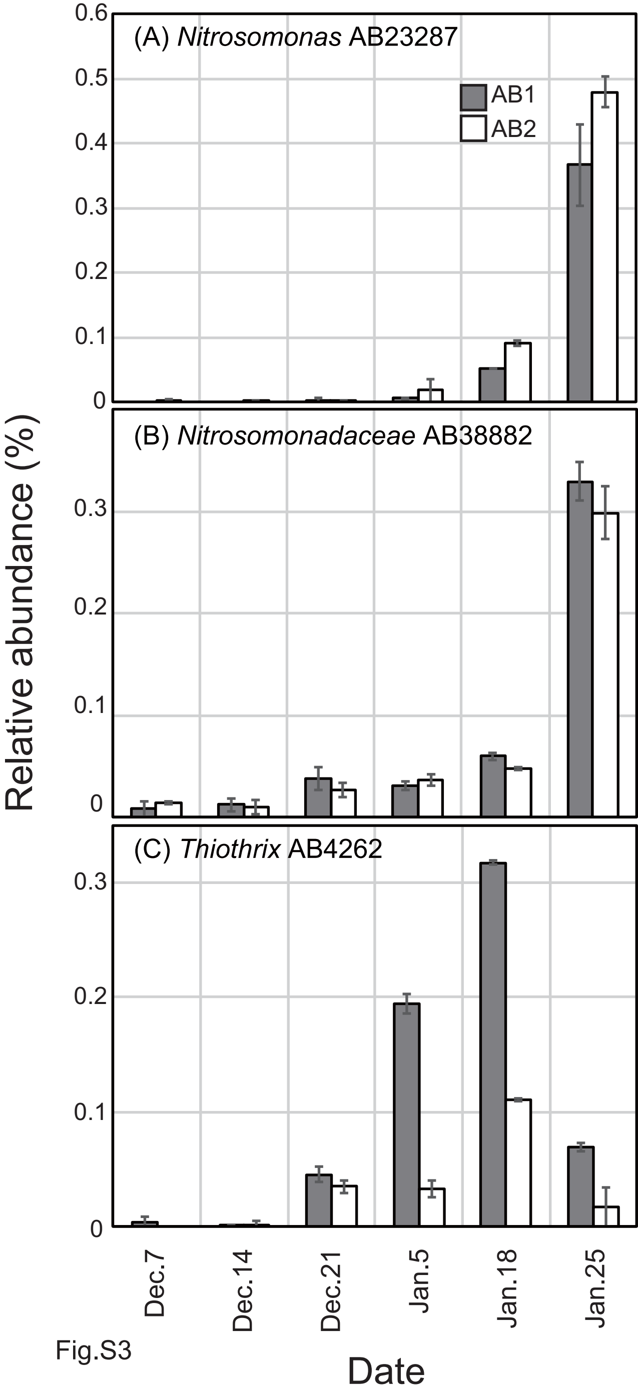
(A) Nitrosomonas AB23287
AB1
AB2
(B) Nitrosomonadaceae AB38882
(C) Thiothrix AB4262
0.6
0.5
0.4
0.3
0.2
0.1
0
Relative abundance (%)
Dec.7
Dec.14
Dec.21
Jan.5
Jan.18
Jan.25
Date

Fig.S3

Fig.S4

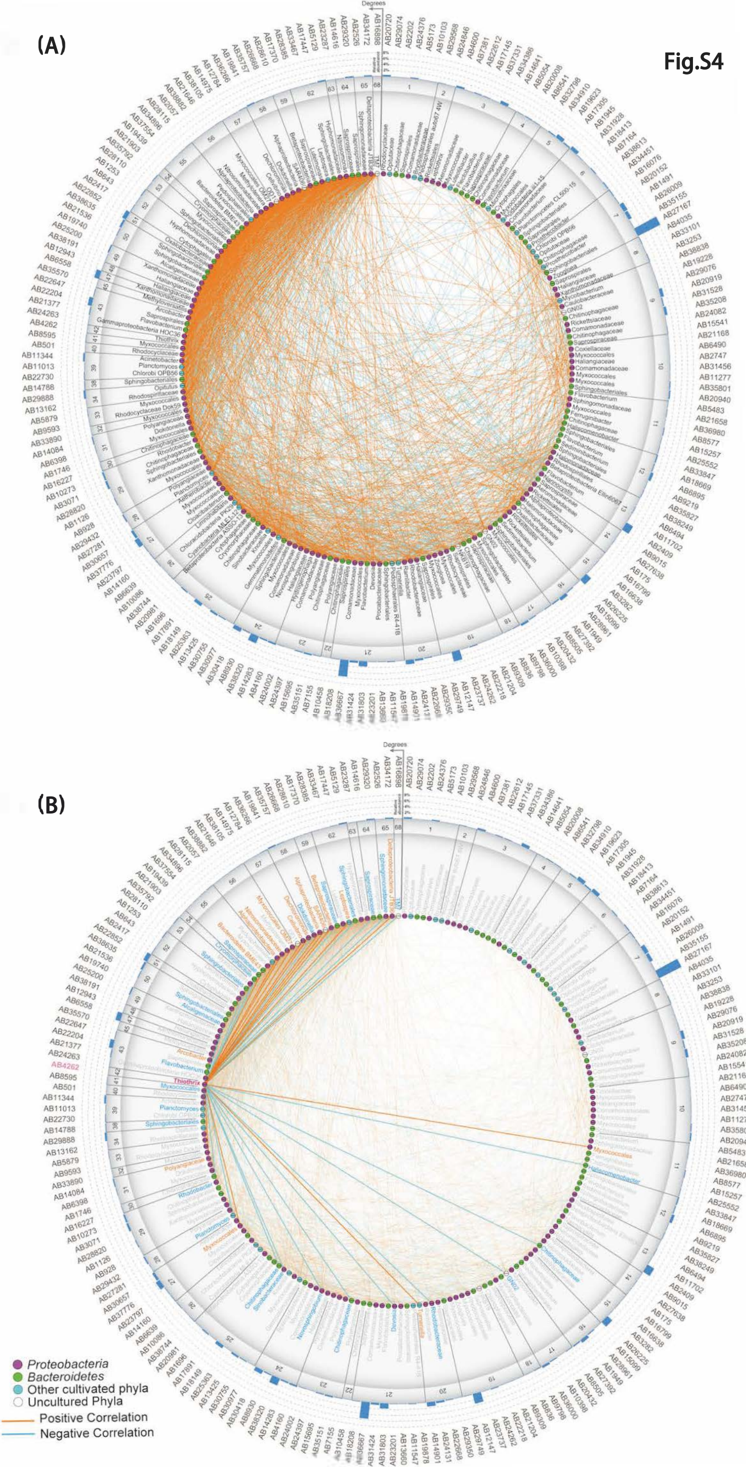

(A)
(B)
Proteobacteria
Bacteroidetes
Other cultivated phyla
Uncultured Phyla
Positive Correlation
Negative Correlation

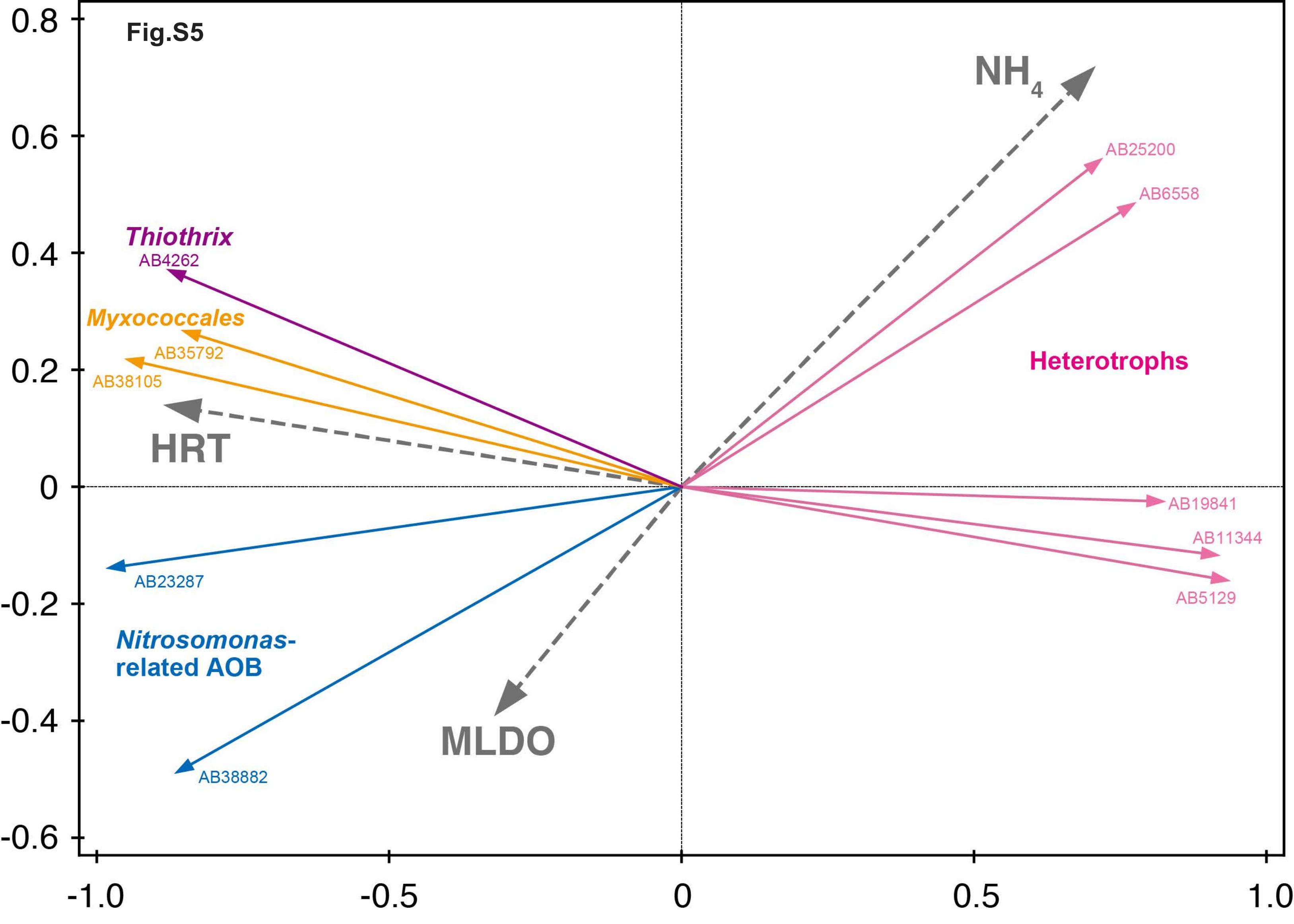

Fig.S5
NH4
AB25200
AB6558
Thiothrix
AB4262
Myxococcales
AB35792
AB38105
HRT
Heterotrophs
AB19841
AB11344
AB5129
AB23287
Nitrosomonas-
related AOB
MLDO
AB38882
0.8
0.6
0.4
0.2
0
-0.2
-0.4
-0.6
-1.0
-0.5
0
0.5
1.0

Supplemental Table S1. iTag sequencing results of 16S rRNA genes and observed diversity indexes

Tank ID	Sampling date	Read numbers	OTU numbers	Good's Coverage	Chao1 index
AB1	Dec. 7	69149	3435	0.97	9784
		84055	3796	0.972	11010
	Dec. 14	88319	3773	0.974	10551
		74963	3492	0.972	9335
	Dec. 21	90680	3662	0.976	10064
		80909	3667	0.973	10161
	Jan. 5	71415	3477	0.971	8996
		74670	4115	0.965	11687
	Jan. 18	90101	4014	0.973	11397
		85515	3893	0.972	10407
	Jan .25	98291	4280	0.973	11315
		88647	4022	0.973	11078
AB2	Dec. 7	80475	3773	0.973	9284
		87567	4425	0.97	10936
	Dec. 14	70844	3803	0.969	9881
		78698	3805	0.972	9987
	Dec. 21	84004	3954	0.972	10265
		96132	4370	0.973	11370
	Jan. 5	84044	4020	0.971	10538
		80819	3803	0.972	9890
	Jan. 18	102755	4087	0.976	11188
		80003	3663	0.973	9124
	Jan. 25	91760	4340	0.971	11914
		96248	4422	0.972	11776

Supplemental Table S2. Microbial community composition of activated sludge tanks AB1 and AB2.

#OTU ID	Number of degrees in network	Taxonomy							% of total dataset	Relative abundace in AB1 (%)												Relative abundace in AB2 (%)											
										Dec.7		Dec.14		Dec.21		Jan.5		Jan.18		Jan.25		Dec.7		Dec.14		Dec.21		Jan.5		Jan.18		Jan.25	
		Domain	Phylum	Class	Order	Family	Genus	Species		1	2	1	2	1	2	1	2	1	2	1	2	1	2	1	2	1	2	1	2	1	2	1	2
AB16898	68	Bacteria	TM7	SC3					0.10	0.21	0.20	0.52	0.41	0.13	0.10	0.01	0.01	0.00	0.00	0.00	0.00	0.12	0.16	0.14	0.17	0.03	0.05	0.00	0.01	0.01	0.02	0.03	0.01
AB34172	65	Bacteria	Proteobacteria	Deltaproteobacteria	Sva0853	JTB36			0.40	0.00	0.01	0.00	0.00	0.00	0.02	0.06	0.11	1.64	2.62	0.45	0.48	0.00	0.00	0.00	0.00	0.00	0.00	0.05	0.06	1.92	1.86	0.09	0.13
AB2526	65	Bacteria	Proteobacteria	Alphaproteobacteria	Sphingomonadales	Sphingomonadaceae			0.09	0.20	0.23	0.21	0.24	0.12	0.13	0.02	0.02	0.01	0.02	0.03	0.01	0.15	0.16	0.08	0.10	0.09	0.09	0.03	0.02	0.03	0.02	0.03	0.03
AB29320	64	Bacteria	Bacteroidetes	[Saprospirae]	[Saprospirales]				0.42	0.05	0.05	0.01	0.01	0.09	0.08	0.42	0.56	0.73	0.78	0.97	0.88	0.12	0.05	0.11	0.05	0.10	0.11	0.57	0.66	0.55	0.76	1.17	1.08
AB14616	64	Bacteria	Bacteroidetes	[Saprospirae]	[Saprospirales]	Saprospiraceae			0.11	0.28	0.26	0.16	0.16	0.10	0.13	0.07	0.06	0.05	0.05	0.05	0.05	0.21	0.15	0.18	0.14	0.09	0.09	0.09	0.08	0.02	0.05	0.07	0.05
**AB23287**	**63**	**Bacteria**	**Proteobacteria**	**Betaproteobacteria**	**Nitrosomonadales**	**Nitrosomonadaceae**	**Nitrosomonas**	**oligotropha**	**0.08**	**0.00**	**0.00**	**0.00**	**0.00**	**0.00**	**0.00**	**0.01**	**0.01**	**0.05**	**0.05**	**0.32**	**0.41**	**0.00**	**0.00**	**0.00**	**0.00**	**0.00**	**0.00**	**0.01**	**0.03**	**0.09**	**0.09**	**0.46**	**0.50**
AB5129	62	Bacteria	Proteobacteria	Alphaproteobacteria	Rhodobacterales	Hyphomonadaceae			0.44	0.81	0.78	0.75	0.70	0.60	0.61	0.21	0.29	0.28	0.32	0.32	0.31	0.51	0.50	0.53	0.43	0.54	0.45	0.36	0.29	0.23	0.22	0.22	0.24
AB17447	62	Bacteria	Bacteroidetes	Sphingobacteriia	Sphingobacteriales				0.16	0.51	0.49	0.47	0.43	0.17	0.23	0.04	0.06	0.03	0.06	0.07	0.05	0.15	0.12	0.13	0.12	0.12	0.14	0.09	0.08	0.05	0.02	0.08	0.06
AB28385	62	Bacteria	Proteobacteria	Gammaproteobacteria	Xanthomonadales	Xanthomonadaceae	Luteimonas		0.09	0.17	0.20	0.13	0.13	0.09	0.12	0.07	0.04	0.04	0.06	0.02	0.02	0.12	0.16	0.16	0.13	0.15	0.17	0.09	0.09	0.02	0.02	0.03	0.02
AB33467	62	Bacteria	Spirochaetes	[Leptospirae]	[Leptospirales]	Leptospiraceae	Leptospira		0.09	0.00	0.00	0.00	0.00	0.01	0.00	0.02	0.04	0.15	0.25	0.49	0.38	0.01	0.00	0.01	0.00	0.02	0.01	0.02	0.02	0.09	0.19	0.26	0.23
AB17370	59	Bacteria	Bacteroidetes	[Saprospirae]	[Saprospirales]	Saprospiraceae			0.09	0.14	0.12	0.07	0.06	0.02	0.03	0.02	0.02	0.01	0.00	0.01	0.01	0.21	0.29	0.25	0.24	0.18	0.15	0.06	0.04	0.02	0.02	0.05	0.02
AB28610	59	Bacteria	Proteobacteria	Betaproteobacteria					0.08	0.04	0.05	0.03	0.01	0.08	0.07	0.08	0.08	0.14	0.15	0.10	0.11	0.08	0.04	0.06	0.03	0.03	0.06	0.09	0.08	0.12	0.10	0.15	0.14
AB19841	58	Bacteria	Proteobacteria	Gammaproteobacteria	Xanthomonadales	Xanthomonadaceae	Dokdonella		0.43	0.70	0.68	0.58	0.63	0.42	0.38	0.23	0.16	0.21	0.19	0.13	0.16	0.62	0.69	0.66	0.71	0.48	0.80	0.35	0.39	0.26	0.25	0.41	0.32
AB35757	58	Bacteria	Proteobacteria	Alphaproteobacteria					0.21	0.03	0.02	0.03	0.04	0.17	0.13	0.32	0.28	0.51	0.66	0.39	0.46	0.02	0.04	0.03	0.01	0.07	0.07	0.09	0.11	0.73	0.35	0.21	0.23
AB26668	58	Bacteria	SAR406	AB16	noFP_H7				0.09	0.01	0.01	0.01	0.00	0.11	0.07	0.10	0.15	0.24	0.26	0.22	0.22	0.02	0.00	0.02	0.01	0.04	0.05	0.07	0.07	0.19	0.20	0.09	0.10
AB36266	57	Bacteria	Proteobacteria	Betaproteobacteria	Rhodocyclales	Rhodocyclaceae	Dechloromonas		0.20	0.11	0.12	0.12	0.11	0.07	0.12	0.29	0.30	0.39	0.32	0.33	0.41	0.07	0.04	0.09	0.07	0.11	0.12	0.21	0.19	0.24	0.28	0.28	0.34
AB14975	57	Bacteria	OD1	ABY1					0.09	0.24	0.27	0.26	0.25	0.15	0.20	0.05	0.05	0.02	0.01	0.02	0.01	0.03	0.11	0.05	0.10	0.11	0.11	0.03	0.03	0.00	0.00	0.01	0.01
AB12784	57	Bacteria	Proteobacteria	Gammaproteobacteria	Alteromonadales	Alteromonadaceae	Cellvibrio		0.09	0.02	0.01	0.00	0.01	0.02	0.01	0.03	0.07	0.18	0.21	0.18	0.20	0.01	0.01	0.00	0.02	0.07	0.08	0.07	0.05	0.26	0.31	0.12	0.18
AB38105	56	Bacteria	Proteobacteria	Deltaproteobacteria	Myxococcales	OM27			0.14	0.00	0.00	0.00	0.01	0.16	0.08	0.17	0.19	0.50	0.64	0.31	0.28	0.01	0.01	0.02	0.00	0.01	0.01	0.03	0.02	0.28	0.29	0.13	0.13
AB21646	56	Bacteria	Proteobacteria	Betaproteobacteria	Methylophilales	Methylophilaceae	Methylotenera	mobilis	0.09	0.20	0.16	0.10	0.12	0.07	0.09	0.10	0.10	0.06	0.04	0.00	0.00	0.09	0.15	0.12	0.19	0.14	0.14	0.05	0.08	0.04	0.03	0.00	0.02
**AB38882**	**56**	**Bacteria**	**Proteobacteria**	**Betaproteobacteria**	**Nitrosomonadales**	**Nitrosomonadaceae**			**0.08**	**0.00**	**0.01**	**0.02**	**0.01**	**0.03**	**0.05**	**0.03**	**0.03**	**0.06**	**0.06**	**0.34**	**0.32**	**0.01**	**0.01**	**0.01**	**0.02**	**0.02**	**0.03**	**0.03**	**0.04**	**0.05**	**0.05**	**0.32**	**0.28**
AB2057	56	Bacteria	Proteobacteria	Alphaproteobacteria					0.07	0.00	0.00	0.00	0.01	0.02	0.01	0.01	0.01	0.10	0.09	0.16	0.18	0.00	0.00	0.00	0.00	0.00	0.01	0.00	0.01	0.18	0.29	0.35	0.35
AB28115	55	Bacteria	Verrucomicrobia	[Pedosphaerae]	[Pedosphaerales]				0.16	0.02	0.02	0.01	0.02	0.07	0.04	0.09	0.09	0.25	0.31	0.87	0.76	0.02	0.03	0.06	0.03	0.02	0.02	0.02	0.05	0.17	0.12	0.33	0.34
AB34896	55	Bacteria	Proteobacteria	Deltaproteobacteria	Myxococcales				0.12	0.20	0.15	0.27	0.18	0.24	0.25	0.12	0.12	0.03	0.03	0.02	0.04	0.20	0.14	0.18	0.16	0.15	0.16	0.04	0.08	0.03	0.07	0.05	0.02
AB19439	55	Bacteria	Bacteroidetes	[Saprospirae]	[Saprospirales]	Saprospiraceae			0.08	0.07	0.08	0.14	0.19	0.08	0.06	0.06	0.07	0.03	0.01	0.04	0.03	0.22	0.14	0.22	0.14	0.05	0.06	0.06	0.04	0.03	0.02	0.04	0.02
AB37554	55	Bacteria	Bacteroidetes	BME43					0.08	0.03	0.03	0.04	0.04	0.07	0.07	0.15	0.09	0.18	0.19	0.20	0.16	0.04	0.03	0.01	0.03	0.03	0.04	0.06	0.09	0.07	0.07	0.09	0.08
AB21903	54	Bacteria	Bacteroidetes	Flavobacteriia	Flavobacteriales	Cryomorphaceae			0.08	0.10	0.14	0.14	0.10	0.09	0.09	0.03	0.04	0.08	0.06	0.03	0.02	0.13	0.12	0.10	0.11	0.10	0.08	0.04	0.05	0.06	0.06	0.03	0.05
AB35792	53	Bacteria	Proteobacteria	Deltaproteobacteria	Myxococcales				0.24	0.00	0.01	0.02	0.03	0.14	0.07	0.21	0.24	0.35	0.57	0.30	0.24	0.15	0.14	0.16	0.22	0.26	0.30	0.47	0.37	0.40	0.31	0.41	0.31
AB28110	53	Bacteria	Bacteroidetes	Sphingobacteriia	Sphingobacteriales				0.12	0.14	0.14	0.13	0.11	0.08	0.07	0.02	0.03	0.01	0.03	0.03	0.03	0.35	0.31	0.28	0.26	0.19	0.23	0.10	0.10	0.06	0.06	0.08	0.09
AB1253	52	Bacteria	Proteobacteria	Betaproteobacteria	Rhodocyclales	Rhodocyclaceae	Dechloromonas		0.26	0.14	0.15	0.04	0.07	0.07	0.08	0.15	0.16	0.51	0.60	0.57	0.56	0.07	0.23	0.08	0.05	0.08	0.07	0.19	0.18	0.52	0.52	0.49	0.65
AB2417	52	Bacteria	Bacteroidetes	Cytophagia	Cytophagales				0.17	0.16	0.12	0.08	0.11	0.08	0.08	0.10	0.17	0.29	0.35	0.19	0.24	0.03	0.03	0.03	0.03	0.04	0.04	0.13	0.17	0.44	0.52	0.30	0.30
AB643	52	Bacteria	Proteobacteria	Alphaproteobacteria	Rhodobacterales	Hyphomonadaceae			0.12	0.14	0.17	0.20	0.15	0.15	0.19	0.06	0.09	0.07	0.03	0.03	0.05	0.18	0.20	0.15	0.18	0.22	0.23	0.06	0.11	0.06	0.03	0.03	0.05
AB22852	51	Bacteria	Proteobacteria	Betaproteobacteria	Burkholderiales	Oxalobacteraceae			1.42	1.10	1.09	0.73	0.70	0.45	0.56	1.45	1.64	2.12	2.08	2.74	2.98	0.61	0.35	0.67	0.41	0.57	0.58	1.70	1.73	1.41	1.48	3.37	3.47
AB38635	50	Bacteria	Bacteroidetes	Sphingobacteriia	Sphingobacteriales				0.65	0.12	0.11	0.21	0.20	0.66	0.66	0.65	0.60	0.59	0.60	1.55	1.51	0.30	0.34	0.37	0.27	0.41	0.41	0.68	0.66	1.00	1.22	1.28	1.28
AB21536	50	Bacteria	Bacteroidetes	Sphingobacteriia	Sphingobacteriales				0.23	0.34	0.36	0.30	0.34	0.15	0.12	0.08	0.12	0.09	0.08	0.12	0.20	0.34	0.36	0.44	0.42	0.27	0.29	0.26	0.24	0.15	0.11	0.19	0.18
AB19740	50	Bacteria	Proteobacteria	Betaproteobacteria	Burkholderiales	Alcaligenaceae			0.07	0.11	0.13	0.12	0.14	0.04	0.05	0.02	0.02	0.05	0.05	0.03	0.03	0.11	0.11	0.11	0.09	0.12	0.14	0.07	0.06	0.04	0.02	0.03	0.02
AB25200	49	Bacteria	Proteobacteria	Gammaproteobacteria	Xanthomonadales	Xanthomonadaceae			0.40	0.82	0.77	0.40	0.30	0.40	0.40	0.38	0.34	0.28	0.25	0.08	0.09	0.52	0.60	0.57	0.60	0.62	0.66	0.31	0.28	0.21	0.31	0.17	0.16
AB38191	49	Bacteria	Proteobacteria	Deltaproteobacteria	Myxococcales	Haliangiaceae			0.11	0.17	0.17	0.21	0.12	0.16	0.16	0.07	0.10	0.04	0.07	0.05	0.05	0.14	0.08	0.15	0.15	0.15	0.14	0.10	0.05	0.08	0.06	0.05	0.07
AB12943	48	Bacteria	Proteobacteria	Deltaproteobacteria	Myxococcales	Haliangiaceae			0.09	0.11	0.15	0.16	0.14	0.18	0.19	0.09	0.05	0.05	0.07	0.04	0.03	0.13	0.08	0.08	0.10	0.13	0.15	0.06	0.04	0.06	0.04	0.04	0.04
AB6558	47	Bacteria	Proteobacteria	Gammaproteobacteria	Xanthomonadales	Xanthomonadaceae			1.65	2.10	2.24	1.84	1.25	1.10	1.33	0.97	0.75	0.73	0.86	0.33	0.36	2.75	3.17	2.98	3.26	3.29	3.15	1.96	1.65	1.07	1.33	0.56	0.54
AB35570	45	Bacteria	Proteobacteria	Betaproteobacteria	Rhodocyclales	Rhodocyclaceae	Methyloversatilis		0.07	0.06	0.05	0.02	0.03	0.07	0.06	0.07	0.06	0.11	0.10	0.08	0.10	0.08	0.04	0.05	0.04	0.09	0.08	0.09	0.10	0.07	0.14	0.09	0.11
AB22204	43	Bacteria	Bacteroidetes	[Saprospirae]	[Saprospirales]				1.34	1.11	1.09	0.83	0.83	1.06	0.97	1.34	1.46	1.52	1.28	1.42	1.47	0.91	0.79	0.99	0.86	1.36	1.18	2.18	2.23	1.48	1.62	2.22	1.92
AB21377	43	Bacteria	Bacteroidetes	Flavobacteriia	Flavobacteriales	Flavobacteriaceae	Flavobacterium	lutivivi	0.29	0.92	0.95	0.91	0.77	0.20	0.20	0.05	0.02	0.02	0.02	0.07	0.06	0.32	0.27	0.30	0.25	0.20	0.25	0.34	0.32	0.10	0.12	0.18	0.17
AB22647	43	Bacteria	Proteobacteria	Epsilonproteobacteria	Campylobacterales	Campylobacteraceae	Arcobacter	cryaerophilus	0.19	0.08	0.07	0.14	0.17	0.17	0.23	0.21	0.29	0.27	0.21	0.25	0.25	0.16	0.10	0.18	0.12	0.13	0.12	0.22	0.22	0.24	0.20	0.16	0.26
AB24263	43	Bacteria	Proteobacteria	Gammaproteobacteria	HOC36				0.09	0.02	0.01	0.01	0.00	0.06	0.05	0.05	0.05	0.13	0.11	0.03	0.03	0.04	0.02	0.02	0.02	0.05	0.07	0.14	0.11	0.34	0.38	0.13	0.16
**AB4262**	**42**	**Bacteria**	**Proteobacteria**	**Gammaproteobacteria**	**Thiotrichales**	**Thiotrichaceae**	**Thiothrix**		**0.07**	**0.00**	**0.01**	**0.00**	**0.00**	**0.04**	**0.05**	**0.20**	**0.19**	**0.32**	**0.32**	**0.07**	**0.07**	**0.00**	**0.00**	**0.00**	**0.00**	**0.04**	**0.03**	**0.04**	**0.03**	**0.11**	**0.11**	**0.03**	**0.01**
AB8595	41	Bacteria	Proteobacteria	Deltaproteobacteria	Myxococcales				0.16	0.30	0.34	0.51	0.40	0.30	0.35	0.02	0.02	0.01	0.01	0.02	0.02	0.18	0.17	0.23	0.12	0.09	0.10	0.05	0.05	0.10	0.09	0.14	0.16
AB501	40	Bacteria	Proteobacteria	Betaproteobacteria	Rhodocyclales	Rhodocyclaceae			0.09	0.01	0.01	0.01	0.01	0.05	0.04	0.09	0.10	0.12	0.12	0.14	0.16	0.12	0.08	0.12	0.08	0.12	0.10	0.07	0.10	0.15	0.11	0.12	0.17
AB11344	39	Bacteria	Proteobacteria	Gammaproteobacteria	Pseudomonadales	Moraxellaceae	Acinetobacter		0.55	1.13	0.91	0.88	0.90	0.48	0.49	0.48	0.52	0.50	0.47	0.46	0.34	0.50	0.60	0.63	0.65	0.40	0.53	0.45	0.48	0.39	0.26	0.38	0.41
AB22730	39	Bacteria	Chlorobi	OPB56					0.24	0.03	0.03	0.01	0.01	0.11	0.09	0.45	0.46	0.47	0.34	0.34	0.33	0.20	0.23	0.19	0.22	0.19	0.17	0.16	0.19	0.34	0.40	0.38	0.35
AB11013	39	Bacteria	Planctomycetes	Planctomycetia	Planctomycetales	Planctomycetaceae	Planctomyces		0.12	0.11	0.14	0.15	0.23	0.15	0.10	0.08	0.08	0.05	0.05	0.09	0.06	0.12	0.16	0.13	0.16	0.14	0.16	0.10	0.10	0.09	0.05	0.14	0.13
AB14788	38	Bacteria	Bacteroidetes	Sphingobacteriia	Sphingobacteriales				0.24	0.31	0.32	0.35	0.34	0.29	0.26	0.15	0.18	0.22	0.19	0.32	0.27	0.36	0.33	0.28	0.32	0.23	0.28	0.15	0.16	0.11	0.12	0.17	0.16
AB29888	34	Bacteria	Verrucomicrobia	Opitutae	Opitutales	Opitutaceae	Opitutus		0.65	0.23	0.31	0.24	0.21	0.55	0.47	0.84	0.87	0.35	0.54	1.23	1.26	0.79	0.52	0.74	0.45	0.33	0.32	0.69	0.88	0.85	0.70	1.04	1.27
AB13162	34	Bacteria	Proteobacteria	Alphaproteobacteria	Rhodospirillales	Rhodospirillaceae			0.09	0.12	0.09	0.10	0.19	0.09	0.10	0.04	0.06	0.10	0.09	0.08	0.08	0.16	0.13	0.13	0.11	0.10	0.11	0.07	0.07	0.06	0.08	0.06	0.05
AB5879	33	Bacteria	Proteobacteria	Deltaproteobacteria	Myxococcales				0.15	0.32	0.25	0.21	0.15	0.22	0.21	0.10	0.07	0.02	0.03	0.01	0.00	0.23	0.14	0.21	0.19	0.19	0.25	0.16	0.13	0.14	0.16	0.20	0.12
AB9593	33	Bacteria	Proteobacteria	Betaproteobacteria	Rhodocyclales	Rhodocyclaceae	Dok59		0.10	0.05	0.04	0.04	0.04	0.06	0.04	0.07	0.10	0.13	0.12	0.10	0.15	0.10	0.07	0.10	0.11	0.09	0.13	0.10	0.09	0.13	0.12	0.15	0.20
AB33890	32	Bacteria	Proteobacteria	Deltaproteobacteria	Myxococcales				0.09	0.05	0.04	0.13	0.09	0.08	0.07	0.08	0.07	0.05	0.07	0.05	0.05	0.21	0.22	0.21	0.21	0.09	0.08	0.03	0.05	0.03	0.03	0.02	0.02
AB14084	31	Bacteria	Proteobacteria	Deltaproteobacteria	Myxococcales	Polyangiaceae			0.33	0.29	0.22	0.39	0.29	0.82	0.81	0.72	0.59	0.87	0.91	0.30	0.32	0.05	0.04	0.06	0.06	0.10	0.12	0.13	0.15	0.23	0.20	0.14	0.13
AB6398	31	Bacteria	Proteobacteria	Gammaproteobacteria	Xanthomonadales	Xanthomonadaceae	Dokdonella		0.16	0.12	0.16	0.09	0.08	0.19	0.23	0.26	0.19	0.06	0.08	0.04	0.07	0.28	0.24	0.35	0.29	0.24	0.34	0.12	0.12	0.04	0.05	0.07	0.06
AB1746	31	Bacteria	Proteobacteria	Deltaproteobacteria	Myxococcales				0.07	0.07	0.07	0.06	0.06	0.11	0.12	0.04	0.05	0.05	0.04	0.03	0.02	0.07	0.08	0.09	0.11	0.09	0.13	0.06	0.03	0.07	0.06	0.03	0.04
AB16227	30	Bacteria	Bacteroidetes	[Saprospirae]	[Saprospirales]	Chitinophagaceae			0.17	0.25	0.25	0.53	0.45	0.39	0.33	0.12	0.12	0.10	0.09	0.19	0.13	0.14	0.16	0.14	0.10	0.11	0.07	0.05	0.04	0.06	0.06	0.08	0.09
AB3071	29	Bacteria	Bacteroidetes	[Saprospirae]	[Saprospirales]	Chitinophagaceae			0.64	0.89	0.87	1.76	2.06	1.53	1.35	0.45	0.37	0.28	0.24	0.49	0.47	0.48	0.47	0.46	0.52	0.39	0.48	0.33	0.27	0.16	0.23	0.50	0.42
AB10273	29	Bacteria	Proteobacteria	Alphaproteobacteria	Rhodobacterales	Rhodobacteraceae	Rhodobacter		0.50	0.65	0.60	0.53	0.68	0.51	0.47	0.53	0.45	0.36	0.36	0.39	0.38	0.63	0.56	0.72	0.52	0.44	0.48	0.52	0.49	0.44	0.33	0.51	0.52
AB1126	29	Bacteria	Proteobacteria	Gammaproteobacteria	Xanthomonadales	Xanthomonadaceae			0.25	0.02	0.03	0.04	0.06	0.05	0.04	0.04	0.05	0.05	0.07	0.12	0.08	0.43	0.51	0.53	0.72	0.52	0.62	0.43	0.46	0.23	0.23	0.34	0.24
AB28820	29	Bacteria	Bacteroidetes	Sphingobacteriia	Sphingobacteriales				0.08	0.03	0.05	0.01	0.02	0.02	0.02	0.20	0.22	0.09	0.08	0.09	0.13	0.08	0.05	0.07	0.08	0.06	0.05	0.06	0.06	0.06	0.07	0.10	0.13
AB928	29	Bacteria	Proteobacteria	Deltaproteobacteria	Myxococcales				0.07	0.02	0.01	0.01	0.01	0.02	0.04	0.08	0.04	0.06	0.05	0.04	0.05	0.07	0.08	0.05	0.09	0.10	0.10	0.12	0.08	0.16	0.11	0.11	0.14
AB29432	28	Bacteria	Proteobacteria	Deltaproteobacteria	Myxococcales	Polyangiaceae			0.40	0.34	0.25	0.41	0.33	0.68	0.78	0.95	0.91	1.01	0.96	0.56	0.49	0.21	0.10	0.15	0.08	0.14	0.12	0.16	0.17	0.20	0.27	0.14	0.16
AB27281	28	Bacteria	Planctomycetes	Planctomycetia	Planctomycetales	Planctomycetaceae	Planctomyces		0.08	0.15	0.10	0.08	0.08	0.08	0.08	0.04	0.05	0.04	0.03	0.09	0.08	0.14	0.11	0.14	0.10	0.09	0.10	0.02	0.04	0.02	0.02	0.06	0.08
AB37776	27	Bacteria	Proteobacteria	Deltaproteobacteria	Myxococcales				1.55	1.47	1.36	1.43	1.35	2.64	2.87	2.58	2.39	2.87	3.31	2.53	2.24	0.52	0.48	0.42	0.51	1.00	1.14	0.80	0.74	1.53	1.25	0.87	0.92
AB30657	27	Bacteria	Proteobacteria	Deltaproteobacteria	Myxococcales	Polyangiaceae	Aetherobacter	fasciculatus	0.81	1.67	1.60	1.55	1.37	2.39	2.33	1.11	1.06	1.15	1.10	0.44	0.37	0.23	0.14	0.19	0.14	0.23	0.20	0.27	0.30	0.50	0.50	0.27	0.31
AB14160	27	Bacteria	Bacteroidetes	Flavobacteriia	Flavobacteriales	[Weeksellaceae]	Cloacibacterium		0.19	0.18	0.12	0.50	0.48	0.30	0.25	0.18	0.23	0.11	0.09	0.15	0.13	0.19	0.28	0.14	0.19	0.23	0.20	0.10	0.13	0.14	0.08	0.09	0.08
AB23797	27	Bacteria	Proteobacteria	Deltaproteobacteria	Myxococcales				0.18	0.15	0.10	0.13	0.14	0.27	0.16	0.22	0.20	0.11	0.16	0.08	0.06	0.24	0.24	0.35	0.30	0.37	0.38	0.17	0.12	0.12	0.12	0.08	0.03
AB6639	26	Bacteria	Proteobacteria	Betaproteobacteria	Burkholderiales	Comamonadaceae	Limnohabitans		0.19	0.12	0.14	0.15	0.14	0.16	0.14	0.28	0.22	0.20	0.19	0.11	0.14	0.16	0.18	0.17	0.23	0.23	0.25	0.28	0.23	0.26	0.26	0.18	0.18
AB38744	26	Bacteria	Cyanobacteria	4C0d-2	MLE1-12				0.17	0.03	0.01	0.02	0.01	0.01	0.01	0.04	0.02	0.04	0.05	0.08	0.14	0.29	0.35	0.39	0.30	0.30	0.27	0.25	0.26	0.30	0.16	0.31	0.33
AB10086	26	Bacteria	Acidobacteria	[Chloracidobacteria]	PK29				0.08	0.04	0.03	0.02	0.03	0.03	0.02	0.03	0.03	0.02	0.02	0.03	0.03	0.27	0.28	0.25	0.19	0.17	0.09	0.12	0.09	0.03	0.04	0.05	0.05
AB18149	25	Bacteria	Bacteroidetes	[Saprospirae]	[Saprospirales]	Chitinophagaceae			1.04	0.92	0.88	0.69	0.70	0.49	0.41	0.52	0.53	0.42	0.37	1.22	1.01	1.63	1.65	1.55	1.65	1.58	1.56	1.27	1.48	0.66	0.67	1.61	1.57
AB13425	25	Bacteria	Actinobacteria	Actinobacteria	Actinomycetales	Intrasporangiaceae	Knoellia	subterranea	0.58	0.75	0.53	0.58	0.49	0.41	0.49	0.42	0.35	0.74	0.82	0.58	0.72	0.35	0.17	0.20	0.25	0.39	0.41	0.49	0.47	1.36	1.23	0.83	1.00
AB1696	25	Bacteria	Bacteroidetes	Cytophagia	Cytophagales	Cytophagaceae			0.37	0.86	0.75	0.75	0.73	0.46	0.48	0.31	0.35	0.22	0.23	0.62	0.55	0.32	0.27	0.33	0.22	0.24	0.20	0.17	0.19	0.06	0.10	0.25	0.24
AB30755	25	Bacteria	Proteobacteria	Deltaproteobacteria	Myxococcales				0.28	0.50	0.47	0.31	0.26	0.68	0.65	0.51	0.48	0.29	0.22	0.20	0.20	0.13	0.16	0.10	0.13	0.20	0.18	0.17	0.13	0.21	0.15	0.14	0.15
AB20981	25	Bacteria	Proteobacteria	Betaproteobacteria	ASSO-13				0.11	0.15	0.15	0.19	0.19	0.22	0.20	0.26	0.19	0.29	0.33	0.10	0.10	0.02	0.04	0.03	0.02	0.02	0.04	0.04	0.03	0.06	0.04	0.02	0.04
AB17891	25	Bacteria	Bacteroidetes	Flavobacteriia	Flavobacteriales	Cryomorphaceae			0.09	0.19	0.19	0.24	0.18	0.16	0.17	0.06	0.08	0.06	0.06	0.13	0.13	0.05	0.04	0.07	0.06	0.05	0.06	0.03	0.03	0.02	0.03	0.06	0.05
AB30977	25	Bacteria	Gemmatimonadetes	Gemmatimonadetes					0.07	0.17	0.16	0.13	0.09	0.10	0.15	0.09	0.06	0.06	0.05	0.03	0.03	0.07	0.04	0.07	0.05	0.06	0.04	0.04	0.05	0.02	0.04	0.04	0.03
AB25363	25	Bacteria	Proteobacteria	Gammaproteobacteria	Xanthomonadales	Sinobacteraceae			0.07	0.08	0.08	0.09	0.07	0.05	0.06	0.03	0.04	0.04	0.05	0.07	0.06	0.09	0.10	0.12	0.09	0.08	0.08	0.04	0.06	0.03	0.04	0.08	0.08
AB38320	24	Bacteria	Proteobacteria	Betaproteobacteria	Burkholderiales	Comamonadaceae			2.53	3.70	3.80	3.12	2.36	2.87	3.18	2.48	2.22	3.86	3.76	2.44	2.54	1.99	1.23	1.45	1.35	1.83	1.82	2.05	2.02	2.57	3.07	2.45	2.53
AB30418	24	Bacteria	Bacteroidetes	Sphingobacteriia	Sphingobacteriales				1.34	0.44	0.36	0.43	0.55	1.32	1.18	1.71	1.98	0.90	0.91	2.52	2.40	1.63	1.06	1.66	1.08	0.92	0.79	0.97	1.06	2.26	1.82	2.17	1.97
AB4160	24	Bacteria	Proteobacteria	Deltaproteobacteria	Myxococcales	Haliangiaceae			0.51	0.82	0.65	0.92	0.79	1.55	1.57	0.40	0.38	0.19	0.18	0.08	0.06	0.27	0.33	0.26	0.36	0.67	0.66	0.44	0.36	0.49	0.45	0.19	0.22
AB14283	24	Bacteria	Proteobacteria	Alphaproteobacteria	Sphingomonadales	Sphingomonadaceae	Novosphingobium		0.41	0.42	0.49	0.56	0.51	0.39	0.39	0.17	0.31	0.41	0.30	0.31	0.32	0.40	0.57	0.49	0.58	0.57	0.64	0.31	0.25	0.33	0.33	0.46	0.42
AB8930	24	Bacteria	Proteobacteria	Deltaproteobacteria	Myxococcales				0.31	0.03	0.07	0.06	0.05	0.16	0.15	0.40	0.44	0.38	0.43	0.26	0.28	0.28	0.32	0.20	0.40	0.53	0.53	0.40	0.40	0.44	0.52	0.37	0.34
AB35151	23	Bacteria	Bacteroidetes	[Saprospirae]	[Saprospirales]	Chitinophagaceae			0.44	0.35	0.32	0.47	0.43	0.23	0.23	0.20	0.21	0.22	0.14	0.31	0.27	0.36	0.69	0.54	0.72	0.71	0.56	0.76	0.68	0.31	0.35	0.78	0.76
AB15695	23	Bacteria	Proteobacteria	Deltaproteobacteria	Myxococcales	Polyangiaceae			0.30	0.48	0.50	0.58	0.49	0.78	0.73	0.73	0.60	0.49	0.44	0.21	0.22	0.14	0.06	0.13	0.06	0.09	0.09	0.07	0.06	0.06	0.09	0.05	0.05
AB7155	23	Bacteria	Proteobacteria	Deltaproteobacteria	Myxococcales	Polyangiaceae			0.20	0.21	0.20	0.19	0.17	0.44	0.44	0.41	0.36	0.41	0.37	0.13	0.13	0.11	0.11	0.11	0.10	0.13	0.14	0.12	0.12	0.15	0.15	0.06	0.05
AB24002	23	Bacteria	Proteobacteria	Betaproteobacteria	Burkholderiales	Comamonadaceae	Hydrogenophaga		0.16	0.01	0.02	0.05	0.03	0.10	0.18	0.51	0.48	0.12	0.13	0.06	0.06	0.16	0.24	0.15	0.34	0.24	0.22	0.16	0.19	0.14	0.15	0.06	0.08
AB24397	23	Bacteria	Proteobacteria	Betaproteobacteria	Burkholderiales	Comamonadaceae			0.07	0.01	0.01	0.01	0.01	0.03	0.02	0.15	0.12	0.02	0.02	0.00	0.01	0.17	0.21	0.13	0.22	0.20	0.17	0.08	0.05	0.03	0.01	0.01	0.01
AB10458	22	Bacteria	Bacteroidetes	[Saprospirae]	[Saprospirales]	Chitinophagaceae			0.07	0.10	0.11	0.08	0.09	0.16	0.11	0.07	0.09	0.07	0.11	0.12	0.08	0.02	0.05	0.07	0.05	0.04	0.05	0.03	0.02	0.03	0.02	0.04	0.03
AB36667	21	Bacteria	Proteobacteria	Betaproteobacteria	Burkholderiales	Comamonadaceae	Rhodoferax		5.66	4.20	4.40	4.84	4.39	4.33	4.32	5.97	5.93	6.70	6.08	4.85	5.26	5.02	5.92	4.45	6.39	6.48	6.80	7.54	7.59	6.65	6.67	5.19	5.84
AB31803	21	Bacteria	Bacteroidetes	Flavobacteriia	Flavobacteriales	Flavobacteriaceae	Flavobacterium		1.76	2.62	3.11	5.12	5.22	2.75	2.88	0.40	0.41	1.02	0.80	2.50	2.28	1.28	1.52	1.49	1.61	1.69	1.68	0.72	0.83	0.36	0.36	0.83	0.81
AB31424	21	Bacteria	Proteobacteria	Deltaproteobacteria	Myxococcales				0.66	0.14	0.11	0.33	0.29	0.51	0.48	0.54	0.49	0.74	0.91	0.65	0.67	0.77	0.88	0.67	0.88	1.23	1.02	0.95	0.79	0.77	0.74	0.57	0.61
AB11547	21	Bacteria	Bacteroidetes	Sphingobacteriia	Sphingobacteriales				0.25	0.05	0.07	0.26	0.27	0.78	0.97	0.52	0.63	0.34	0.32	0.46	0.42	0.01	0.01	0.01	0.02	0.01	0.01	0.11	0.12	0.13	0.15	0.21	0.18
AB18208	21	Bacteria	Bacteroidetes	[Saprospirae]	[Saprospirales]				0.18	0.06	0.09	0.10	0.09	0.19	0.22	0.31	0.27	0.13	0.12	0.28	0.23	0.15	0.10	0.16	0.16	0.10	0.12	0.21	0.22	0.15	0.24	0.29	0.27
AB23201	21	Bacteria	Proteobacteria	Alphaproteobacteria	Rhizobiales	Hyphomicrobiaceae	Devosia		0.14	0.18	0.19	0.20	0.18	0.10	0.14	0.07	0.07	0.08	0.11	0.15	0.14	0.20	0.23	0.17	0.19	0.14	0.16	0.10	0.08	0.10	0.12	0.20	0.16
AB13660	21	Bacteria	Proteobacteria	Betaproteobacteria	Procabacteriales	Procabacteriaceae			0.12	0.35	0.36	0.06	0.06	0.06	0.11	0.08	0.09	0.17	0.20	0.17	0.17	0.02	0.02	0.02	0.01	0.06	0.09	0.15	0.12	0.18	0.17	0.12	0.13
AB19878	21	Bacteria	Verrucomicrobia	[Pedosphaerae]	[Pedosphaerales]	R4-41B			0.09	0.03	0.04	0.01	0.01	0.01	0.02	0.04	0.05	0.05	0.05	0.18	0.19	0.18	0.14	0.21	0.09	0.04	0.04	0.10	0.12	0.14	0.07	0.21	0.19
AB14901	20	Bacteria	Spirochaetes	[Leptospirae]	[Leptospirales]	Leptospiraceae	Turneriella		1.44	0.82	0.79	0.74	0.63	2.06	1.96	1.84	2.22	2.60	2.23	1.33	1.26	1.08	1.26	1.08	0.96	1.52	1.54	1.43	1.61	1.72	1.58	1.23	1.07
AB24131	20	Bacteria	Proteobacteria	Betaproteobacteria	Burkholderiales	Comamonadaceae	Ramlibacter		0.64	0.20	0.19	0.21	0.20	0.66	0.66	0.97	1.10	0.50	0.38	0.37	0.36	1.01	1.00	0.97	1.11	0.80	0.90	0.68	0.78	0.72	0.70	0.46	0.49
AB22668	20	Bacteria	Proteobacteria	Alphaproteobacteria	Rhodobacterales	Rhodobacteraceae			0.26	0.29	0.33	0.31	0.36	0.23	0.26	0.11	0.15	0.14	0.11	0.22	0.21	0.33	0.28	0.40	0.27	0.25	0.29	0.24	0.22	0.26	0.26	0.39	0.43
AB29350	20	Bacteria	Proteobacteria	Deltaproteobacteria	Myxococcales	Haliangiaceae			0.11	0.03	0.06	0.05	0.06	0.08	0.05	0.09	0.07	0.03	0.04	0.08	0.09	0.17	0.11	0.12	0.11	0.10	0.10	0.24	0.16	0.18	0.18	0.25	0.25
AB12147	19	Bacteria	Proteobacteria	Deltaproteobacteria	Myxococcales				3.36	0.24	0.23	0.60	0.60	1.18	1.29	1.94	1.86	4.98	4.89	4.79	4.18	3.68	4.42	3.91	4.40	4.91	4.85	5.55	5.95	4.83	4.68	3.35	3.35
AB24262	19	Bacteria	Proteobacteria	Deltaproteobacteria	Myxococcales				0.29	0.44	0.34	0.19	0.19	0.44	0.45	0.21	0.22	0.24	0.22	0.27	0.38	0.13	0.07	0.12	0.06	0.11	0.10	0.21	0.20	0.49	0.50	0.68	0.73
AB29749	19	Bacteria	Bacteroidetes	[Saprospirae]	[Saprospirales]				0.14	0.09	0.12	0.01	0.01	0.01	0.03	0.15	0.24	0.12	0.09	0.12	0.09	0.03	0.06	0.04	0.05	0.09	0.10	0.46	0.52	0.18	0.24	0.29	0.25
AB23737	19	Bacteria	Proteobacteria	Betaproteobacteria	Rhodocyclales	Rhodocyclaceae	Zoogloea		0.11	0.09	0.09	0.08	0.06	0.05	0.04	0.08	0.07	0.07	0.07	0.08	0.08	0.15	0.12	0.13	0.16	0.15	0.13	0.17	0.14	0.09	0.12	0.15	0.17
AB21204	19	Bacteria	Bacteroidetes	[Saprospirae]	[Saprospirales]				0.10	0.01	0.03	0.10	0.16	0.07	0.04	0.06	0.05	0.05	0.06	0.06	0.09	0.22	0.17	0.23	0.22	0.11	0.11	0.11	0.14	0.07	0.08	0.12	0.09
AB9309	19	Bacteria	NKB19	TSBW08					0.10	0.18	0.26	0.13	0.13	0.09	0.09	0.05	0.05	0.11	0.11	0.09	0.11	0.04	0.05	0.04	0.06	0.08	0.07	0.07	0.10	0.13	0.11	0.10	0.11
AB22218	19	Bacteria	Proteobacteria	Betaproteobacteria	Rhodocyclales	Rhodocyclaceae			0.08	0.09	0.08	0.06	0.07	0.08	0.08	0.14	0.12	0.13	0.14	0.08	0.09	0.08	0.06	0.06	0.05	0.05	0.05	0.09	0.10	0.10	0.07	0.09	0.10
AB9798	18	Bacteria	Bacteroidetes	[Saprospirae]	[Saprospirales]	Saprospiraceae			0.23	0.37	0.34	0.34	0.36	0.20	0.17	0.25	0.24	0.13	0.11	0.31	0.34	0.38	0.28	0.39	0.21	0.16	0.13	0.14	0.13	0.06	0.06	0.20	0.23
AB836	18	Bacteria	Bacteroidetes	[Saprospirae]	[Saprospirales]	Chitinophagaceae			0.14	0.21	0.23	0.29	0.33	0.20	0.19	0.10	0.09	0.10	0.10	0.16	0.15	0.05	0.15	0.17	0.10	0.12	0.14	0.07	0.13	0.05	0.06	0.10	0.14

AB36000	17	Bacteria	Proteobacteria	Deltaproteobacteria	Myxococcales				0.71	0.86	0.96	0.86	0.65	0.44	0.39	0.73	0.54	0.41	0.51	0.33	0.23	0.88	0.91	0.91	1.11	1.01	1.18	0.77	0.74	0.83	0.98	0.46	0.41
AB10398	17	Bacteria	Bacteroidetes	Sphingobacteriia	Sphingobacteriales				0.28	0.21	0.17	0.09	0.13	0.06	0.10	0.15	0.11	0.16	0.19	0.42	0.33	0.41	0.27	0.30	0.32	0.49	0.42	0.44	0.52	0.17	0.22	0.54	0.49
AB8505	17	Bacteria	Proteobacteria	Deltaproteobacteria	Myxococcales				0.23	0.35	0.32	0.17	0.12	0.42	0.50	0.37	0.38	0.26	0.24	0.19	0.16	0.09	0.12	0.10	0.10	0.16	0.10	0.16	0.18	0.24	0.26	0.25	0.23
AB20432	17	Bacteria	GN02	BD1-5					0.07	0.10	0.14	0.14	0.18	0.10	0.12	0.02	0.03	0.02	0.03	0.04	0.05	0.09	0.05	0.09	0.04	0.03	0.04	0.07	0.10	0.04	0.04	0.05	0.05
AB1949	16	Bacteria	Bacteroidetes	[Saprospirae]	[Saprospirales]	Chitinophagaceae	Sediminibacterium		1.09	0.39	0.49	0.67	0.63	1.13	1.17	1.48	1.68	0.63	0.53	1.03	0.83	2.06	2.22	2.03	2.11	0.91	0.79	0.97	0.89	0.79	0.71	0.98	1.03
AB27392	16	Bacteria	Bacteroidetes	Sphingobacteriia	Sphingobacteriales				0.36	0.40	0.45	0.50	0.44	0.49	0.58	0.30	0.34	0.25	0.24	0.54	0.52	0.27	0.16	0.35	0.21	0.18	0.16	0.20	0.22	0.28	0.24	0.62	0.64
AB28961	16	Bacteria	Proteobacteria	Alphaproteobacteria	Rickettsiales				0.28	0.58	0.43	1.75	1.65	0.09	0.09	0.03	0.05	0.42	0.35	0.19	0.27	0.02	0.06	0.03	0.06	0.05	0.08	0.03	0.04	0.18	0.19	0.09	0.11
AB15099	16	Bacteria	Proteobacteria	Alphaproteobacteria	Rickettsiales				0.11	0.13	0.15	0.16	0.17	0.12	0.10	0.08	0.10	0.11	0.10	0.13	0.16	0.16	0.11	0.12	0.11	0.07	0.04	0.06	0.05	0.09	0.09	0.07	0.10
AB26225	15	Bacteria	Proteobacteria	Betaproteobacteria	Burkholderiales	Oxalobacteraceae			2.22	1.71	1.84	1.71	1.75	2.50	2.70	2.90	2.82	2.07	1.77	1.13	1.19	2.10	2.69	2.01	2.77	3.18	3.21	3.24	3.39	2.10	1.85	1.29	1.45
AB16638	15	Bacteria	Proteobacteria	Gammaproteobacteria	Oceanospirillales	Halomonadaceae			0.12	0.00	0.00	0.00	0.00	0.01	0.01	0.20	0.24	0.05	0.06	0.08	0.10	0.15	0.17	0.13	0.16	0.16	0.12	0.14	0.12	0.27	0.30	0.19	0.15
AB16799	15	Bacteria	Proteobacteria	Alphaproteobacteria					0.09	0.13	0.13	0.09	0.05	0.16	0.16	0.12	0.18	0.05	0.06	0.03	0.04	0.07	0.09	0.09	0.12	0.13	0.11	0.03	0.04	0.05	0.07	0.03	0.03
AB3282	15	Bacteria	Bacteroidetes	[Saprospirae]	[Saprospirales]	Chitinophagaceae			0.08	0.14	0.19	0.09	0.14	0.04	0.02	0.01	0.02	0.01	0.02	0.05	0.02	0.05	0.12	0.07	0.12	0.13	0.06	0.12	0.13	0.09	0.09	0.11	0.11
AB27638	14	Bacteria	Proteobacteria	Gammaproteobacteria	Aeromonadales	Aeromonadaceae			0.12	0.10	0.09	0.14	0.16	0.20	0.16	0.12	0.14	0.08	0.10	0.06	0.07	0.12	0.15	0.11	0.12	0.13	0.16	0.15	0.13	0.11	0.10	0.08	0.06
AB175	14	Bacteria	Proteobacteria	Alphaproteobacteria	Rickettsiales				0.09	0.02	0.04	0.07	0.05	0.04	0.05	0.03	0.03	0.12	0.07	0.17	0.11	0.16	0.18	0.19	0.09	0.12	0.12	0.17	0.17	0.07	0.08	0.08	0.07
AB9015	14	Bacteria	Bacteroidetes	[Saprospirae]	[Saprospirales]	Saprospiraceae			0.08	0.05	0.10	0.07	0.13	0.06	0.10	0.09	0.07	0.06	0.04	0.07	0.06	0.12	0.15	0.14	0.11	0.10	0.05	0.05	0.07	0.04	0.04	0.05	0.04
AB2409	13	Bacteria	Proteobacteria	Deltaproteobacteria	Myxococcales	Nannocystaceae	Nannocystis		2.64	4.53	3.77	3.92	3.12	1.80	1.58	0.77	0.75	1.73	1.78	0.99	0.99	2.19	3.19	1.91	3.20	3.83	4.00	3.54	2.97	5.80	3.93	1.55	1.58
AB6494	13	Bacteria	Proteobacteria	Betaproteobacteria	Ellin6067				0.18	0.03	0.02	0.02	0.03	0.03	0.03	0.09	0.07	0.06	0.06	0.09	0.08	0.46	0.67	0.44	0.66	0.49	0.68	0.06	0.04	0.08	0.05	0.09	0.09
AB11702	13	Bacteria	Bacteroidetes	Flavobacteriia	Flavobacteriales	Flavobacteriaceae	Flavobacterium		0.11	0.05	0.07	0.08	0.06	0.04	0.06	0.12	0.14	0.15	0.09	0.29	0.25	0.08	0.09	0.06	0.13	0.08	0.09	0.13	0.14	0.05	0.08	0.12	0.17
AB38249	13	Bacteria	Proteobacteria	Alphaproteobacteria	Rhodospirillales				0.07	0.02	0.02	0.05	0.09	0.04	0.03	0.03	0.02	0.03	0.02	0.09	0.08	0.18	0.15	0.16	0.14	0.05	0.05	0.08	0.05	0.04	0.03	0.07	0.06
AB6895	12	Bacteria	Bacteroidetes	[Saprospirae]	[Saprospirales]	Chitinophagaceae	Sediminibacterium		0.88	0.20	0.23	0.36	0.31	1.57	1.31	1.65	1.94	0.39	0.34	0.62	0.58	0.82	1.42	0.77	1.43	1.17	0.98	0.86	0.82	0.82	0.69	1.01	0.95
AB33847	12	Bacteria	Bacteroidetes	Sphingobacteriia	Sphingobacteriales				0.36	0.62	0.65	0.36	0.34	0.16	0.17	0.18	0.20	0.26	0.20	0.46	0.54	0.44	0.18	0.41	0.17	0.14	0.14	0.37	0.36	0.53	0.55	0.62	0.69
AB18669	12	Bacteria	Bacteroidetes	Flavobacteriia	Flavobacteriales	Flavobacteriaceae	Flavobacterium		0.27	0.36	0.36	0.38	0.42	0.17	0.20	0.06	0.07	0.26	0.26	0.84	0.75	0.25	0.19	0.31	0.21	0.18	0.13	0.18	0.18	0.09	0.13	0.29	0.27
AB35827	12	Bacteria	Proteobacteria	Gammaproteobacteria	Oceanospirillales	Halomonadaceae			0.19	0.12	0.10	0.08	0.06	0.22	0.24	0.36	0.44	0.06	0.12	0.05	0.04	0.46	0.35	0.37	0.36	0.19	0.25	0.20	0.26	0.06	0.10	0.03	0.05
AB9219	12	Bacteria	Bacteroidetes	Sphingobacteriia	Sphingobacteriales				0.12	0.16	0.10	0.19	0.21	0.18	0.19	0.12	0.13	0.11	0.11	0.17	0.12	0.11	0.09	0.13	0.07	0.12	0.06	0.09	0.11	0.08	0.07	0.10	0.10
AB8577	11	Bacteria	Bacteroidetes	[Saprospirae]	[Saprospirales]	Chitinophagaceae	Ferruginibacter	lapsinanis	0.27	0.12	0.11	0.12	0.16	0.29	0.26	0.41	0.26	0.17	0.20	0.37	0.28	0.20	0.41	0.24	0.45	0.48	0.36	0.33	0.31	0.17	0.13	0.33	0.30
AB25552	11	Bacteria	Bacteroidetes	[Saprospirae]	[Saprospirales]	Saprospiraceae	Haliscomenobacter		0.17	0.21	0.16	0.15	0.19	0.14	0.12	0.10	0.11	0.11	0.07	0.27	0.27	0.36	0.31	0.43	0.20	0.15	0.17	0.08	0.07	0.05	0.05	0.16	0.13
AB21658	11	Bacteria	Proteobacteria	Alphaproteobacteria	Sphingomonadales	Sphingomonadaceae			0.16	0.08	0.10	0.14	0.14	0.12	0.11	0.11	0.08	0.15	0.16	0.15	0.13	0.13	0.12	0.13	0.13	0.25	0.19	0.24	0.26	0.20	0.22	0.28	0.25
AB15257	11	Bacteria	Bacteroidetes	[Saprospirae]	[Saprospirales]	Chitinophagaceae			0.12	0.04	0.02	0.03	0.04	0.18	0.14	0.20	0.22	0.11	0.12	0.17	0.12	0.17	0.12	0.17	0.11	0.10	0.08	0.12	0.09	0.12	0.13	0.13	0.09
AB5483	11	Bacteria	Bacteroidetes	Flavobacteriia	Flavobacteriales	Flavobacteriaceae	Flavobacterium		0.11	0.02	0.01	0.08	0.10	0.30	0.27	0.13	0.13	0.07	0.05	0.10	0.12	0.08	0.15	0.12	0.15	0.14	0.10	0.14	0.13	0.03	0.06	0.10	0.07
AB36980	11	Bacteria	Proteobacteria	Deltaproteobacteria	Myxococcales				0.07	0.02	0.02	0.03	0.02	0.08	0.08	0.20	0.17	0.13	0.16	0.09	0.09	0.06	0.05	0.07	0.05	0.06	0.06	0.04	0.03	0.03	0.04	0.03	0.02
AB20940	10	Bacteria	Bacteroidetes	Sphingobacteriia	Sphingobacteriales				0.43	0.88	0.90	0.92	0.96	0.39	0.44	0.07	0.09	0.17	0.18	0.35	0.34	0.37	0.38	0.37	0.39	0.26	0.27	0.27	0.32	0.46	0.46	0.48	0.55
AB35801	10	Bacteria	Proteobacteria	Deltaproteobacteria	Myxococcales				0.30	0.14	0.12	0.26	0.21	0.48	0.44	0.48	0.35	0.31	0.41	0.18	0.14	0.37	0.36	0.33	0.41	0.44	0.57	0.17	0.19	0.29	0.23	0.14	0.17
AB31456	10	Bacteria	Proteobacteria	Betaproteobacteria	Burkholderiales	Comamonadaceae			0.12	0.00	0.00	0.00	0.00	0.07	0.12	0.11	0.12	0.15	0.12	0.15	0.14	0.11	0.22	0.13	0.20	0.24	0.22	0.08	0.06	0.15	0.16	0.14	0.18
AB11277	10	Bacteria	Proteobacteria	Deltaproteobacteria	Myxococcales				0.10	0.08	0.08	0.05	0.04	0.06	0.08	0.10	0.08	0.12	0.18	0.16	0.16	0.13	0.09	0.12	0.11	0.08	0.10	0.07	0.08	0.10	0.06	0.17	0.15
AB2747	10	Bacteria	Proteobacteria	Deltaproteobacteria	Myxococcales	Haliangiaceae			0.08	0.08	0.10	0.07	0.03	0.11	0.08	0.06	0.09	0.10	0.06	0.03	0.02	0.09	0.14	0.10	0.13	0.16	0.15	0.09	0.10	0.07	0.10	0.03	0.04
AB6490	10	Bacteria	Proteobacteria	Deltaproteobacteria	Myxococcales				0.08	0.05	0.05	0.04	0.04	0.09	0.11	0.17	0.15	0.10	0.08	0.08	0.09	0.11	0.06	0.08	0.06	0.07	0.04	0.09	0.06	0.08	0.09	0.10	0.10
AB21168	10	Bacteria	Proteobacteria	Gammaproteobacteria	Legionellales	Coxiellaceae			0.08	0.73	0.64	0.03	0.04	0.00	0.00	0.00	0.01	0.07	0.08	0.02	0.02	0.02	0.02	0.02	0.01	0.02	0.01	0.03	0.01	0.04	0.04	0.02	0.04
AB20919	9	Bacteria	Bacteroidetes	[Saprospirae]	[Saprospirales]	Chitinophagaceae			1.05	1.29	1.36	1.45	2.10	1.45	1.40	1.09	0.86	0.65	0.69	0.81	0.74	0.51	0.96	0.73	0.98	0.97	0.95	1.40	1.17	0.89	0.73	1.01	0.99
AB35208	9	Bacteria	Proteobacteria	Betaproteobacteria	Burkholderiales	Comamonadaceae			0.85	1.18	1.00	1.14	0.87	1.07	1.07	0.60	0.45	0.94	0.98	0.59	0.64	0.67	0.71	0.58	0.66	1.05	1.19	0.79	0.81	0.81	1.12	0.72	0.75
AB31528	9	Bacteria	Proteobacteria	Alphaproteobacteria	Rickettsiales	Rickettsiaceae			0.33	0.79	0.79	1.54	1.52	0.16	0.19	0.09	0.14	0.20	0.15	0.23	0.23	0.17	0.14	0.19	0.12	0.15	0.16	0.15	0.11	0.23	0.15	0.13	0.15
AB15541	9	Bacteria	Bacteroidetes	[Saprospirae]	[Saprospirales]	Saprospiraceae			0.16	0.25	0.25	0.18	0.22	0.08	0.16	0.19	0.19	0.10	0.11	0.24	0.22	0.25	0.15	0.27	0.21	0.11	0.07	0.09	0.11	0.07	0.06	0.14	0.14
AB29076	9	Bacteria	GN02	BD1-5					0.12	0.00	0.00	0.00	0.00	0.06	0.04	0.05	0.05	0.07	0.03	0.05	0.05	0.42	0.31	0.50	0.33	0.11	0.12	0.01	0.01	0.11	0.12	0.19	0.18
AB19228	9	Bacteria	Proteobacteria	Alphaproteobacteria	Caulobacterales	Caulobacteraceae			0.08	0.26	0.16	0.12	0.08	0.07	0.07	0.04	0.03	0.12	0.08	0.04	0.05	0.07	0.07	0.07	0.08	0.09	0.07	0.04	0.04	0.06	0.06	0.05	0.04
AB38838	9	Bacteria	Actinobacteria	Actinobacteria	Actinomycetales	Mycobacteriaceae	Mycobacterium		0.07	0.06	0.03	0.06	0.12	0.07	0.07	0.03	0.02	0.04	0.05	0.03	0.06	0.11	0.10	0.10	0.08	0.12	0.12	0.07	0.09	0.08	0.09	0.08	0.07
AB24082	9	Bacteria	Bacteroidetes	[Saprospirae]	[Saprospirales]	Chitinophagaceae			0.07	0.03	0.07	0.13	0.10	0.04	0.07	0.03	0.01	0.02	0.02	0.05	0.05	0.05	0.13	0.06	0.09	0.09	0.10	0.12	0.10	0.06	0.07	0.08	0.05
AB3253	8	Bacteria	Proteobacteria	Gammaproteobacteria	Xanthomonadales	Xanthomonadaceae			0.25	0.27	0.20	0.09	0.09	0.17	0.19	0.58	0.57	0.26	0.30	0.05	0.10	0.33	0.29	0.36	0.31	0.39	0.42	0.20	0.17	0.20	0.19	0.09	0.08
AB4035	8	Bacteria	Bacteroidetes	[Saprospirae]	[Saprospirales]				0.20	0.06	0.07	0.19	0.29	0.41	0.41	0.23	0.35	0.25	0.21	0.13	0.11	0.38	0.25	0.29	0.25	0.12	0.15	0.07	0.03	0.11	0.16	0.09	0.10
AB33101	8	Bacteria	Proteobacteria	Deltaproteobacteria	Myxococcales	Haliangiaceae			0.10	0.04	0.03	0.05	0.04	0.13	0.12	0.16	0.14	0.06	0.11	0.14	0.10	0.05	0.13	0.07	0.13	0.12	0.11	0.12	0.11	0.06	0.06	0.11	0.09
AB27167	7	Bacteria	Proteobacteria	Betaproteobacteria	Rhodocyclales	Rhodocyclaceae	Zoogloea		8.54	11.01	12.94	7.63	8.16	6.58	6.54	12.80	11.18	7.64	7.46	8.02	8.79	7.79	5.17	7.16	5.84	5.71	5.03	11.12	10.53	7.92	9.91	10.13	9.88
AB1491	7	Bacteria	Bacteroidetes	[Saprospirae]	[Saprospirales]	Chitinophagaceae			1.41	2.04	2.05	1.44	1.70	1.67	1.45	1.93	1.99	1.13	0.93	1.95	2.11	1.18	1.19	1.00	1.19	0.92	1.04	1.12	1.39	0.57	0.75	1.76	1.43
AB26009	7	Bacteria	Verrucomicrobia	Verrucomicrobiae	Verrucomicrobiales	Verrucomicrobiaceae	Prosthecobacter		1.10	0.27	0.32	0.31	0.35	1.57	1.33	1.20	1.38	0.83	0.81	0.74	0.79	1.78	1.56	1.64	1.40	1.17	1.23	1.22	1.42	1.90	1.47	0.80	0.89
AB16076	7	Bacteria	Chlorobi	OPB56					0.25	0.09	0.09	0.09	0.12	0.21	0.19	0.29	0.26	0.44	0.27	0.31	0.36	0.40	0.28	0.57	0.35	0.27	0.25	0.27	0.27	0.17	0.19	0.19	0.16
AB20152	7	Bacteria	Verrucomicrobia	Opitutae	Opitutales	Opitutaceae			0.17	0.03	0.03	0.31	0.41	0.65	0.67	0.21	0.26	0.09	0.06	0.09	0.06	0.08	0.10	0.12	0.15	0.15	0.13	0.06	0.11	0.08	0.08	0.07	0.08
AB35155	7	Bacteria	Bacteroidetes	Sphingobacteriia	Sphingobacteriales				0.13	0.30	0.33	0.30	0.30	0.11	0.11	0.12	0.12	0.05	0.05	0.10	0.06	0.10	0.06	0.10	0.08	0.03	0.02	0.15	0.10	0.14	0.11	0.16	0.14
AB1945	6	Bacteria	Proteobacteria	Betaproteobacteria	Burkholderiales	Comamonadaceae			1.22	1.35	1.36	1.31	1.16	1.28	1.34	1.44	1.33	0.97	0.94	0.49	0.54	0.90	1.35	0.85	1.56	2.11	2.17	1.54	1.59	1.11	1.15	0.68	0.72
AB34451	6	Bacteria	Verrucomicrobia	Verrucomicrobiae	Verrucomicrobiales	Verrucomicrobiaceae	Prosthecobacter	debontii	0.30	0.06	0.08	0.28	0.28	0.79	0.62	0.42	0.43	0.31	0.32	0.35	0.37	0.36	0.31	0.37	0.22	0.14	0.15	0.15	0.20	0.31	0.24	0.23	0.22
AB7164	6	Bacteria	Bacteroidetes	Sphingobacteriia	Sphingobacteriales				0.28	0.36	0.30	0.28	0.27	0.26	0.24	0.16	0.22	0.45	0.38	0.63	0.49	0.28	0.25	0.23	0.27	0.19	0.18	0.17	0.15	0.29	0.22	0.20	0.24
AB31928	6	Bacteria	Bacteroidetes	Flavobacteriia	Flavobacteriales	Flavobacteriaceae	Flavobacterium		0.12	0.17	0.15	0.09	0.09	0.05	0.04	0.02	0.03	0.07	0.06	0.18	0.22	0.23	0.10	0.26	0.14	0.05	0.08	0.16	0.20	0.10	0.15	0.17	0.16
AB38613	6	Bacteria	Bacteroidetes	[Saprospirae]	[Saprospirales]				0.07	0.03	0.02	0.04	0.05	0.27	0.31	0.07	0.10	0.06	0.05	0.07	0.07	0.11	0.04	0.10	0.03	0.06	0.03	0.04	0.03	0.04	0.03	0.06	0.06
AB18413	6	Bacteria	Planctomycetes	OM190	CL500-15				0.07	0.01	0.03	0.06	0.05	0.15	0.12	0.07	0.05	0.09	0.15	0.05	0.08	0.10	0.07	0.10	0.08	0.09	0.08	0.03	0.02	0.05	0.07	0.05	0.04
AB19623	5	Bacteria	Proteobacteria	Deltaproteobacteria	Myxococcales				1.26	0.89	0.96	2.12	2.25	2.11	2.33	1.01	0.84	1.66	1.82	1.16	1.02	1.40	1.29	1.32	1.27	1.01	1.03	0.98	1.01	0.86	0.77	0.57	0.61
AB17305	5	Bacteria	Acidobacteria	Acidobacteria-6	iii1-15				0.37	0.33	0.33	0.17	0.22	0.37	0.35	0.47	0.48	0.35	0.42	0.46	0.55	0.63	0.43	0.64	0.31	0.43	0.25	0.30	0.25	0.25	0.20	0.43	0.36
AB34910	5	Bacteria	Bacteroidetes	Cytophagia	Cytophagales				0.33	0.32	0.23	0.09	0.11	0.14	0.16	0.40	0.38	0.52	0.49	0.53	0.54	0.54	0.45	0.62	0.49	0.46	0.27	0.17	0.18	0.13	0.18	0.20	0.21
AB32798	5	Bacteria	Actinobacteria	Acidimicrobiia	Acidimicrobiales	Microthrixaceae			0.07	0.12	0.06	0.15	0.16	0.10	0.08	0.02	0.01	0.06	0.07	0.05	0.04	0.05	0.04	0.07	0.04	0.05	0.04	0.03	0.04	0.07	0.12	0.08	0.07
AB20008	4	Bacteria	Proteobacteria	Betaproteobacteria	Burkholderiales	Comamonadaceae			0.25	0.34	0.33	0.32	0.24	0.29	0.27	0.19	0.17	0.23	0.28	0.18	0.17	0.29	0.22	0.29	0.21	0.31	0.33	0.22	0.25	0.21	0.33	0.22	0.24
AB6541	4	Bacteria	Bacteroidetes	Flavobacteriia	Flavobacteriales	Flavobacteriaceae	Flavobacterium		0.20	0.22	0.23	0.24	0.28	0.27	0.20	0.22	0.20	0.14	0.13	0.17	0.18	0.15	0.24	0.14	0.21	0.14	0.19	0.22	0.24	0.12	0.13	0.20	0.20
AB5054	4	Bacteria	Bacteroidetes	[Saprospirae]	[Saprospirales]	Chitinophagaceae			0.16	0.16	0.17	0.34	0.39	0.23	0.28	0.17	0.17	0.08	0.05	0.13	0.13	0.07	0.10	0.13	0.08	0.13	0.11	0.14	0.12	0.06	0.11	0.26	0.19
AB34386	3	Bacteria	Bacteroidetes	Flavobacteriia	Flavobacteriales	Flavobacteriaceae	Flavobacterium		1.04	0.84	1.07	1.18	1.24	0.42	0.48	1.06	1.05	0.80	0.75	1.74	1.79	0.78	0.98	0.78	1.15	0.89	0.70	1.47	1.60	0.66	0.76	1.37	1.41
AB4600	3	Bacteria	Proteobacteria	Betaproteobacteria	Burkholderiales	Comamonadaceae	Leptothrix		0.63	0.27	0.24	0.83	0.89	0.61	0.50	0.18	0.17	0.68	0.84	1.08	1.17	0.28	0.78	0.35	0.76	0.72	0.90	0.08	0.06	0.77	0.81	1.11	1.12
AB22612	3	Bacteria	Proteobacteria	Deltaproteobacteria	Myxococcales				0.27	0.37	0.33	0.20	0.15	0.31	0.22	0.28	0.23	0.45	0.45	0.23	0.22	0.29	0.18	0.29	0.19	0.16	0.26	0.15	0.16	0.46	0.44	0.21	0.26
AB17145	3	Bacteria	Verrucomicrobia	Verrucomicrobiae	Verrucomicrobiales	Verrucomicrobiaceae	Luteolibacter		0.22	0.10	0.10	0.21	0.35	0.33	0.31	0.18	0.27	0.06	0.08	0.21	0.23	0.25	0.30	0.25	0.32	0.19	0.13	0.35	0.33	0.11	0.11	0.22	0.25
AB14641	3	Bacteria	Bacteroidetes	[Saprospirae]	[Saprospirales]	Saprospiraceae			0.12	0.25	0.23	0.10	0.12	0.11	0.13	0.17	0.22	0.11	0.09	0.14	0.10	0.12	0.09	0.08	0.06	0.06	0.10	0.10	0.12	0.06	0.07	0.14	0.13
AB37331	3	Bacteria	Bacteroidetes	Cytophagia	Cytophagales	Cytophagaceae	Flectobacillus		0.09	0.07	0.08	0.07	0.11	0.09	0.09	0.13	0.15	0.13	0.13	0.08	0.09	0.17	0.09	0.14	0.08	0.11	0.13	0.06	0.07	0.07	0.04	0.02	0.07
AB7381	3	Bacteria	Proteobacteria	Gammaproteobacteria	Aeromonadales	Aeromonadaceae			0.09	0.10	0.07	0.10	0.12	0.10	0.12	0.10	0.07	0.07	0.06	0.10	0.08	0.10	0.08	0.08	0.07	0.10	0.09	0.08	0.09	0.07	0.07	0.07	0.05
AB29568	2	Bacteria	Verrucomicrobia	[Pedosphaerae]	[Pedosphaerales]	auto67_4W			0.18	0.17	0.18	0.18	0.21	0.32	0.28	0.16	0.15	0.15	0.19	0.20	0.17	0.15	0.16	0.17	0.09	0.15	0.16	0.12	0.15	0.18	0.19	0.18	0.18
AB24846	2	Bacteria	Proteobacteria	Alphaproteobacteria	Rickettsiales				0.07	0.06	0.07	0.16	0.16	0.08	0.07	0.04	0.11	0.11	0.06	0.10	0.07	0.05	0.05	0.08	0.06	0.04	0.09	0.04	0.05	0.04	0.04	0.08	0.06
AB20720	1	Bacteria	Proteobacteria	Betaproteobacteria	Rhodocyclales	Rhodocyclaceae			0.54	1.24	1.06	0.17	0.16	0.33	0.38	1.10	1.22	0.40	0.43	0.22	0.24	0.99	0.19	1.23	0.22	0.32	0.40	0.59	0.59	0.44	0.47	0.29	0.32
AB24376	1	Bacteria	Bacteroidetes	[Saprospirae]	[Saprospirales]				0.35	0.10	0.10	0.47	0.46	0.42	0.43	0.60	0.54	0.23	0.19	0.34	0.32	0.27	0.25	0.33	0.26	0.22	0.19	0.63	0.67	0.23	0.34	0.42	0.28
AB10103	1	Bacteria	Acidobacteria	Solibacteres	Solibacterales	[Bryobacteraceae]			0.08	0.19	0.23	0.06	0.10	0.07	0.06	0.10	0.08	0.06	0.10	0.08	0.08	0.08	0.05	0.09	0.05	0.04	0.05	0.10	0.08	0.04	0.08	0.11	0.07
AB5173	1	Bacteria	Proteobacteria	Betaproteobacteria	Burkholderiales	Comamonadaceae			0.07	0.08	0.06	0.08	0.07	0.09	0.07	0.11	0.09	0.09	0.09	0.08	0.06	0.05	0.08	0.06	0.06	0.08	0.08	0.07	0.06	0.07	0.09	0.06	0.09
AB2202	1	Bacteria	Bacteroidetes	[Saprospirae]	[Saprospirales]	Chitinophagaceae			0.07	0.05	0.05	0.07	0.06	0.09	0.11	0.12	0.11	0.08	0.10	0.07	0.05	0.04	0.07	0.06	0.11	0.07	0.06	0.09	0.08	0.04	0.04	0.07	0.07
AB29074	1	Bacteria	Verrucomicrobia	Opitutae	Opitutales	Opitutaceae			0.07	0.04	0.08	0.05	0.05	0.13	0.09	0.07	0.06	0.02	0.04	0.08	0.08	0.09	0.07	0.09	0.08	0.05	0.05	0.05	0.06	0.07	0.06	0.07	0.06
AB14204	0	Bacteria	Proteobacteria	Epsilonproteobacteria	Campylobacterales	Campylobacteraceae	Arcobacter		0.29	0.20	0.18	0.35	0.43	0.30	0.33	0.32	0.27	0.30	0.32	0.27	0.29	0.27	0.28	0.29	0.29	0.24	0.23	0.26	0.26	0.34	0.31	0.24	0.28
AB33111	0	Bacteria	Proteobacteria	Betaproteobacteria	Burkholderiales				0.13	0.21	0.13	0.13	0.12	0.12	0.13	0.16	0.13	0.15	0.14	0.10	0.09	0.12	0.08	0.06	0.09	0.17	0.13	0.16	0.10	0.10	0.10	0.15	0.15
AB21747	0	Bacteria	Bacteroidetes	Sphingobacteriia	Sphingobacteriales	Sphingobacteriaceae			0.10	0.09	0.10	0.04	0.05	0.14	0.15	0.09	0.08	0.10	0.08	0.08	0.13	0.08	0.09	0.06	0.10	0.07	0.09	0.12	0.14	0.12	0.10	0.11	0.16

Supplemental Table S3. The correlation coefficients (Spearman's *rs*) and significance *p*-values for the interactions between abundant OTUs.

Node1	Node2	Spearman's *rs*	*p*-value
AB10086	AB10398	0.66435	0.00039924
AB10086	AB1126	0.79478	3.4995E-06
AB10086	AB11547	-0.84174	2.5355E-07
AB10086	AB14084	-0.96783	1.1355E-14
AB10086	AB15695	-0.69304	0.00017392
AB10086	AB17370	0.72522	0.000060868
AB10086	AB18149	0.88087	1.3489E-08
AB10086	AB20981	-0.9087	8.2631E-10
AB10086	AB21204	0.76174	0.000015298
AB10086	AB21536	0.65652	0.00049337
AB10086	AB23737	0.80435	2.1717E-06
AB10086	AB28110	0.75565	0.000019573
AB10086	AB29350	0.67913	0.00026315
AB10086	AB29432	-0.88174	1.2497E-08
AB10086	AB30657	-0.81652	1.1383E-06
AB10086	AB30755	-0.77826	7.5467E-06
AB10086	AB35151	0.7687	0.000011441
AB10086	AB37776	-0.92087	1.8141E-10
AB10086	AB38320	-0.79739	3.0802E-06
AB10086	AB38744	0.75217	0.000022462
AB10086	AB6558	0.64174	0.00072438
AB10086	AB7155	-0.79217	3.9689E-06
AB10086	AB8505	-0.66435	0.00039924
AB10103	AB27167	0.75217	0.000022462
AB10273	AB11344	0.66435	0.00039924
AB10273	AB14901	-0.67217	0.0003211
AB10398	AB14084	-0.66174	0.00042872
AB10398	AB15695	-0.81913	9.8508E-07
AB10398	AB18149	0.75652	0.000018904
AB10398	AB29432	-0.6487	0.00060612
AB10398	AB30657	-0.74435	0.000030377
AB10398	AB35151	0.69478	0.00016489
AB10458	AB10086	-0.63913	0.00077359
AB10458	AB1126	-0.66348	0.00040887
AB10458	AB14084	0.64174	0.00072438
AB10458	AB15695	0.75217	0.000022462
AB10458	AB16227	0.73391	0.0000447
AB10458	AB16638	-0.65623	0.00049723
AB10458	AB1696	0.74087	0.00003462
AB10458	AB17891	0.83304	4.3752E-07
AB10458	AB20981	0.69304	0.00017392
AB10458	AB21658	-0.65043	0.00057931
AB10458	AB23737	-0.77478	8.7997E-06
AB10458	AB29350	-0.80957	1.6557E-06
AB10458	AB30657	0.64435	0.00067789
AB10458	AB30755	0.74348	0.000031392
AB10458	AB36667	-0.6713	0.00032907
AB10458	AB37776	0.66	0.0004494
AB10458	AB38744	-0.7	0.00014019
AB10458	AB6639	-0.63217	0.00091914
AB10458	AB7155	0.63739	0.00080797
AB10458	AB836	0.65391	0.00052875
AB10458	AB9219	0.6713	0.00032907
AB11013	AB10273	0.63391	0.00088071
AB11013	AB1253	-0.64522	0.00066298
AB11013	AB14283	0.80348	2.2704E-06
AB11013	AB17447	0.73043	0.000050646
AB11013	AB19841	0.83565	3.7269E-07
AB11013	AB21377	0.66174	0.00042872
AB11013	AB21536	0.74609	0.000028432
AB11013	AB22647	-0.71391	0.000089426
AB11013	AB22668	0.66522	0.00038982
AB11013	AB22730	-0.65217	0.00055353
AB11013	AB22852	-0.65304	0.00054102
AB11013	AB23201	0.7	0.00014019
AB11013	AB2417	-0.6887	0.00019842
AB11013	AB28110	0.77739	7.8442E-06
AB11013	AB29320	-0.65739	0.00048204
AB11013	AB3071	0.67565	0.00029087
AB11013	AB34172	-0.87364	2.4903E-08
AB11013	AB35757	-0.75565	0.000019573
AB11013	AB36266	-0.76696	0.000012314
AB11013	AB38105	-0.79304	3.8066E-06
AB11013	AB643	0.7113	0.000097481
AB11013	AB8595	0.72696	0.000057276
AB1126	AB10398	0.71652	0.00008196
AB1126	AB11547	-0.65913	0.00046006
AB1126	AB12147	0.62957	0.00097949
AB1126	AB14084	-0.79565	3.3545E-06
AB1126	AB15695	-0.77478	8.7997E-06
AB1126	AB18149	0.75652	0.000018904
AB1126	AB29432	-0.80348	2.2704E-06
AB1126	AB30657	-0.92957	5.2532E-11
AB1126	AB30755	-0.87478	2.266E-08
AB1126	AB31424	0.79391	3.6502E-06
AB1126	AB35151	0.68957	0.0001933
AB1126	AB37776	-0.76261	0.00001476
AB1126	AB38320	-0.80609	1.9857E-06
AB11277	AB20919	-0.6713	0.00032907
AB11277	AB27638	-0.65043	0.00057931
AB11277	AB28115	0.64783	0.00061993
AB11277	AB29320	0.64	0.00075688
AB11277	AB34910	0.68522	0.00022014
AB11277	AB4160	-0.87217	2.8073E-08
AB11344	AB22204	-0.70957	0.0001032
AB11344	AB29888	-0.68348	0.00023175
AB11344	AB30418	-0.66783	0.00036268
AB11344	AB38635	-0.8913	5.1749E-09
AB11547	AB14084	0.87913	1.5688E-08
AB11547	AB18149	-0.74783	0.000026598
AB11547	AB19841	-0.68522	0.00022014
AB11547	AB2409	-0.65043	0.00057931
AB11547	AB29432	0.80348	2.2704E-06
AB11547	AB30657	0.67304	0.0003133
AB11547	AB30755	0.65739	0.00048204
AB11547	AB35151	-0.65217	0.00055353
AB11547	AB36000	-0.81565	1.1939E-06
AB11547	AB37776	0.80783	1.8141E-06
AB11547	AB6558	-0.79478	3.4995E-06
AB11702	AB22852	0.65565	0.00050493
AB11702	AB29320	0.64087	0.00074047
AB11702	AB34386	0.70261	0.0001291
AB11702	AB34896	-0.65043	0.00057931
AB11702	AB36266	0.64435	0.00067789
AB11702	AB4160	-0.71565	0.000084386
AB11702	AB5879	-0.70957	0.0001032
AB11702	AB8595	-0.67739	0.00027671
AB12147	AB36667	0.85217	1.2602E-07
AB1253	AB11344	-0.63913	0.00077359
AB1253	AB19841	-0.70261	0.0001291
AB1253	AB22204	0.67739	0.00027671
AB1253	AB22852	0.76696	0.000012314
AB1253	AB25200	-0.71217	0.000094728
AB1253	AB29320	0.80087	2.5907E-06
AB1253	AB34172	0.84096	2.6659E-07
AB1253	AB38635	0.6913	0.00018339
AB1253	AB4160	-0.63304	0.00089975
AB1253	AB5129	-0.63739	0.00080797
AB1253	AB6558	-0.6513	0.00056629
AB12784	AB10273	-0.82087	8.9343E-07
AB12784	AB11344	-0.76522	0.000013246
AB12784	AB1253	0.7687	0.000011441
AB12784	AB12943	-0.65652	0.00049337
AB12784	AB13425	0.6313	0.00093889
AB12784	AB14160	-0.65739	0.00048204
AB12784	AB14616	-0.88783	7.1964E-09
AB12784	AB14788	-0.70087	0.00013641
AB12784	AB14975	-0.79826	2.9507E-06
AB12784	AB16227	-0.6887	0.00019842
AB12784	AB16898	-0.78344	5.9723E-06
AB12784	AB17447	-0.78783	4.8761E-06
AB12784	AB19841	-0.63739	0.00080797
AB12784	AB21377	-0.69478	0.00016489
AB12784	AB21536	-0.64696	0.000634
AB12784	AB22204	0.75739	0.000018255
AB12784	AB22730	0.65913	0.00046006
AB12784	AB22852	0.64348	0.00069309
AB12784	AB2417	0.78	6.9817E-06
AB12784	AB26668	0.78783	4.8761E-06
AB12784	AB28115	0.6687	0.00035401
AB12784	AB28385	-0.74609	0.000028432
AB12784	AB29320	0.78435	5.7298E-06
AB12784	AB3071	-0.64696	0.000634
AB12784	AB33467	0.80787	1.8101E-06
AB12784	AB34172	0.75817	0.00001769
AB12784	AB34896	-0.83217	4.6126E-07
AB12784	AB35757	0.8087	1.7333E-06
AB12784	AB35792	0.79478	3.4995E-06
AB12784	AB36266	0.72261	0.000066627
AB12784	AB38105	0.76435	0.000013735
AB12784	AB38191	-0.68609	0.00021453
AB12784	AB38635	0.72609	0.000059048
AB12784	AB501	0.70609	0.00011552
AB12784	AB5129	-0.72522	0.000060868
AB12784	AB6398	-0.64174	0.00072438
AB12784	AB643	-0.69304	0.00017392
AB12784	AB8595	-0.70957	0.0001032
AB12784	AB9593	0.76696	0.000012314
AB12784	AB9798	-0.66609	0.00038058
AB12943	AB11702	-0.71739	0.000079596
AB12943	AB1253	-0.80174	2.4797E-06
AB12943	AB14160	0.68174	0.0002439
AB12943	AB14616	0.67478	0.00029819
AB12943	AB16898	0.71896	0.000075493
AB12943	AB17447	0.76	0.000016426
AB12943	AB19841	0.63043	0.00095901
AB12943	AB22204	-0.74522	0.00002939
AB12943	AB22730	-0.7	0.00014019
AB12943	AB22852	-0.80348	2.2704E-06
AB12943	AB2417	-0.66522	0.00038982
AB12943	AB25200	0.74435	0.000030377
AB12943	AB27638	0.64696	0.000634
AB12943	AB28115	-0.7713	0.000010233
AB12943	AB28385	0.74609	0.000028432
AB12943	AB29320	-0.82609	6.6234E-07
AB12943	AB29749	-0.66609	0.00038058
AB12943	AB29888	-0.77478	8.7997E-06
AB12943	AB30418	-0.63478	0.00086202
AB12943	AB34172	-0.72723	0.000056719
AB12943	AB34896	0.82261	8.0947E-07
AB12943	AB35792	-0.65478	0.00051672
AB12943	AB36266	-0.68522	0.00022014
AB12943	AB38191	0.85565	9.8643E-08
AB12943	AB38635	-0.69043	0.00018828
AB12943	AB4160	0.78174	6.4545E-06
AB12943	AB5129	0.74696	0.000027502
AB12943	AB5879	0.72783	0.000055551
AB12943	AB643	0.7887	4.6812E-06
AB12943	AB8595	0.66783	0.00036268
AB12943	AB9593	-0.65913	0.00046006
AB13162	AB11344	0.67304	0.0003133
AB13162	AB14616	0.67565	0.00029087
AB13162	AB14788	0.78	6.9817E-06
AB13162	AB16898	0.63007	0.00096763
AB13162	AB18208	-0.73304	0.000046126
AB13162	AB19841	0.67391	0.00030566
AB13162	AB21536	0.64	0.00075688
AB13162	AB22204	-0.77304	9.4926E-06
AB13162	AB22852	-0.65739	0.00048204
AB13162	AB2417	-0.63913	0.00077359
AB13162	AB28110	0.65391	0.00052875
AB13162	AB29320	-0.67913	0.00026315
AB13162	AB29749	-0.73565	0.000041965
AB13162	AB29888	-0.69826	0.00014804
AB13162	AB34896	0.63652	0.00082565
AB13162	AB35757	-0.64	0.00075688
AB13162	AB36266	-0.69217	0.0001786
AB13162	AB38635	-0.75826	0.000017626
AB13162	AB5129	0.70435	0.00012214
AB13162	AB643	0.65217	0.00055353
AB13425	AB1949	-0.67565	0.00029087
AB13425	AB22852	0.68435	0.00022588
AB13425	AB24131	-0.67043	0.00033721
AB13425	AB38320	0.72522	0.000060868
AB13660	AB1253	0.65304	0.00054102
AB13660	AB13425	0.79304	3.8066E-06
AB13660	AB14160	-0.63739	0.00080797
AB13660	AB1949	-0.70435	0.00012214
AB13660	AB20152	-0.70435	0.00012214
AB13660	AB22852	0.66087	0.00043896
AB13660	AB23797	-0.68522	0.00022014
AB13660	AB2417	0.7913	4.1373E-06
AB13660	AB24262	0.66348	0.00040887
AB13660	AB34172	0.70283	0.00012818
AB13660	AB36266	0.69652	0.00015626
AB13660	AB38320	0.7313	0.000049098
AB13660	AB6398	-0.67913	0.00026315
AB14084	AB18149	-0.91478	3.9823E-10
AB14084	AB29432	0.92522	9.9458E-11
AB14084	AB30657	0.83652	3.5307E-07
AB14084	AB35151	-0.81217	1.4413E-06
AB14084	AB36000	-0.66522	0.00038982
AB14084	AB37776	0.94609	3.0004E-12
AB14084	AB38320	0.76435	0.000013735
AB14084	AB6558	-0.65391	0.00052875
AB14160	AB1253	-0.74348	0.000031392
AB14160	AB22204	-0.73826	0.000038137
AB14160	AB22852	-0.7487	0.000025721
AB14160	AB29320	-0.76696	0.000012314
AB14160	AB34172	-0.73769	0.000038944
AB14160	AB35792	-0.67304	0.0003133
AB14160	AB36266	-0.64609	0.00064835

AB14283	AB19841	0.80435	2.1717E-06
AB14616	AB10273	0.84609	1.9066E-07
AB14616	AB11013	0.70174	0.00013271
AB14616	AB11344	0.75043	0.000024043
AB14616	AB1253	-0.75391	0.000020974
AB14616	AB14788	0.67217	0.0003211
AB14616	AB14901	-0.63652	0.00082565
AB14616	AB17447	0.88609	8.4523E-09
AB14616	AB19841	0.84087	2.6815E-07
AB14616	AB21377	0.85826	8.1747E-08
AB14616	AB21536	0.79217	3.9689E-06
AB14616	AB22204	-0.68261	0.00023776
AB14616	AB22647	-0.73304	0.000046126
AB14616	AB22730	-0.76	0.000016426
AB14616	AB22852	-0.64957	0.00059259
AB14616	AB23201	0.6313	0.00093889
AB14616	AB2417	-0.73391	0.0000447
AB14616	AB25200	0.77304	9.4926E-06
AB14616	AB28110	0.79565	3.3545E-06
AB14616	AB28115	-0.79652	3.2147E-06
AB14616	AB29320	-0.79217	3.9689E-06
AB14616	AB3071	0.66957	0.00034552
AB14616	AB34172	-0.80349	2.2693E-06
AB14616	AB34896	0.83652	3.5307E-07
AB14616	AB35757	-0.93826	1.2845E-11
AB14616	AB35792	-0.78174	6.4545E-06
AB14616	AB36266	-0.78609	5.2877E-06
AB14616	AB38105	-0.89739	2.8267E-09
AB14616	AB38635	-0.78174	6.4545E-06
AB14616	AB5129	0.80174	2.4797E-06
AB14616	AB5879	0.79043	0.000004312
AB14616	AB6398	0.64087	0.00074047
AB14616	AB643	0.69043	0.00018828
AB14616	AB6558	0.69826	0.00014804
AB14616	AB8595	0.78957	4.4932E-06
AB14616	AB9798	0.64261	0.00070859
AB14641	AB1491	0.79739	3.0802E-06
AB14641	AB27167	0.64435	0.00067789
AB14641	AB31424	-0.70522	0.00011879
AB14788	AB11344	0.67478	0.00029819
AB14788	AB1696	0.66	0.0004494
AB14788	AB22204	-0.8287	5.6818E-07
AB14788	AB29320	-0.63304	0.00089975
AB14788	AB3071	0.7487	0.000025721
AB14788	AB31803	0.7887	4.6812E-06
AB14788	AB34172	-0.67451	0.0003005
AB14788	AB38635	-0.65478	0.00051672
AB14788	AB5129	0.75304	0.000021707
AB1491	AB12147	-0.66783	0.00036268
AB14975	AB11013	0.66087	0.00043896
AB14975	AB11344	0.79478	3.4995E-06
AB14975	AB1253	-0.74087	0.00003462
AB14975	AB12943	0.82522	6.9669E-07
AB14975	AB14160	0.75043	0.000024043
AB14975	AB14616	0.78609	5.2877E-06
AB14975	AB14788	0.65652	0.00049337
AB14975	AB16227	0.73391	0.0000447
AB14975	AB16898	0.76035	0.000016193
AB14975	AB17447	0.85304	1.186E-07
AB14975	AB19878	-0.72261	0.000066627
AB14975	AB22204	-0.74435	0.000030377
AB14975	AB22730	-0.76174	0.000015298
AB14975	AB22852	-0.68609	0.00021453
AB14975	AB2417	-0.6887	0.00019842
AB14975	AB25200	0.73478	0.000043313
AB14975	AB26668	-0.71652	0.00008196
AB14975	AB28115	-0.78957	4.4932E-06
AB14975	AB28385	0.83478	3.9327E-07
AB14975	AB29320	-0.88783	7.1964E-09
AB14975	AB29888	-0.74957	0.00002487
AB14975	AB30418	-0.68087	0.00025017
AB14975	AB3071	0.71826	0.000077292
AB14975	AB31803	0.74957	0.00002487
AB14975	AB33467	-0.89084	5.4122E-09
AB14975	AB34172	-0.79652	3.2156E-06
AB14975	AB34896	0.78435	5.7298E-06
AB14975	AB35757	-0.72087	0.000070727
AB14975	AB35792	-0.83478	3.9327E-07
AB14975	AB36266	-0.63304	0.00089975
AB14975	AB38105	-0.7487	0.000025721
AB14975	AB38191	0.80348	2.2704E-06
AB14975	AB38635	-0.77652	8.1519E-06
AB14975	AB4160	0.6913	0.00018339
AB14975	AB501	-0.81913	9.8508E-07
AB14975	AB5129	0.85478	1.0493E-07
AB14975	AB643	0.78087	6.7135E-06
AB14975	AB6541	0.65304	0.00054102
AB14975	AB836	0.68609	0.00021453
AB14975	AB8595	0.63913	0.00077359
AB14975	AB9593	-0.86348	5.5507E-08
AB15099	AB14788	0.75826	0.000017626
AB15099	AB15541	0.68609	0.00021453
AB15099	AB16227	0.78957	4.4932E-06
AB15099	AB1696	0.75304	0.000021707
AB15099	AB18669	0.72174	0.00006865
AB15099	AB29749	-0.6713	0.00032907
AB15099	AB31528	0.68783	0.00020367
AB15099	AB35792	-0.66261	0.00041869
AB15099	AB36667	-0.72348	0.000064657
AB15099	AB6639	-0.8113	1.5098E-06
AB15099	AB7164	0.75478	0.000020263
AB15099	AB8930	-0.78957	4.4932E-06
AB15099	AB9798	0.75043	0.000024043
AB15257	AB17305	0.63391	0.00088071
AB15257	AB18208	0.65913	0.00046006
AB15257	AB1949	0.68348	0.00023175
AB15257	AB30418	0.67826	0.00026986
AB15257	AB34451	0.75652	0.000018904
AB15541	AB1696	0.76087	0.000015853
AB15541	AB25552	0.71391	0.000089426
AB15541	AB35792	-0.64783	0.00061993
AB15541	AB36667	-0.66261	0.00041869
AB15541	AB6639	-0.68087	0.00025017
AB15541	AB8930	-0.65478	0.00051672
AB15541	AB9798	0.91304	4.9324E-10
AB15695	AB14084	0.72087	0.000070727
AB15695	AB18149	-0.71739	0.000079596
AB15695	AB29432	0.79304	3.8066E-06
AB15695	AB30657	0.76348	0.000014239
AB15695	AB35151	-0.76087	0.000015853
AB15695	AB37776	0.69739	0.0001521
AB16076	AB20919	-0.64	0.00075688
AB16076	AB20940	-0.70957	0.0001032
AB16076	AB34910	0.78609	5.2877E-06
AB16227	AB12147	-0.76435	0.000013735
AB16227	AB14160	0.65826	0.00047094
AB16227	AB14788	0.77043	0.000010623
AB16227	AB15695	0.66522	0.00038982
AB16227	AB1696	0.91043	6.7432E-10
AB16227	AB22204	-0.76	0.000016426
AB16227	AB3071	0.80261	0.000002373
AB16227	AB31424	-0.69217	0.0001786
AB16227	AB31803	0.8	2.7061E-06
AB16227	AB35792	-0.88957	6.1109E-09
AB16227	AB36667	-0.87478	2.266E-08
AB16227	AB5129	0.73043	0.000050646
AB16227	AB6639	-0.77565	8.4703E-06
AB16227	AB8930	-0.82696	6.2951E-07
AB16227	AB9798	0.72	0.000072859
AB16638	AB1696	-0.6297	0.00097624
AB16638	AB17447	-0.64666	0.00063883
AB16638	AB22730	0.68319	0.00023372
AB16638	AB24002	0.64579	0.00065327
AB16638	AB24131	0.70015	0.00013952
AB16638	AB31528	-0.68232	0.00023977
AB16638	AB31803	-0.79713	3.1201E-06
AB16638	AB5129	-0.73016	0.000051146
AB16638	AB6639	0.69232	0.00017778
AB16638	AB836	-0.80148	2.5125E-06
AB16638	AB8930	0.72059	0.000071403
AB16799	AB12943	0.7	0.00014019
AB16799	AB14975	0.67043	0.00033721
AB16799	AB19878	-0.63652	0.00082565
AB16799	AB21646	0.67754	0.00027554
AB16799	AB21658	-0.71739	0.000079596
AB16799	AB22852	-0.69304	0.00017392
AB16799	AB25200	0.77217	9.8569E-06
AB16799	AB29320	-0.65739	0.00048204
AB16799	AB34896	0.69304	0.00017392
AB16799	AB38191	0.72609	0.000059048
AB16799	AB4160	0.63913	0.00077359
AB16799	AB6398	0.63913	0.00077359
AB16898	AB10273	0.73813	0.000038324
AB16898	AB11013	0.8	2.7059E-06
AB16898	AB11344	0.69194	0.00017987
AB16898	AB11702	-0.63442	0.00086968
AB16898	AB12147	-0.64314	0.00069911
AB16898	AB1253	-0.74074	0.000034785
AB16898	AB14283	0.71852	0.000076617
AB16898	AB14616	0.88541	8.9962E-09
AB16898	AB14788	0.64619	0.00064666
AB16898	AB14901	-0.66885	0.00035252
AB16898	AB17447	0.817	0.000001109
AB16898	AB19841	0.85621	9.478E-08
AB16898	AB20940	0.66667	0.00037452
AB16898	AB21377	0.76863	0.000011473
AB16898	AB21536	0.72593	0.000059378
AB16898	AB22204	-0.74205	0.000033125
AB16898	AB22647	-0.78257	6.2146E-06
AB16898	AB22668	0.70458	0.00012125
AB16898	AB22730	-0.70371	0.00012468
AB16898	AB22852	-0.70153	0.0001336
AB16898	AB23201	0.70937	0.00010386
AB16898	AB2417	-0.63007	0.00096763
AB16898	AB25200	0.71068	0.00009951
AB16898	AB28110	0.73856	0.000037713
AB16898	AB28115	-0.74161	0.000033671
AB16898	AB29320	-0.85273	1.2127E-07
AB16898	AB29888	-0.64706	0.00063231
AB16898	AB3071	0.71068	0.00009951
AB16898	AB34172	-0.80742	1.8526E-06
AB16898	AB34896	0.8183	1.0316E-06
AB16898	AB35757	-0.85142	1.328E-07
AB16898	AB35792	-0.78824	4.7831E-06
AB16898	AB36266	-0.79652	3.2156E-06
AB16898	AB38105	-0.86536	4.808E-08
AB16898	AB38191	0.78475	5.6244E-06
AB16898	AB38635	-0.75599	0.000019308
AB16898	AB5129	0.72593	0.000059378
AB16898	AB5879	0.77124	0.00001026
AB16898	AB643	0.68976	0.00019215
AB16898	AB6558	0.64009	0.00075519
AB16898	AB8595	0.8963	3.1595E-09
AB1696	AB12147	-0.80609	1.9857E-06
AB1696	AB1491	0.76609	0.000012772
AB1696	AB3071	0.77391	9.1403E-06
AB1696	AB31424	-0.7913	4.1373E-06
AB1696	AB31803	0.74435	0.000030377
AB1696	AB36667	-0.8913	5.1749E-09
AB1696	AB5129	0.63913	0.00077359
AB17145	AB22612	-0.72522	0.000060868
AB17145	AB24376	0.64783	0.00061993
AB17305	AB2409	-0.66174	0.00042872
AB17305	AB35208	-0.67913	0.00026315
AB17370	AB10273	0.72	0.000072859
AB17370	AB11013	0.77391	9.1403E-06
AB17370	AB11547	-0.74609	0.000028432
AB17370	AB1253	-0.70261	0.0001291
AB17370	AB12784	-0.7287	0.000053871
AB17370	AB13425	-0.68522	0.00022014
AB17370	AB13660	-0.64435	0.00067789
AB17370	AB14084	-0.74348	0.000031392
AB17370	AB14283	0.71304	0.000092043
AB17370	AB14616	0.84783	1.697E-07
AB17370	AB16898	0.7695	0.000011055
AB17370	AB17447	0.69304	0.00017392
AB17370	AB18149	0.67217	0.0003211
AB17370	AB19841	0.89043	5.6254E-09
AB17370	AB21377	0.74957	0.00002487
AB17370	AB21536	0.87565	2.1077E-08
AB17370	AB21646	0.78408	5.7998E-06
AB17370	AB22647	-0.7713	0.000010233
AB17370	AB22852	-0.71652	0.00008196
AB17370	AB23201	0.65304	0.00054102
AB17370	AB2417	-0.82957	5.3956E-07
AB17370	AB24262	-0.65739	0.00048204
AB17370	AB25200	0.78348	5.9629E-06
AB17370	AB2526	0.73478	0.000043313
AB17370	AB26668	-0.91391	4.4345E-10
AB17370	AB28110	0.93652	1.7294E-11
AB17370	AB28115	-0.76522	0.000013246
AB17370	AB28385	0.82261	8.0947E-07
AB17370	AB29320	-0.67913	0.00026315
AB17370	AB29432	-0.69826	0.00014804
AB17370	AB33467	-0.74324	0.000031677
AB17370	AB34172	-0.85447	1.0729E-07
AB17370	AB34896	0.67826	0.00026986
AB17370	AB35757	-0.90609	1.1125E-09
AB17370	AB36000	0.72435	0.000062737
AB17370	AB36266	-0.85478	1.0493E-07
AB17370	AB37776	-0.73652	0.000040653
AB17370	AB38105	-0.88348	1.0707E-08
AB17370	AB38191	0.63652	0.00082565
AB17370	AB38635	-0.67739	0.00027671

AB17370	AB5879	0.68435	0.00022588
AB17370	AB6398	0.75391	0.000020974
AB17370	AB643	0.75217	0.000022462
AB17370	AB6558	0.87304	2.6152E-08
AB17447	AB10273	0.66087	0.00043896
AB17447	AB11344	0.7	0.00014019
AB17447	AB1253	-0.74522	0.00002939
AB17447	AB14788	0.71652	0.00008196
AB17447	AB16227	0.65304	0.00054102
AB17447	AB19841	0.75739	0.000018255
AB17447	AB21377	0.81304	1.3755E-06
AB17447	AB21536	0.67391	0.00030566
AB17447	AB22204	-0.67826	0.00026986
AB17447	AB22647	-0.68261	0.00023776
AB17447	AB22730	-0.90609	1.1125E-09
AB17447	AB2417	-0.63652	0.00082565
AB17447	AB25200	0.66696	0.00037154
AB17447	AB29320	-0.78261	6.2044E-06
AB17447	AB29888	-0.64783	0.00061993
AB17447	AB3071	0.78957	4.4932E-06
AB17447	AB31803	0.7887	4.6812E-06
AB17447	AB34172	-0.77342	9.3377E-06
AB17447	AB35757	-0.8313	4.8615E-07
AB17447	AB35792	-0.73565	0.000041965
AB17447	AB36266	-0.71391	0.000089426
AB17447	AB38635	-0.69652	0.00015626
AB17447	AB5129	0.93565	2.0004E-11
AB1746	AB11702	-0.70696	0.00011232
AB1746	AB1253	-0.69913	0.00014407
AB1746	AB12943	0.79652	3.2147E-06
AB1746	AB16799	0.66957	0.00034552
AB1746	AB17370	0.63391	0.00088071
AB1746	AB19841	0.69478	0.00016489
AB1746	AB21646	0.65797	0.00047464
AB1746	AB21903	0.72435	0.000062737
AB1746	AB22218	-0.64783	0.00061993
AB1746	AB22852	-0.91565	3.5722E-10
AB1746	AB23287	-0.6883	0.00020079
AB1746	AB23797	0.73478	0.000043313
AB1746	AB2417	-0.7	0.00014019
AB1746	AB25200	0.78261	6.2044E-06
AB1746	AB27167	-0.64348	0.00069309
AB1746	AB2747	0.71478	0.000086874
AB1746	AB28115	-0.64957	0.00059259
AB1746	AB28385	0.6713	0.00032907
AB1746	AB29320	-0.72	0.000072859
AB1746	AB33467	-0.71966	0.000073714
AB1746	AB34172	-0.67582	0.00028949
AB1746	AB34386	-0.67652	0.00028371
AB1746	AB34896	0.6887	0.00019842
AB1746	AB36266	-0.79826	2.9507E-06
AB1746	AB37554	-0.77826	7.5467E-06
AB1746	AB38191	0.74174	0.000033513
AB1746	AB4160	0.63739	0.00080797
AB1746	AB5879	0.68522	0.00022014
AB1746	AB6398	0.66087	0.00043896
AB1746	AB643	0.7887	4.6812E-06
AB1746	AB6558	0.76087	0.000015853
AB175	AB10398	0.68174	0.0002439
AB175	AB1126	0.80957	1.6557E-06
AB175	AB12147	0.70696	0.00011232
AB175	AB16076	0.64087	0.00074047
AB175	AB20981	-0.63913	0.00077359
AB175	AB21204	0.64696	0.000634
AB175	AB30657	-0.78783	4.8761E-06
AB175	AB30755	-0.76957	0.000011025
AB175	AB31424	0.68783	0.00020367
AB175	AB38744	0.70261	0.0001291
AB175	AB7155	-0.68	0.00025659
AB175	AB8505	-0.72	0.000072859
AB17891	AB1126	-0.63391	0.00088071
AB17891	AB12147	-0.7487	0.000025721
AB17891	AB1491	0.68522	0.00022014
AB17891	AB15099	0.63478	0.00086202
AB17891	AB15695	0.68522	0.00022014
AB17891	AB16227	0.81304	1.3755E-06
AB17891	AB16638	-0.72581	0.000059623
AB17891	AB1696	0.88261	1.1571E-08
AB17891	AB26009	-0.6313	0.00093889
AB17891	AB27392	0.6313	0.00093889
AB17891	AB29350	-0.72783	0.000055551
AB17891	AB3071	0.78261	6.2044E-06
AB17891	AB31424	-0.78087	6.7135E-06
AB17891	AB31803	0.76348	0.000014239
AB17891	AB35792	-0.6687	0.00035401
AB17891	AB36667	-0.79565	3.3545E-06
AB17891	AB38744	-0.66435	0.00039924
AB17891	AB6639	-0.76783	0.00001187
AB17891	AB836	0.75826	0.000017626
AB17891	AB8930	-0.75478	0.000020263
AB17891	AB9219	0.71826	0.000077292
AB18149	AB37776	-0.88261	1.1571E-08
AB18149	AB38320	-0.75913	0.000017017
AB18208	AB22647	0.63391	0.00088071
AB18208	AB2409	-0.67478	0.00029819
AB18208	AB29320	0.69652	0.00015626
AB18208	AB29888	0.84174	2.5355E-07
AB18208	AB30418	0.82435	7.3262E-07
AB18208	AB38635	0.80348	2.2704E-06
AB18208	AB5129	-0.74087	0.00003462
AB18413	AB19623	0.65043	0.00057931
AB18413	AB27167	-0.72609	0.000059048
AB18413	AB29749	-0.64	0.00075688
AB18413	AB35155	-0.64957	0.00059259
AB18413	AB35801	0.73652	0.000040653
AB18413	AB4035	0.74261	0.000032437
AB18669	AB14901	-0.64696	0.000634
AB18669	AB1696	0.62957	0.00097949
AB18669	AB24131	-0.71043	0.0001003
AB18669	AB26009	-0.67043	0.00033721
AB18669	AB26225	-0.80087	2.5907E-06
AB18669	AB6895	-0.68087	0.00025017
AB18669	AB7164	0.63826	0.00079062
AB18669	AB8930	-0.7	0.00014019
AB19228	AB12943	0.63043	0.00095901
AB19228	AB13162	0.68957	0.0001933
AB19228	AB18208	-0.8287	5.6818E-07
AB19228	AB29888	-0.82609	6.6234E-07
AB19228	AB30418	-0.72522	0.000060868
AB19228	AB38635	-0.70174	0.00013271
AB19228	AB5129	0.6887	0.00019842
AB19439	AB10273	0.79391	3.6502E-06
AB19439	AB11013	0.6687	0.00035401
AB19439	AB11344	0.73304	0.000046126
AB19439	AB1253	-0.78957	4.4932E-06
AB19439	AB12784	-0.91478	3.9823E-10
AB19439	AB13162	0.63391	0.00088071
AB19439	AB13425	-0.72783	0.000055551
AB19439	AB13660	-0.6913	0.00018339
AB19439	AB14160	0.68696	0.00020904
AB19439	AB14616	0.84522	2.0198E-07
AB19439	AB14788	0.7087	0.00010616
AB19439	AB14975	0.72	0.000072859
AB19439	AB16898	0.74336	0.000031536
AB19439	AB17370	0.79478	3.4995E-06
AB19439	AB17447	0.72435	0.000062737
AB19439	AB19841	0.67217	0.0003211
AB19439	AB21377	0.64783	0.00061993
AB19439	AB21536	0.72783	0.000055551
AB19439	AB21646	0.70624	0.00011495
AB19439	AB22204	-0.75652	0.000018904
AB19439	AB22852	-0.71043	0.0001003
AB19439	AB23287	-0.78118	6.6188E-06
AB19439	AB2417	-0.8087	1.7333E-06
AB19439	AB24263	-0.72522	0.000060868
AB19439	AB25200	0.63826	0.00079062
AB19439	AB2526	0.76348	0.000014239
AB19439	AB26668	-0.79217	3.9689E-06
AB19439	AB28110	0.69826	0.00014804
AB19439	AB28115	-0.67478	0.00029819
AB19439	AB28385	0.72522	0.000060868
AB19439	AB28610	-0.74957	0.00002487
AB19439	AB29320	-0.77217	9.8569E-06
AB19439	AB33467	-0.78341	5.9806E-06
AB19439	AB33890	0.73478	0.000043313
AB19439	AB34172	-0.79172	4.0554E-06
AB19439	AB34896	0.77043	0.000010623
AB19439	AB35757	-0.8113	1.5098E-06
AB19439	AB35792	-0.71043	0.0001003
AB19439	AB36266	-0.8	2.7061E-06
AB19439	AB38105	-0.74087	0.00003462
AB19439	AB38191	0.66522	0.00038982
AB19439	AB38635	-0.72783	0.000055551
AB19439	AB501	-0.63739	0.00080797
AB19439	AB5129	0.65478	0.00051672
AB19439	AB6398	0.68783	0.00020367
AB19439	AB643	0.67826	0.00026986
AB19439	AB9593	-0.63913	0.00077359
AB19439	AB9798	0.64609	0.00064835
AB1945	AB26225	0.80087	2.5907E-06
AB1945	AB6558	0.68	0.00025659
AB1949	AB38320	-0.6687	0.00035401
AB19623	AB22204	-0.69217	0.0001786
AB19740	AB11013	0.68174	0.0002439
AB19740	AB11344	0.63217	0.00091914
AB19740	AB12943	0.64435	0.00067789
AB19740	AB13162	0.78348	5.9629E-06
AB19740	AB14283	0.68435	0.00022588
AB19740	AB14616	0.71652	0.00008196
AB19740	AB14788	0.64174	0.00072438
AB19740	AB14975	0.68348	0.00023175
AB19740	AB16898	0.66144	0.00043222
AB19740	AB17370	0.71478	0.000086874
AB19740	AB17447	0.76696	0.000012314
AB19740	AB18208	-0.86696	4.2501E-08
AB19740	AB19228	0.65652	0.00049337
AB19740	AB19841	0.79217	3.9689E-06
AB19740	AB2057	-0.75861	0.000017381
AB19740	AB21377	0.78435	5.7298E-06
AB19740	AB21536	0.75826	0.000017626
AB19740	AB21646	0.72059	0.000071403
AB19740	AB21903	0.76609	0.000012772
AB19740	AB22218	-0.64261	0.00070859
AB19740	AB22647	-0.72087	0.000070727
AB19740	AB22730	-0.78783	4.8761E-06
AB19740	AB23287	-0.79959	2.7627E-06
AB19740	AB2409	0.6313	0.00093889
AB19740	AB24262	-0.63043	0.00095901
AB19740	AB25200	0.65565	0.00050493
AB19740	AB2526	0.73565	0.000041965
AB19740	AB26668	-0.75913	0.000017017
AB19740	AB28110	0.76609	0.000012772
AB19740	AB28115	-0.84261	2.3966E-07
AB19740	AB28385	0.86783	3.9711E-08
AB19740	AB28610	-0.75478	0.000020263
AB19740	AB28820	-0.71304	0.000092043
AB19740	AB29320	-0.72609	0.000059048
AB19740	AB29888	-0.78696	5.0782E-06
AB19740	AB30418	-0.78957	4.4932E-06
AB19740	AB31803	0.63391	0.00088071
AB19740	AB33467	-0.74848	0.000025938
AB19740	AB34172	-0.71155	0.000096701
AB19740	AB35757	-0.74957	0.00002487
AB19740	AB36266	-0.67826	0.00026986
AB19740	AB37554	-0.72957	0.000052237
AB19740	AB38105	-0.77913	7.2594E-06
AB19740	AB38635	-0.7687	0.000011441
AB19740	AB38882	-0.70696	0.00011232
AB19740	AB5129	0.80261	0.000002373
AB19740	AB643	0.77217	9.8569E-06
AB19740	AB6490	-0.7687	0.000011441
AB19740	AB6558	0.75565	0.000019573
AB19841	AB10273	0.68	0.00025659
AB19841	AB11344	0.64	0.00075688
AB19841	AB22852	-0.70609	0.00011552
AB19841	AB36000	0.70609	0.00011552
AB19841	AB38635	-0.7313	0.000049098
AB19841	AB5129	0.64609	0.00064835
AB19841	AB6558	0.81913	9.8508E-07
AB19878	AB10398	0.7087	0.00010616
AB19878	AB11277	0.74	0.000035759
AB19878	AB12943	-0.75478	0.000020263
AB19878	AB15695	-0.71043	0.0001003
AB19878	AB175	0.64696	0.000634
AB19878	AB29320	0.65565	0.00050493
AB19878	AB29350	0.66696	0.00037154
AB19878	AB29888	0.74609	0.000028432
AB19878	AB30418	0.65391	0.00052875
AB19878	AB30657	-0.70957	0.0001032
AB19878	AB30755	-0.75913	0.000017017
AB19878	AB31928	0.68174	0.0002439
AB19878	AB35208	-0.64696	0.000634
AB19878	AB38744	0.77391	9.1403E-06
AB19878	AB4160	-0.81739	0.000001085
AB19878	AB501	0.71304	0.000092043
AB19878	AB7155	-0.7687	0.000011441
AB19878	AB9593	0.7113	0.000097481
AB20008	AB30418	-0.63478	0.00086202
AB20008	AB35208	0.78609	5.2877E-06
AB20152	AB14160	0.73826	0.000038137
AB20152	AB33847	-0.72174	0.00006865
AB20152	AB35801	0.65565	0.00050493
AB20152	AB4035	0.68609	0.00021453
AB20432	AB12784	-0.64261	0.00070859

AB20432	AB14616	0.69043	0.00018828
AB20432	AB16898	0.64314	0.00069911
AB20432	AB17447	0.74609	0.000028432
AB20432	AB20940	0.74609	0.000028432
AB20432	AB21377	0.76609	0.000012772
AB20432	AB22730	-0.78435	5.7298E-06
AB20432	AB2526	0.68522	0.00022014
AB20432	AB28820	-0.67391	0.00030566
AB20432	AB3071	0.64696	0.000634
AB20432	AB31456	-0.67565	0.00029087
AB20432	AB36667	-0.66696	0.00037154
AB20432	AB4262	-0.63145	0.00093562
AB20432	AB5129	0.66522	0.00038982
AB20432	AB836	0.64783	0.00061993
AB20432	AB8595	0.82957	5.3956E-07
AB20432	AB8930	-0.78174	6.4545E-06
AB2057	AB10273	-0.66667	0.00037452
AB2057	AB11344	-0.78344	5.9723E-06
AB2057	AB1253	0.65752	0.00048041
AB2057	AB12784	0.78824	4.7831E-06
AB2057	AB13162	-0.63747	0.0008063
AB2057	AB14616	-0.81917	9.8268E-07
AB2057	AB14975	-0.76689	0.00001235
AB2057	AB16898	-0.67598	0.00028813
AB2057	AB17370	-0.80305	2.3202E-06
AB2057	AB17447	-0.7207	0.000071141
AB2057	AB18208	0.65665	0.00049173
AB2057	AB19439	-0.78737	4.9822E-06
AB2057	AB19841	-0.71068	0.00009951
AB2057	AB21377	-0.75425	0.000020695
AB2057	AB21536	-0.74859	0.000025831
AB2057	AB21646	-0.86381	5.4143E-08
AB2057	AB21903	-0.75425	0.000020695
AB2057	AB22204	0.64357	0.0006914
AB2057	AB22647	0.68802	0.00020249
AB2057	AB22730	0.63225	0.00091753
AB2057	AB22852	0.63442	0.00086968
AB2057	AB23287	0.84107	2.6478E-07
AB2057	AB2417	0.74597	0.000028558
AB2057	AB24262	0.70458	0.00012125
AB2057	AB24263	0.69717	0.00015315
AB2057	AB25200	-0.79434	0.000003576
AB2057	AB2526	-0.68845	0.00019986
AB2057	AB26668	0.82876	5.6601E-07
AB2057	AB28110	-0.73028	0.000050917
AB2057	AB28115	0.8292	5.5155E-07
AB2057	AB28385	-0.87364	2.4903E-08
AB2057	AB28610	0.83399	4.1294E-07
AB2057	AB29320	0.80131	2.5341E-06
AB2057	AB29888	0.6305	0.00095742
AB2057	AB30418	0.66188	0.00042714
AB2057	AB33467	0.82057	9.0861E-07
AB2057	AB33890	-0.65752	0.00048041
AB2057	AB34172	0.74978	0.000024661
AB2057	AB34896	-0.65926	0.00045845
AB2057	AB35570	0.69978	0.00014115
AB2057	AB35757	0.84837	1.636E-07
AB2057	AB35792	0.6353	0.00085115
AB2057	AB36266	0.69717	0.00015315
AB2057	AB37554	0.76471	0.00001353
AB2057	AB38105	0.8427	2.3819E-07
AB2057	AB38191	-0.68497	0.00022177
AB2057	AB38635	0.84445	2.1252E-07
AB2057	AB38882	0.8732	2.5809E-08
AB2057	AB501	0.65665	0.00049173
AB2057	AB5129	-0.72941	0.000052519
AB2057	AB6398	-0.65055	0.00057763
AB2057	AB643	-0.71852	0.000076617
AB2057	AB6558	-0.79826	0.000002951
AB2057	AB9593	0.68192	0.00024262
AB20720	AB4600	-0.7313	0.000049098
AB20940	AB14901	-0.69478	0.00016489
AB20981	AB10398	-0.76348	0.000014239
AB20981	AB1126	-0.84435	2.1391E-07
AB20981	AB11547	0.78	6.9817E-06
AB20981	AB14084	0.9313	4.023E-11
AB20981	AB15695	0.77478	8.7997E-06
AB20981	AB18149	-0.90435	1.3501E-09
AB20981	AB28110	-0.66	0.0004494
AB20981	AB29432	0.89217	4.7572E-09
AB20981	AB30657	0.85304	1.186E-07
AB20981	AB30755	0.86348	5.5507E-08
AB20981	AB35151	-0.79739	3.0802E-06
AB20981	AB37776	0.89391	4.0114E-09
AB20981	AB38320	0.75391	0.000020974
AB20981	AB38744	-0.78783	4.8761E-06
AB20981	AB6494	-0.63913	0.00077359
AB20981	AB7155	0.87304	2.6152E-08
AB20981	AB8505	0.6713	0.00032907
AB21168	AB13425	0.64696	0.000634
AB21168	AB1949	-0.7713	0.000010233
AB21168	AB20152	-0.65217	0.00055353
AB21168	AB24002	-0.65217	0.00055353
AB21168	AB24131	-0.63652	0.00082565
AB21168	AB28961	0.63913	0.00077359
AB21168	AB33101	-0.71913	0.000075047
AB21168	AB5483	-0.78957	4.4932E-06
AB21168	AB6895	-0.7887	4.6812E-06
AB21168	AB9309	0.7313	0.000049098
AB21204	AB1126	0.81739	0.000001085
AB21204	AB13660	-0.72261	0.000066627
AB21204	AB14084	-0.75565	0.000019573
AB21204	AB18149	0.74435	0.000030377
AB21204	AB20981	-0.76957	0.000011025
AB21204	AB29432	-0.73391	0.0000447
AB21204	AB30657	-0.77913	7.2594E-06
AB21204	AB30755	-0.79652	3.2147E-06
AB21204	AB35151	0.71652	0.00008196
AB21204	AB37776	-0.84174	2.5355E-07
AB21204	AB38320	-0.81478	1.2519E-06
AB21204	AB38744	0.68	0.00025659
AB21204	AB7155	-0.75391	0.000020974
AB21204	AB8505	-0.83043	5.1223E-07
AB21377	AB10273	0.74087	0.00003462
AB21377	AB14901	-0.71826	0.000077292
AB21377	AB19841	0.79478	3.4995E-06
AB21377	AB29320	-0.66609	0.00038058
AB21377	AB34172	-0.67669	0.00028235
AB21377	AB5129	0.7313	0.000049098
AB21377	AB6558	0.67478	0.00029819
AB21536	AB10273	0.71304	0.000092043
AB21536	AB11547	-0.73391	0.0000447
AB21536	AB14084	-0.71391	0.000089426
AB21536	AB14283	0.71739	0.000079596
AB21536	AB14901	-0.76696	0.000012314
AB21536	AB18149	0.68087	0.00025017
AB21536	AB19841	0.84174	2.5355E-07
AB21536	AB21377	0.80348	2.2704E-06
AB21536	AB22668	0.68522	0.00022014
AB21536	AB29432	-0.69478	0.00016489
AB21536	AB34172	-0.7146	0.000087408
AB21536	AB37776	-0.74957	0.00002487
AB21536	AB38635	-0.65304	0.00054102
AB21536	AB6558	0.72348	0.000064657
AB21646	AB11344	0.83322	4.3271E-07
AB21646	AB1253	-0.71624	0.000082732
AB21646	AB12784	-0.67754	0.00027554
AB21646	AB12943	0.67362	0.00030817
AB21646	AB13162	0.64579	0.00065327
AB21646	AB14616	0.76973	0.000010947
AB21646	AB14975	0.808	1.7975E-06
AB21646	AB16898	0.74308	0.000031864
AB21646	AB17447	0.67145	0.00032772
AB21646	AB18208	-0.66101	0.00043725
AB21646	AB1945	0.6858	0.00021636
AB21646	AB19841	0.79322	3.7751E-06
AB21646	AB21536	0.66667	0.00037453
AB21646	AB22204	-0.6671	0.00037005
AB21646	AB22647	-0.7232	0.00006528
AB21646	AB22852	-0.73538	0.000042387
AB21646	AB23797	0.63318	0.00089671
AB21646	AB2417	-0.72755	0.000056095
AB21646	AB24262	-0.63405	0.00087772
AB21646	AB25200	0.90237	1.675E-09
AB21646	AB26668	-0.81366	1.3308E-06
AB21646	AB28110	0.67102	0.00033175
AB21646	AB28115	-0.83105	4.9365E-07
AB21646	AB28385	0.90715	9.8616E-10
AB21646	AB29320	-0.85627	9.4361E-08
AB21646	AB29888	-0.74581	0.00002873
AB21646	AB30418	-0.67449	0.00030065
AB21646	AB33467	-0.88972	6.0209E-09
AB21646	AB34172	-0.77708	7.9541E-06
AB21646	AB34896	0.68319	0.00023372
AB21646	AB35757	-0.78495	5.5723E-06
AB21646	AB35792	-0.65666	0.0004915
AB21646	AB36000	0.68754	0.00020542
AB21646	AB36266	-0.71972	0.000073553
AB21646	AB38105	-0.80061	2.6249E-06
AB21646	AB38191	0.70755	0.0001102
AB21646	AB38635	-0.90672	1.036E-09
AB21646	AB501	-0.66797	0.00036122
AB21646	AB5129	0.64797	0.00061767
AB21646	AB6398	0.71233	0.000094245
AB21646	AB643	0.79322	3.7751E-06
AB21646	AB6558	0.79626	3.2562E-06
AB21658	AB10398	0.65739	0.00048204
AB21658	AB12147	0.64609	0.00064835
AB21658	AB15695	-0.69565	0.00016052
AB21658	AB16227	-0.69391	0.00016935
AB21658	AB1696	-0.65739	0.00048204
AB21658	AB35792	0.77739	7.8442E-06
AB21903	AB11344	0.66174	0.00042872
AB21903	AB1253	-0.64435	0.00067789
AB21903	AB12784	-0.69565	0.00016052
AB21903	AB12943	0.72957	0.000052237
AB21903	AB13162	0.76435	0.000013735
AB21903	AB14283	0.68783	0.00020367
AB21903	AB14616	0.75217	0.000022462
AB21903	AB14788	0.64957	0.00059259
AB21903	AB14975	0.67391	0.00030566
AB21903	AB16898	0.80044	2.6475E-06
AB21903	AB17370	0.70435	0.00012214
AB21903	AB17447	0.69043	0.00018828
AB21903	AB18208	-0.79043	0.000004312
AB21903	AB19228	0.7687	0.000011441
AB21903	AB19439	0.66696	0.00037154
AB21903	AB19841	0.75652	0.000018904
AB21903	AB21377	0.66261	0.00041869
AB21903	AB21536	0.6713	0.00032907
AB21903	AB21646	0.74668	0.00002779
AB21903	AB22204	-0.74087	0.00003462
AB21903	AB22647	-0.71304	0.000092043
AB21903	AB22852	-0.76	0.000016426
AB21903	AB23287	-0.79258	3.8932E-06
AB21903	AB2417	-0.6487	0.00060612
AB21903	AB25200	0.76609	0.000012772
AB21903	AB2526	0.75739	0.000018255
AB21903	AB26668	-0.72348	0.000064657
AB21903	AB28110	0.74609	0.000028432
AB21903	AB28115	-0.74	0.000035759
AB21903	AB28385	0.78435	5.7298E-06
AB21903	AB28610	-0.69826	0.00014804
AB21903	AB29320	-0.81304	1.3755E-06
AB21903	AB29888	-0.72696	0.000057276
AB21903	AB30418	-0.64348	0.00069309
AB21903	AB33467	-0.81398	1.3075E-06
AB21903	AB34172	-0.67974	0.00025854
AB21903	AB34896	0.70261	0.0001291
AB21903	AB35757	-0.76261	0.00001476
AB21903	AB35792	-0.63913	0.00077359
AB21903	AB36266	-0.76696	0.000012314
AB21903	AB37554	-0.79304	3.8066E-06
AB21903	AB38105	-0.74957	0.00002487
AB21903	AB38191	0.7313	0.000049098
AB21903	AB38635	-0.84261	2.3966E-07
AB21903	AB5129	0.73217	0.000047592
AB21903	AB5879	0.66087	0.00043896
AB21903	AB643	0.77217	9.8569E-06
AB21903	AB6558	0.76696	0.000012314
AB21903	AB8595	0.66783	0.00036268
AB2202	AB33101	0.68087	0.00025017
AB22204	AB22852	0.79043	0.000004312
AB22218	AB11013	-0.69652	0.00015626
AB22218	AB14283	-0.73217	0.000047592
AB22218	AB16898	-0.62963	0.00097792
AB22218	AB17370	-0.68348	0.00023175
AB22218	AB19841	-0.66522	0.00038982
AB22218	AB22647	0.63043	0.00095901
AB22218	AB22852	0.68348	0.00023175
AB22218	AB2417	0.63739	0.00080797
AB22218	AB28110	-0.7087	0.00010616
AB22218	AB34172	0.72854	0.000054165
AB22218	AB36266	0.69304	0.00017392
AB22218	AB643	-0.6313	0.00093889
AB22218	AB6558	-0.68609	0.00021453
AB22647	AB14283	-0.68	0.00025659
AB22647	AB19841	-0.86783	3.9711E-08
AB22647	AB20008	-0.63217	0.00091914
AB22647	AB21377	-0.68609	0.00021453
AB22647	AB21536	-0.67043	0.00033721
AB22647	AB22852	0.63565	0.00084367
AB22647	AB25200	-0.77304	9.4926E-06
AB22647	AB29320	0.65652	0.00049337

AB22647	AB29888	0.64087	0.00074047
AB22647	AB34172	0.74641	0.000028087
AB22647	AB35757	0.76348	0.000014239
AB22647	AB36000	-0.72783	0.000055551
AB22647	AB36266	0.73826	0.000038137
AB22647	AB38635	0.6913	0.00018339
AB22647	AB6558	-0.76348	0.000014239
AB22668	AB10273	0.63304	0.00089975
AB22668	AB14283	0.69391	0.00016935
AB22668	AB14901	-0.7713	0.000010233
AB22668	AB19841	0.68	0.00025659
AB22668	AB20940	0.73391	0.0000447
AB22668	AB21377	0.63652	0.00082565
AB22668	AB35151	0.64957	0.00059259
AB22730	AB1253	0.64348	0.00069309
AB22730	AB19841	-0.65739	0.00048204
AB22730	AB20919	-0.65043	0.00057931
AB22730	AB21377	-0.84174	2.5355E-07
AB22730	AB29320	0.73391	0.0000447
AB22730	AB29888	0.6313	0.00093889
AB22730	AB3071	-0.67391	0.00030566
AB22730	AB31803	-0.77739	7.8442E-06
AB22730	AB34172	0.6732	0.00031187
AB22730	AB4160	-0.66174	0.00042872
AB22730	AB5129	-0.9	2.1562E-09
AB22852	AB6558	-0.78957	4.4932E-06
AB23201	AB14283	0.75391	0.000020974
AB23201	AB14788	0.73565	0.000041965
AB23201	AB14901	-0.80609	1.9857E-06
AB23201	AB19841	0.68435	0.00022588
AB23201	AB21536	0.73043	0.000050646
AB23201	AB22647	-0.69652	0.00015626
AB23201	AB22668	0.7713	0.000010233
AB23201	AB25552	0.73913	0.000036931
AB23201	AB35757	-0.68783	0.00020367
AB23201	AB8595	0.66783	0.00036268
AB23287	AB11013	-0.63748	0.00080624
AB23287	AB11344	-0.77724	7.8967E-06
AB23287	AB1253	0.81185	1.4662E-06
AB23287	AB12784	0.77856	7.4484E-06
AB23287	AB12943	-0.80484	2.1172E-06
AB23287	AB13162	-0.69137	0.00018303
AB23287	AB14160	-0.67516	0.00029502
AB23287	AB14616	-0.81799	1.0498E-06
AB23287	AB14975	-0.88108	1.3246E-08
AB23287	AB16799	-0.6734	0.00031011
AB23287	AB16898	-0.7864	5.2113E-06
AB23287	AB17370	-0.73562	0.000042016
AB23287	AB17447	-0.81054	1.5726E-06
AB23287	AB18208	0.72948	0.000052386
AB23287	AB19841	-0.74131	0.00003405
AB23287	AB21377	-0.67647	0.00028413
AB23287	AB21536	-0.64887	0.0006034
AB23287	AB21646	-0.88565	8.7964E-09
AB23287	AB22204	0.72598	0.00005927
AB23287	AB22647	0.69005	0.00019047
AB23287	AB22730	0.74438	0.000030338
AB23287	AB22852	0.76015	0.000016323
AB23287	AB23797	-0.6331	0.00089858
AB23287	AB2417	0.72773	0.000055735
AB23287	AB24263	0.67603	0.00028772
AB23287	AB25200	-0.84296	2.3424E-07
AB23287	AB2526	-0.76454	0.000013628
AB23287	AB26668	0.75358	0.00002125
AB23287	AB28110	-0.65588	0.00050189
AB23287	AB28115	0.871	3.0865E-08
AB23287	AB28385	-0.917	3.0098E-10
AB23287	AB29320	0.90298	1.5674E-09
AB23287	AB29888	0.79871	2.8859E-06
AB23287	AB30418	0.70364	0.00012496
AB23287	AB33467	0.89065	5.5087E-09
AB23287	AB33890	-0.69137	0.00018303
AB23287	AB34172	0.79979	2.7343E-06
AB23287	AB34896	-0.81755	1.0756E-06
AB23287	AB35757	0.77724	7.8967E-06
AB23287	AB35792	0.72773	0.000055735
AB23287	AB36266	0.7549	0.000020171
AB23287	AB38105	0.76454	0.000013628
AB23287	AB38191	-0.83639	3.5602E-07
AB23287	AB38635	0.87626	2.0033E-08
AB23287	AB4160	-0.65369	0.00053189
AB23287	AB501	0.70188	0.00013211
AB23287	AB5129	-0.83288	4.4181E-07
AB23287	AB5879	-0.66683	0.00037282
AB23287	AB6398	-0.65237	0.00055063
AB23287	AB643	-0.80835	1.7652E-06
AB23287	AB6558	-0.74526	0.000029346
AB23287	AB9593	0.73211	0.000047695
AB23737	AB10398	0.81391	1.3124E-06
AB23737	AB1126	0.72783	0.000055551
AB23737	AB11547	-0.74	0.000035759
AB23737	AB14084	-0.84696	1.799E-07
AB23737	AB15695	-0.83043	5.1223E-07
AB23737	AB18149	0.82783	5.9814E-07
AB23737	AB20981	-0.85652	9.2693E-08
AB23737	AB29350	0.79043	0.000004312
AB23737	AB29432	-0.84	2.835E-07
AB23737	AB30657	-0.7713	0.000010233
AB23737	AB30755	-0.7913	4.1373E-06
AB23737	AB35151	0.84435	2.1391E-07
AB23737	AB37776	-0.82957	5.3956E-07
AB23737	AB38744	0.79217	3.9689E-06
AB23737	AB7155	-0.85913	7.6721E-08
AB23737	AB9219	-0.65739	0.00048204
AB23797	AB1253	-0.66435	0.00039924
AB23797	AB13425	-0.80696	1.8982E-06
AB23797	AB22852	-0.78783	4.8761E-06
AB23797	AB24131	0.66957	0.00034552
AB23797	AB24262	-0.71304	0.000092043
AB23797	AB25200	0.6887	0.00019842
AB23797	AB26225	0.68261	0.00023776
AB23797	AB3253	0.75304	0.000021707
AB23797	AB33847	-0.76957	0.000011025
AB23797	AB34172	-0.65447	0.00052105
AB23797	AB35801	0.76783	0.00001187
AB23797	AB35827	0.8113	1.5098E-06
AB23797	AB36266	-0.71826	0.000077292
AB23797	AB6558	0.71913	0.000075047
AB24002	AB13425	-0.63304	0.00089975
AB24002	AB18669	-0.7687	0.000011441
AB24002	AB1949	0.63826	0.00079062
AB24002	AB20940	-0.65478	0.00051672
AB24002	AB23797	0.65739	0.00048204
AB24002	AB24131	0.91913	2.2853E-10
AB24002	AB26009	0.64783	0.00061993
AB24002	AB26225	0.82174	8.5053E-07
AB24002	AB28961	-0.80435	2.1717E-06
AB24002	AB31528	-0.73478	0.000043313
AB24002	AB3253	0.7	0.00014019
AB24002	AB33847	-0.66174	0.00042872
AB24002	AB35801	0.69913	0.00014407
AB24002	AB35827	0.76	0.000016426
AB24002	AB6639	0.69391	0.00016935
AB24002	AB6895	0.77913	7.2594E-06
AB24002	AB8577	0.63043	0.00095901
AB24002	AB8930	0.66087	0.00043896
AB24082	AB21204	0.64087	0.00074047
AB24082	AB21377	0.66261	0.00041869
AB24082	AB22218	-0.63391	0.00088071
AB24082	AB22612	-0.66696	0.00037154
AB24082	AB26668	-0.64522	0.00066298
AB24082	AB29432	-0.70522	0.00011879
AB24082	AB3282	0.66609	0.00038058
AB24082	AB35151	0.74087	0.00003462
AB24082	AB37776	-0.66435	0.00039924
AB24131	AB1949	0.73913	0.000036931
AB24131	AB26009	0.80261	0.000002373
AB24131	AB26225	0.70957	0.0001032
AB24131	AB38320	-0.69304	0.00017392
AB24131	AB6895	0.75304	0.000021707
AB2417	AB1253	0.80348	2.2704E-06
AB2417	AB13425	0.87304	2.6152E-08
AB2417	AB14160	-0.6887	0.00019842
AB2417	AB14788	-0.65391	0.00052875
AB2417	AB19841	-0.70435	0.00012214
AB2417	AB21536	-0.65304	0.00054102
AB2417	AB22204	0.7487	0.000025721
AB2417	AB22852	0.83565	3.7269E-07
AB2417	AB23797	-0.77304	9.4926E-06
AB2417	AB24262	0.76435	0.000013735
AB2417	AB25200	-0.73652	0.000040653
AB2417	AB29320	0.71565	0.000084386
AB2417	AB34172	0.88192	1.23E-08
AB2417	AB35757	0.76957	0.000011025
AB2417	AB35827	-0.65391	0.00052875
AB2417	AB36266	0.8113	1.5098E-06
AB2417	AB6558	-0.75565	0.000019573
AB24262	AB13425	0.77304	9.4926E-06
AB24262	AB27392	0.65391	0.00052875
AB24262	AB34172	0.64096	0.00073878
AB24262	AB38320	0.68348	0.00023175
AB24262	AB6558	-0.69478	0.00016489
AB24263	AB11344	-0.74957	0.00002487
AB24263	AB12784	0.74696	0.000027502
AB24263	AB14616	-0.68087	0.00025017
AB24263	AB14788	-0.84261	2.3966E-07
AB24263	AB14975	-0.72174	0.00006865
AB24263	AB15099	-0.71652	0.00008196
AB24263	AB15541	-0.82087	8.9343E-07
AB24263	AB16227	-0.79217	3.9689E-06
AB24263	AB16898	-0.63791	0.00079758
AB24263	AB1696	-0.78	6.9817E-06
AB24263	AB17447	-0.66783	0.00036268
AB24263	AB17891	-0.67652	0.00028371
AB24263	AB21536	-0.63304	0.00089975
AB24263	AB21658	0.65478	0.00051672
AB24263	AB22204	0.78783	4.8761E-06
AB24263	AB2526	-0.64696	0.000634
AB24263	AB25552	-0.72957	0.000052237
AB24263	AB26668	0.64261	0.00070859
AB24263	AB29320	0.68261	0.00023776
AB24263	AB29749	0.65913	0.00046006
AB24263	AB3071	-0.70087	0.00013641
AB24263	AB31803	-0.72348	0.000064657
AB24263	AB33467	0.64193	0.00072093
AB24263	AB35757	0.65913	0.00046006
AB24263	AB35792	0.81304	1.3755E-06
AB24263	AB36667	0.63652	0.00082565
AB24263	AB38105	0.64609	0.00064835
AB24263	AB38635	0.67478	0.00029819
AB24263	AB5129	-0.70696	0.00011232
AB24263	AB6639	0.64	0.00075688
AB24263	AB836	-0.65652	0.00049337
AB24263	AB8930	0.65565	0.00050493
AB24263	AB9798	-0.81913	9.8508E-07
AB24397	AB13425	-0.77826	7.5467E-06
AB24397	AB13660	-0.67826	0.00026986
AB24397	AB18669	-0.64957	0.00059259
AB24397	AB22852	-0.62957	0.00097949
AB24397	AB23797	0.85739	8.7066E-08
AB24397	AB24002	0.87565	2.1077E-08
AB24397	AB24131	0.88522	9.1514E-09
AB24397	AB2417	-0.64261	0.00070859
AB24397	AB24262	-0.70609	0.00011552
AB24397	AB26009	0.68348	0.00023175
AB24397	AB26225	0.81478	1.2519E-06
AB24397	AB27392	-0.74696	0.000027502
AB24397	AB2747	0.68696	0.00020904
AB24397	AB28961	-0.75652	0.000018904
AB24397	AB3253	0.76261	0.00001476
AB24397	AB33847	-0.73217	0.000047592
AB24397	AB35801	0.7313	0.000049098
AB24397	AB35827	0.80696	1.8982E-06
AB24397	AB38320	-0.69652	0.00015626
AB24397	AB6398	0.76087	0.000015853
AB24397	AB6558	0.63304	0.00089975
AB24397	AB6895	0.65565	0.00050493
AB24397	AB9309	-0.76609	0.000012772
AB24846	AB17891	0.75739	0.000018255
AB25200	AB11344	0.66	0.0004494
AB25200	AB1945	0.65652	0.00049337
AB25200	AB19841	0.78087	6.7135E-06
AB25200	AB22852	-0.7913	4.1373E-06
AB25200	AB29320	-0.77652	8.1519E-06
AB25200	AB29888	-0.70174	0.00013271
AB25200	AB34172	-0.75294	0.000021793
AB25200	AB36000	0.74522	0.00002939
AB25200	AB38635	-0.79304	3.8066E-06
AB25200	AB5129	0.65043	0.00057931
AB25200	AB6558	0.86	7.1974E-08
AB2526	AB10273	0.74261	0.000032437
AB2526	AB11013	0.8313	4.8615E-07
AB2526	AB11344	0.68609	0.00021453
AB2526	AB1253	-0.68783	0.00020367
AB2526	AB12784	-0.78522	5.5048E-06
AB2526	AB12943	0.7713	0.000010233
AB2526	AB14160	0.63304	0.00089975
AB2526	AB14283	0.65391	0.00052875
AB2526	AB14616	0.86696	4.2501E-08
AB2526	AB14788	0.71565	0.000084386
AB2526	AB14901	-0.64261	0.00070859
AB2526	AB14975	0.83652	3.5307E-07
AB2526	AB16227	0.66696	0.00037154
AB2526	AB16898	0.88802	7.0676E-09

AB2526	AB17447	0.91043	6.7432E-10
AB2526	AB19841	0.78435	5.7298E-06
AB2526	AB21377	0.79043	0.000004312
AB2526	AB21536	0.69826	0.00014804
AB2526	AB21646	0.68711	0.00020811
AB2526	AB22204	-0.75391	0.000020974
AB2526	AB22647	-0.70696	0.00011232
AB2526	AB22668	0.64348	0.00069309
AB2526	AB22730	-0.80435	2.1717E-06
AB2526	AB22852	-0.67565	0.00029087
AB2526	AB23201	0.70348	0.00012558
AB2526	AB2417	-0.65304	0.00054102
AB2526	AB25200	0.65217	0.00055353
AB2526	AB26668	-0.84348	2.2646E-07
AB2526	AB28110	0.73913	0.000036931
AB2526	AB28115	-0.76174	0.000015298
AB2526	AB28385	0.78522	5.5048E-06
AB2526	AB29320	-0.82348	7.7019E-07
AB2526	AB29888	-0.64696	0.000634
AB2526	AB3071	0.76348	0.000014239
AB2526	AB31803	0.69391	0.00016935
AB2526	AB33467	-0.85678	9.101E-08
AB2526	AB34172	-0.80305	2.3202E-06
AB2526	AB34896	0.73565	0.000041965
AB2526	AB35757	-0.82957	5.3956E-07
AB2526	AB35792	-0.76522	0.000013246
AB2526	AB36266	-0.75652	0.000018904
AB2526	AB38105	-0.84957	1.5082E-07
AB2526	AB38191	0.80957	1.6557E-06
AB2526	AB38635	-0.71652	0.00008196
AB2526	AB501	-0.68783	0.00020367
AB2526	AB5129	0.84435	2.1391E-07
AB2526	AB5879	0.74435	0.000030377
AB2526	AB643	0.71478	0.000086874
AB2526	AB8595	0.86	7.1974E-08
AB2526	AB9593	-0.68348	0.00023175
AB25363	AB11013	0.71652	0.00008196
AB25363	AB14084	-0.63043	0.00095901
AB25363	AB14283	0.73304	0.000046126
AB25363	AB14616	0.66348	0.00040887
AB25363	AB14788	0.70696	0.00011232
AB25363	AB14901	-0.72087	0.000070727
AB25363	AB16898	0.6671	0.00037003
AB25363	AB17370	0.76522	0.000013246
AB25363	AB18149	0.70783	0.0001092
AB25363	AB19841	0.72609	0.000059048
AB25363	AB21536	0.80783	1.8141E-06
AB25363	AB22218	-0.63913	0.00077359
AB25363	AB22647	-0.64957	0.00059259
AB25363	AB22668	0.72609	0.000059048
AB25363	AB23201	0.88435	9.9019E-09
AB25363	AB25552	0.77565	8.4703E-06
AB25363	AB26668	-0.70522	0.00011879
AB25363	AB27281	0.82	9.3826E-07
AB25363	AB28110	0.81652	1.1383E-06
AB25363	AB34172	-0.65185	0.00055821
AB25363	AB35757	-0.76348	0.000014239
AB25363	AB37554	-0.64435	0.00067789
AB25363	AB37776	-0.64348	0.00069309
AB25363	AB38105	-0.73739	0.000039377
AB25363	AB4262	-0.84106	2.6496E-07
AB25552	AB14788	0.80609	1.9857E-06
AB25552	AB14901	-0.63478	0.00086202
AB25552	AB21536	0.68261	0.00023776
AB25552	AB6639	-0.63739	0.00080797
AB25552	AB9798	0.80783	1.8141E-06
AB26668	AB10273	-0.80957	1.6557E-06
AB26668	AB11013	-0.80609	1.9857E-06
AB26668	AB11344	-0.7	0.00014019
AB26668	AB1253	0.69652	0.00015626
AB26668	AB14283	-0.68435	0.00022588
AB26668	AB14616	-0.88609	8.4523E-09
AB26668	AB14901	0.70609	0.00011552
AB26668	AB16898	-0.86885	3.6644E-08
AB26668	AB17447	-0.76	0.000016426
AB26668	AB19841	-0.88261	1.1571E-08
AB26668	AB21377	-0.85391	1.1158E-07
AB26668	AB21536	-0.8687	3.7086E-08
AB26668	AB22204	0.64522	0.00066298
AB26668	AB22647	0.75826	0.000017626
AB26668	AB22668	-0.63565	0.00084367
AB26668	AB22730	0.66783	0.00036268
AB26668	AB22852	0.65565	0.00050493
AB26668	AB23201	-0.68348	0.00023175
AB26668	AB2417	0.74261	0.000032437
AB26668	AB25200	-0.70087	0.00013641
AB26668	AB28110	-0.85739	8.7066E-08
AB26668	AB28115	0.81043	1.5813E-06
AB26668	AB29320	0.79826	2.9507E-06
AB26668	AB34172	0.84663	1.8394E-07
AB26668	AB34896	-0.67739	0.00027671
AB26668	AB35757	0.92609	8.7812E-11
AB26668	AB35792	0.64435	0.00067789
AB26668	AB36266	0.80522	2.0769E-06
AB26668	AB38105	0.96087	9.4675E-14
AB26668	AB38191	-0.63826	0.00079062
AB26668	AB38635	0.75652	0.000018904
AB26668	AB5129	-0.66	0.0004494
AB26668	AB643	-0.72957	0.000052237
AB26668	AB6558	-0.7687	0.000011441
AB26668	AB8595	-0.69913	0.00014407
AB27281	AB13162	0.67304	0.0003133
AB27281	AB14283	0.64348	0.00069309
AB27281	AB14616	0.70609	0.00011552
AB27281	AB14788	0.77217	9.8569E-06
AB27281	AB16898	0.63965	0.00076351
AB27281	AB17370	0.72174	0.00006865
AB27281	AB17447	0.67304	0.0003133
AB27281	AB19841	0.70348	0.00012558
AB27281	AB21536	0.68087	0.00025017
AB27281	AB22204	-0.64348	0.00069309
AB27281	AB22647	-0.63478	0.00086202
AB27281	AB23201	0.7	0.00014019
AB27281	AB2417	-0.70435	0.00012214
AB27281	AB24263	-0.65391	0.00052875
AB27281	AB25200	0.66783	0.00036268
AB27281	AB2526	0.64696	0.000634
AB27281	AB25552	0.85913	7.6721E-08
AB27281	AB28110	0.72957	0.000052237
AB27281	AB28385	0.69043	0.00018828
AB27281	AB34172	-0.69368	0.00017055
AB27281	AB35757	-0.70435	0.00012214
AB27281	AB35792	-0.66348	0.00040887
AB27281	AB37554	-0.68957	0.0001933
AB27281	AB38105	-0.64609	0.00064835
AB27281	AB5129	0.67217	0.0003211
AB27281	AB9798	0.65478	0.00051672
AB27392	AB12147	-0.64174	0.00072438
AB27392	AB1491	0.63391	0.00088071
AB27392	AB1696	0.63391	0.00088071
AB27392	AB26225	-0.70783	0.0001092
AB27392	AB31424	-0.7687	0.000011441
AB27392	AB36000	-0.65304	0.00054102
AB27392	AB36667	-0.7087	0.00010616
AB27392	AB6558	-0.63043	0.00095901
AB2747	AB1945	0.65565	0.00050493
AB2747	AB22852	-0.66	0.0004494
AB2747	AB23797	0.68609	0.00021453
AB2747	AB25200	0.71043	0.0001003
AB2747	AB26225	0.68	0.00025659
AB2747	AB27392	-0.67565	0.00029087
AB2747	AB36000	0.67826	0.00026986
AB2747	AB6558	0.76783	0.00001187
AB27638	AB1253	-0.67391	0.00030566
AB27638	AB14160	0.71913	0.000075047
AB27638	AB20152	0.66522	0.00038982
AB27638	AB22852	-0.71478	0.000086874
AB27638	AB23797	0.70957	0.0001032
AB27638	AB26225	0.66522	0.00038982
AB27638	AB33847	-0.71739	0.000079596
AB27638	AB34172	-0.63486	0.00086037
AB27638	AB34896	0.64174	0.00072438
AB27638	AB36266	-0.65043	0.00057931
AB27638	AB4160	0.66174	0.00042872
AB28110	AB10273	0.66174	0.00042872
AB28110	AB11547	-0.82783	5.9814E-07
AB28110	AB14084	-0.79739	3.0802E-06
AB28110	AB14283	0.72261	0.000066627
AB28110	AB17447	0.6913	0.00018339
AB28110	AB18149	0.71217	0.000094728
AB28110	AB19841	0.89217	4.7572E-09
AB28110	AB21377	0.79043	0.000004312
AB28110	AB21536	0.8913	5.1749E-09
AB28110	AB22647	-0.77043	0.000010623
AB28110	AB22668	0.69304	0.00017392
AB28110	AB22852	-0.66087	0.00043896
AB28110	AB23201	0.72087	0.000070727
AB28110	AB2417	-0.74	0.000035759
AB28110	AB25200	0.69478	0.00016489
AB28110	AB28115	-0.72261	0.000066627
AB28110	AB29432	-0.74957	0.00002487
AB28110	AB34172	-0.7817	6.4656E-06
AB28110	AB35151	0.65217	0.00055353
AB28110	AB35757	-0.88174	1.2497E-08
AB28110	AB36000	0.72783	0.000055551
AB28110	AB36266	-0.83478	3.9327E-07
AB28110	AB37776	-0.7713	0.000010233
AB28110	AB38105	-0.84696	1.799E-07
AB28110	AB5879	0.6913	0.00018339
AB28110	AB6558	0.85913	7.6721E-08
AB28115	AB11344	-0.65826	0.00047094
AB28115	AB1253	0.80435	2.1717E-06
AB28115	AB17447	-0.78174	6.4545E-06
AB28115	AB18208	0.70696	0.00011232
AB28115	AB1945	-0.70957	0.0001032
AB28115	AB19841	-0.78609	5.2877E-06
AB28115	AB21377	-0.79565	3.3545E-06
AB28115	AB21536	-0.64609	0.00064835
AB28115	AB22647	0.73391	0.0000447
AB28115	AB22730	0.75652	0.000018904
AB28115	AB22852	0.6887	0.00019842
AB28115	AB2409	-0.65913	0.00046006
AB28115	AB2417	0.67913	0.00026315
AB28115	AB25200	-0.82696	6.2951E-07
AB28115	AB29320	0.82957	5.3956E-07
AB28115	AB29888	0.79043	0.000004312
AB28115	AB30418	0.77304	9.4926E-06
AB28115	AB34172	0.80697	1.8965E-06
AB28115	AB35757	0.82	9.3826E-07
AB28115	AB36000	-0.68783	0.00020367
AB28115	AB36266	0.7113	0.000097481
AB28115	AB38635	0.79826	2.9507E-06
AB28115	AB4160	-0.71217	0.000094728
AB28115	AB5129	-0.73565	0.000041965
AB28115	AB6558	-0.82435	7.3262E-07
AB28385	AB10273	0.6513	0.00056629
AB28385	AB11013	0.69391	0.00016935
AB28385	AB11344	0.79043	0.000004312
AB28385	AB1253	-0.71478	0.000086874
AB28385	AB13162	0.72	0.000072859
AB28385	AB14283	0.62957	0.00097949
AB28385	AB14616	0.8513	1.3385E-07
AB28385	AB16898	0.76732	0.000012126
AB28385	AB17447	0.82261	8.0947E-07
AB28385	AB18208	-0.74609	0.000028432
AB28385	AB19841	0.85739	8.7066E-08
AB28385	AB21377	0.7487	0.000025721
AB28385	AB21536	0.71217	0.000094728
AB28385	AB22204	-0.68261	0.00023776
AB28385	AB22647	-0.81304	1.3755E-06
AB28385	AB22730	-0.72174	0.00006865
AB28385	AB22852	-0.73217	0.000047592
AB28385	AB2417	-0.75304	0.000021707
AB28385	AB25200	0.87826	1.6904E-08
AB28385	AB26668	-0.82261	8.0947E-07
AB28385	AB28110	0.7687	0.000011441
AB28385	AB28115	-0.8687	3.7086E-08
AB28385	AB29320	-0.82348	7.7019E-07
AB28385	AB29888	-0.77652	8.1519E-06
AB28385	AB30418	-0.75478	0.000020263
AB28385	AB34172	-0.81307	1.3732E-06
AB28385	AB34896	0.72348	0.000064657
AB28385	AB35757	-0.82522	6.9669E-07
AB28385	AB35792	-0.64174	0.00072438
AB28385	AB36000	0.6313	0.00093889
AB28385	AB36266	-0.77391	9.1403E-06
AB28385	AB38105	-0.82435	7.3262E-07
AB28385	AB38191	0.7487	0.000025721
AB28385	AB38635	-0.86087	6.7492E-08
AB28385	AB5129	0.80435	2.1717E-06
AB28385	AB5879	0.70609	0.00011552
AB28385	AB6398	0.68957	0.0001933
AB28385	AB643	0.80696	1.8982E-06
AB28385	AB6558	0.81565	1.1939E-06
AB28385	AB9593	-0.65913	0.00046006
AB28610	AB11013	-0.69043	0.00018828
AB28610	AB11344	-0.73217	0.000047592
AB28610	AB1253	0.76957	0.000011025
AB28610	AB12784	0.74087	0.00003462
AB28610	AB12943	-0.64609	0.00064835
AB28610	AB13162	-0.63913	0.00077359
AB28610	AB14160	-0.73826	0.000038137
AB28610	AB14616	-0.76522	0.000013246
AB28610	AB14788	-0.63826	0.00079062

AB28610	AB14975	-0.84609	1.9066E-07
AB28610	AB16898	-0.78562	5.4026E-06
AB28610	AB17370	-0.76783	0.00001187
AB28610	AB17447	-0.73652	0.000040653
AB28610	AB19841	-0.71913	0.000075047
AB28610	AB21377	-0.69478	0.00016489
AB28610	AB21536	-0.7087	0.00010616
AB28610	AB21646	-0.86628	4.4809E-08
AB28610	AB22204	0.71739	0.000079596
AB28610	AB22218	0.68522	0.00022014
AB28610	AB22647	0.63217	0.00091914
AB28610	AB22730	0.69826	0.00014804
AB28610	AB22852	0.74	0.000035759
AB28610	AB23287	0.87144	2.9795E-08
AB28610	AB2417	0.76348	0.000014239
AB28610	AB24262	0.65739	0.00048204
AB28610	AB24263	0.73826	0.000038137
AB28610	AB25200	-0.73826	0.000038137
AB28610	AB2526	-0.7513	0.000023241
AB28610	AB26668	0.86087	6.7492E-08
AB28610	AB28110	-0.67913	0.00026315
AB28610	AB28115	0.83913	2.9964E-07
AB28610	AB28385	-0.81565	1.1939E-06
AB28610	AB29320	0.89217	4.7572E-09
AB28610	AB29888	0.67826	0.00026986
AB28610	AB33467	0.87686	1.9035E-08
AB28610	AB33890	-0.70087	0.00013641
AB28610	AB34172	0.83747	3.3264E-07
AB28610	AB34896	-0.73826	0.000038137
AB28610	AB35757	0.80957	1.6557E-06
AB28610	AB35792	0.73043	0.000050646
AB28610	AB36266	0.73652	0.000040653
AB28610	AB38105	0.84261	2.3966E-07
AB28610	AB38191	-0.69391	0.00016935
AB28610	AB38635	0.7713	0.000010233
AB28610	AB501	0.68696	0.00020904
AB28610	AB5129	-0.73478	0.000043313
AB28610	AB643	-0.81565	1.1939E-06
AB28610	AB6490	0.63565	0.00084367
AB28610	AB6558	-0.73739	0.000039377
AB28610	AB9593	0.71391	0.000089426
AB28820	AB11277	0.72174	0.00006865
AB28820	AB11702	0.67652	0.00028371
AB28820	AB12943	-0.71565	0.000084386
AB28820	AB14975	-0.67652	0.00028371
AB28820	AB16898	-0.65883	0.00046386
AB28820	AB17447	-0.74609	0.000028432
AB28820	AB18208	0.67565	0.00029087
AB28820	AB21377	-0.70783	0.0001092
AB28820	AB22730	0.85652	9.2693E-08
AB28820	AB23287	0.6607	0.00044099
AB28820	AB2409	-0.63478	0.00086202
AB28820	AB2526	-0.7	0.00014019
AB28820	AB28115	0.69478	0.00016489
AB28820	AB28385	-0.65826	0.00047094
AB28820	AB29320	0.72522	0.000060868
AB28820	AB29888	0.71478	0.000086874
AB28820	AB30418	0.67652	0.00028371
AB28820	AB33467	0.7214	0.000069444
AB28820	AB35208	-0.71826	0.000077292
AB28820	AB38191	-0.66087	0.00043896
AB28820	AB4160	-0.77652	8.1519E-06
AB28820	AB501	0.71913	0.000075047
AB28820	AB5129	-0.75565	0.000019573
AB28820	AB5879	-0.64087	0.00074047
AB28820	AB6490	0.65478	0.00051672
AB28820	AB8595	-0.70957	0.0001032
AB28820	AB9593	0.66087	0.00043896
AB28961	AB13425	0.64	0.00075688
AB28961	AB1949	-0.74261	0.000032437
AB28961	AB24131	-0.83217	4.6126E-07
AB28961	AB26009	-0.6687	0.00035401
AB28961	AB26225	-0.6913	0.00018339
AB28961	AB31528	0.68957	0.0001933
AB28961	AB38320	0.71217	0.000094728
AB28961	AB6895	-0.7087	0.00010616
AB29074	AB1949	0.67304	0.0003133
AB29076	AB1126	0.70087	0.00013641
AB29076	AB1949	0.6313	0.00093889
AB29076	AB24131	0.66348	0.00040887
AB29076	AB26009	0.63913	0.00077359
AB29076	AB30657	-0.71565	0.000084386
AB29076	AB30755	-0.64696	0.000634
AB29076	AB31456	0.72174	0.00006865
AB29076	AB38744	0.78957	4.4932E-06
AB29076	AB6494	0.82522	6.9669E-07
AB29320	AB11344	-0.77913	7.2594E-06
AB29320	AB19841	-0.73217	0.000047592
AB29320	AB22204	0.82609	6.6234E-07
AB29320	AB22852	0.83043	5.1223E-07
AB29320	AB29888	0.79826	2.9507E-06
AB29320	AB30418	0.63739	0.00080797
AB29320	AB38635	0.83913	2.9964E-07
AB29320	AB4160	-0.72435	0.000062737
AB29320	AB5129	-0.79391	3.6502E-06
AB29320	AB6558	-0.68957	0.0001933
AB29350	AB10398	0.65304	0.00054102
AB29350	AB1126	0.67913	0.00026315
AB29350	AB14084	-0.68087	0.00025017
AB29350	AB15695	-0.77391	9.1403E-06
AB29350	AB16638	0.71146	0.000096986
AB29350	AB20981	-0.72957	0.000052237
AB29350	AB29432	-0.66957	0.00034552
AB29350	AB30657	-0.67391	0.00030566
AB29350	AB30755	-0.74435	0.000030377
AB29350	AB35151	0.64261	0.00070859
AB29350	AB37776	-0.67739	0.00027671
AB29350	AB38744	0.74261	0.000032437
AB29350	AB7155	-0.7513	0.000023241
AB29350	AB9219	-0.62957	0.00097949
AB29432	AB14283	-0.63043	0.00095901
AB29432	AB18149	-0.87739	1.8204E-08
AB29432	AB19841	-0.6487	0.00060612
AB29432	AB30657	0.78348	5.9629E-06
AB29432	AB35151	-0.89043	5.6254E-09
AB29432	AB36000	-0.64087	0.00074047
AB29432	AB37776	0.88783	7.1964E-09
AB29432	AB38320	0.71043	0.0001003
AB29568	AB24131	-0.63913	0.00077359
AB29749	AB14160	-0.71565	0.000084386
AB29749	AB14788	-0.77826	7.5467E-06
AB29749	AB16227	-0.76783	0.00001187
AB29749	AB19623	-0.82174	8.5053E-07
AB29749	AB22204	0.87739	1.8204E-08
AB29749	AB22852	0.67304	0.0003133
AB29749	AB29320	0.66435	0.00039924
AB29749	AB3071	-0.64783	0.00061993
AB29749	AB31803	-0.70348	0.00012558
AB29749	AB35792	0.66957	0.00034552
AB29749	AB4035	-0.66783	0.00036268
AB29749	AB5129	-0.69391	0.00016935
AB29888	AB30418	0.88783	7.1964E-09
AB29888	AB35208	-0.66	0.0004494
AB30657	AB18149	-0.83478	3.9327E-07
AB30657	AB37776	0.78957	4.4932E-06
AB30657	AB38320	0.84435	2.1391E-07
AB3071	AB12147	-0.74174	0.000033513
AB3071	AB22204	-0.65391	0.00052875
AB3071	AB31803	0.85739	8.7066E-08
AB3071	AB36667	-0.78174	6.4545E-06
AB30755	AB10398	-0.76696	0.000012314
AB30755	AB14084	0.80348	2.2704E-06
AB30755	AB15695	0.80522	2.0769E-06
AB30755	AB18149	-0.77043	0.000010623
AB30755	AB29432	0.72783	0.000055551
AB30755	AB30657	0.86957	3.4618E-08
AB30755	AB35151	-0.70087	0.00013641
AB30755	AB37776	0.83652	3.5307E-07
AB30755	AB38320	0.64261	0.00070859
AB30977	AB11344	0.65478	0.00051672
AB30977	AB1253	-0.6513	0.00056629
AB30977	AB12784	-0.72087	0.000070727
AB30977	AB12943	0.72783	0.000055551
AB30977	AB14616	0.68783	0.00020367
AB30977	AB14975	0.77826	7.5467E-06
AB30977	AB15695	0.72087	0.000070727
AB30977	AB16799	0.69652	0.00015626
AB30977	AB17447	0.67652	0.00028371
AB30977	AB19878	-0.74348	0.000031392
AB30977	AB23287	-0.75446	0.000020525
AB30977	AB25200	0.63217	0.00091914
AB30977	AB28385	0.64261	0.00070859
AB30977	AB29320	-0.68783	0.00020367
AB30977	AB31456	-0.68696	0.00020904
AB30977	AB33467	-0.6987	0.00014604
AB30977	AB34896	0.77652	8.1519E-06
AB30977	AB35792	-0.71652	0.00008196
AB30977	AB38191	0.70522	0.00011879
AB30977	AB38635	-0.68261	0.00023776
AB30977	AB38882	-0.6713	0.00032907
AB30977	AB501	-0.73391	0.0000447
AB30977	AB5129	0.67565	0.00029087
AB30977	AB9593	-0.8087	1.7333E-06
AB31424	AB12147	0.89217	4.7572E-09
AB31424	AB1491	-0.73913	0.000036931
AB31424	AB30657	-0.72435	0.000062737
AB31424	AB36667	0.78696	5.0782E-06
AB31456	AB1126	0.63565	0.00084367
AB31456	AB30657	-0.64087	0.00074047
AB31456	AB31424	0.63739	0.00080797
AB31456	AB35155	-0.65304	0.00054102
AB31456	AB38744	0.67913	0.00026315
AB31456	AB6494	0.79478	3.4995E-06
AB31528	AB24131	-0.66348	0.00040887
AB31528	AB6895	-0.70174	0.00013271
AB31803	AB36667	-0.67304	0.0003133
AB31928	AB30755	-0.65565	0.00050493
AB31928	AB33847	0.67217	0.0003211
AB31928	AB35801	-0.65043	0.00057931
AB31928	AB7155	-0.67391	0.00030566
AB3253	AB24131	0.69304	0.00017392
AB3253	AB27392	-0.65217	0.00055353
AB32798	AB20940	0.71652	0.00008196
AB32798	AB24002	-0.6487	0.00060612
AB32798	AB28961	0.64174	0.00072438
AB32798	AB29568	0.73652	0.000040653
AB32798	AB8577	-0.62957	0.00097949
AB3282	AB14901	-0.68174	0.0002439
AB3282	AB15257	-0.63478	0.00086202
AB3282	AB19841	0.63217	0.00091914
AB3282	AB21377	0.79217	3.9689E-06
AB3282	AB21536	0.6913	0.00018339
AB3282	AB26668	-0.7	0.00014019
AB3282	AB28110	0.64	0.00075688
AB3282	AB29432	-0.64696	0.000634
AB3282	AB34451	-0.78261	6.2044E-06
AB3282	AB35151	0.69652	0.00015626
AB3282	AB38105	-0.70957	0.0001032
AB3282	AB6490	-0.67043	0.00033721
AB33101	AB15257	0.63391	0.00088071
AB33101	AB1949	0.63043	0.00095901
AB33101	AB5483	0.73913	0.000036931
AB33101	AB6895	0.76435	0.000013735
AB33101	AB8577	0.78435	5.7298E-06
AB33101	AB9309	-0.64087	0.00074047
AB33467	AB10273	-0.68909	0.00019609
AB33467	AB11013	-0.71005	0.00010157
AB33467	AB11344	-0.82621	6.5765E-07
AB33467	AB1253	0.82184	8.4558E-07
AB33467	AB12943	-0.8297	5.3517E-07
AB33467	AB13162	-0.66376	0.00040571
AB33467	AB14160	-0.66289	0.00041551
AB33467	AB14616	-0.86813	3.8773E-08
AB33467	AB16898	-0.88753	7.398E-09
AB33467	AB17447	-0.82359	7.6533E-07
AB33467	AB18208	0.66813	0.00035965
AB33467	AB19841	-0.82359	7.6533E-07
AB33467	AB21377	-0.75197	0.000022641
AB33467	AB21536	-0.67337	0.00031042
AB33467	AB22204	0.77293	9.5382E-06
AB33467	AB22647	0.70743	0.00011061
AB33467	AB22730	0.75285	0.000021876
AB33467	AB22852	0.79913	2.8255E-06
AB33467	AB2417	0.73625	0.000041056
AB33467	AB25200	-0.82272	8.0456E-07
AB33467	AB26668	0.8559	9.689E-08
AB33467	AB28110	-0.67162	0.00032613
AB33467	AB28115	0.87075	3.1491E-08
AB33467	AB28385	-0.87075	3.1491E-08
AB33467	AB29320	0.96682	1.585E-14
AB33467	AB29888	0.79302	3.8107E-06
AB33467	AB30418	0.69433	0.0001672
AB33467	AB34172	0.83851	3.1156E-07
AB33467	AB34896	-0.82796	5.936E-07
AB33467	AB35757	0.83669	3.494E-07
AB33467	AB35792	0.77992	7.0068E-06
AB33467	AB36266	0.79651	3.2159E-06
AB33467	AB38105	0.8559	9.689E-08
AB33467	AB38191	-0.8332	4.3347E-07
AB33467	AB38635	0.86114	6.6132E-08
AB33467	AB4160	-0.70743	0.00011061
AB33467	AB5129	-0.77905	7.2866E-06
AB33467	AB5879	-0.70743	0.00011061
AB33467	AB643	-0.8166	1.1333E-06

AB33467	AB6558	-0.73188	0.000048088
AB33467	AB8595	-0.73276	0.000046603
AB33467	AB9593	0.74411	0.000030649
AB33847	AB13425	0.64435	0.00067789
AB33847	AB26225	-0.67304	0.0003133
AB33847	AB27167	0.63217	0.00091914
AB33890	AB1253	-0.70261	0.0001291
AB33890	AB12784	-0.69391	0.00016935
AB33890	AB13162	0.71391	0.000089426
AB33890	AB13425	-0.77826	7.5467E-06
AB33890	AB13660	-0.79652	3.2147E-06
AB33890	AB14160	0.74609	0.000028432
AB33890	AB14788	0.6913	0.00018339
AB33890	AB19623	0.67913	0.00026315
AB33890	AB21646	0.65927	0.00045829
AB33890	AB22204	-0.84087	2.6815E-07
AB33890	AB22852	-0.78174	6.4545E-06
AB33890	AB23797	0.76957	0.000011025
AB33890	AB2417	-0.84348	2.2646E-07
AB33890	AB24262	-0.80609	1.9857E-06
AB33890	AB24263	-0.63652	0.00082565
AB33890	AB28385	0.63652	0.00082565
AB33890	AB29320	-0.69478	0.00016489
AB33890	AB29749	-0.7713	0.000010233
AB33890	AB33847	-0.64609	0.00064835
AB33890	AB34172	-0.72636	0.00005848
AB33890	AB34896	0.67391	0.00030566
AB33890	AB36266	-0.74087	0.00003462
AB33890	AB38635	-0.65304	0.00054102
AB33890	AB6398	0.72348	0.000064657
AB33890	AB643	0.71565	0.000084386
AB34172	AB10273	-0.63617	0.00083296
AB34172	AB13425	0.66972	0.00034404
AB34172	AB14283	-0.72288	0.000066012
AB34172	AB19841	-0.8427	2.3819E-07
AB34172	AB22204	0.70893	0.00010534
AB34172	AB22852	0.79869	2.8879E-06
AB34172	AB29320	0.80741	1.8539E-06
AB34172	AB3071	-0.66057	0.00044255
AB34172	AB38635	0.67669	0.00028235
AB34172	AB5129	-0.69978	0.00014115
AB34172	AB6558	-0.74292	0.000032058
AB34451	AB2409	-0.63826	0.00079062
AB34896	AB1253	-0.90522	1.2261E-09
AB34896	AB14160	0.73391	0.0000447
AB34896	AB17447	0.81478	1.2519E-06
AB34896	AB19841	0.67739	0.00027671
AB34896	AB22204	-0.73739	0.000039377
AB34896	AB22730	-0.69391	0.00016935
AB34896	AB22852	-0.78174	6.4545E-06
AB34896	AB2417	-0.74522	0.00002939
AB34896	AB25200	0.74696	0.000027502
AB34896	AB28115	-0.75565	0.000019573
AB34896	AB29320	-0.82522	6.9669E-07
AB34896	AB29749	-0.7	0.00014019
AB34896	AB3071	0.67304	0.0003133
AB34896	AB34172	-0.79303	0.000003809
AB34896	AB35757	-0.74696	0.000027502
AB34896	AB35792	-0.76957	0.000011025
AB34896	AB36266	-0.77913	7.2594E-06
AB34896	AB38105	-0.69304	0.00017392
AB34896	AB38635	-0.68	0.00025659
AB34896	AB4160	0.68	0.00025659
AB34896	AB5129	0.75043	0.000024043
AB34896	AB5879	0.76	0.000016426
AB34896	AB6398	0.67304	0.0003133
AB34896	AB8595	0.74174	0.000033513
AB34910	AB17305	0.73652	0.000040653
AB34910	AB20919	-0.75304	0.000021707
AB34910	AB4160	-0.69217	0.0001786
AB35151	AB18149	0.84609	1.9066E-07
AB35151	AB30657	-0.65304	0.00054102
AB35151	AB37776	-0.85826	8.1747E-08
AB35155	AB12147	-0.66957	0.00034552
AB35155	AB16076	-0.74	0.000035759
AB35155	AB20940	0.67217	0.0003211
AB35155	AB27167	0.66	0.0004494
AB35155	AB31424	-0.72783	0.000055551
AB35208	AB1949	-0.65652	0.00049337
AB35208	AB2409	0.63043	0.00095901
AB35208	AB30418	-0.65217	0.00055353
AB35570	AB10273	-0.68261	0.00023776
AB35570	AB11013	-0.64174	0.00072438
AB35570	AB11344	-0.75826	0.000017626
AB35570	AB1253	0.67739	0.00027671
AB35570	AB12784	0.82174	8.5053E-07
AB35570	AB14160	-0.73739	0.000039377
AB35570	AB14616	-0.7113	0.000097481
AB35570	AB14788	-0.6513	0.00056629
AB35570	AB14975	-0.78609	5.2877E-06
AB35570	AB16227	-0.69826	0.00014804
AB35570	AB16898	-0.76079	0.000015907
AB35570	AB17447	-0.70522	0.00011879
AB35570	AB19439	-0.83739	3.3438E-07
AB35570	AB21646	-0.68972	0.00019242
AB35570	AB21658	0.64522	0.00066298
AB35570	AB22204	0.81826	0.000001034
AB35570	AB22852	0.67652	0.00028371
AB35570	AB23287	0.72948	0.000052386
AB35570	AB2417	0.66348	0.00040887
AB35570	AB24263	0.8087	1.7333E-06
AB35570	AB2526	-0.78	6.9817E-06
AB35570	AB26668	0.73391	0.0000447
AB35570	AB28385	-0.65565	0.00050493
AB35570	AB28610	0.77652	8.1519E-06
AB35570	AB29320	0.85217	1.2602E-07
AB35570	AB3071	-0.66174	0.00042872
AB35570	AB33467	0.81223	1.4366E-06
AB35570	AB33890	-0.6513	0.00056629
AB35570	AB34172	0.69325	0.00017284
AB35570	AB34896	-0.68783	0.00020367
AB35570	AB35757	0.66348	0.00040887
AB35570	AB35792	0.78783	4.8761E-06
AB35570	AB36266	0.66783	0.00036268
AB35570	AB37554	0.6313	0.00093889
AB35570	AB38105	0.68609	0.00021453
AB35570	AB38191	-0.71913	0.000075047
AB35570	AB38635	0.6687	0.00035401
AB35570	AB38882	0.74435	0.000030377
AB35570	AB501	0.68348	0.00023175
AB35570	AB5129	-0.66087	0.00043896
AB35570	AB643	-0.62957	0.00097949
AB35570	AB8595	-0.66348	0.00040887
AB35570	AB9015	-0.64957	0.00059259
AB35570	AB9593	0.70522	0.00011879
AB35757	AB10273	-0.77565	8.4703E-06
AB35757	AB11344	-0.71478	0.000086874
AB35757	AB1253	0.76348	0.000014239
AB35757	AB14283	-0.66609	0.00038058
AB35757	AB14788	-0.67565	0.00029087
AB35757	AB14901	0.71217	0.000094728
AB35757	AB19841	-0.87304	2.6152E-08
AB35757	AB21377	-0.8687	3.7086E-08
AB35757	AB21536	-0.86783	3.9711E-08
AB35757	AB22668	-0.63913	0.00077359
AB35757	AB22730	0.70522	0.00011879
AB35757	AB22852	0.62957	0.00097949
AB35757	AB25200	-0.75913	0.000017017
AB35757	AB29320	0.74609	0.000028432
AB35757	AB34172	0.84096	2.6659E-07
AB35757	AB35792	0.65304	0.00054102
AB35757	AB38635	0.77304	9.4926E-06
AB35757	AB5129	-0.72957	0.000052237
AB35757	AB6558	-0.78	6.9817E-06
AB35792	AB10273	-0.64348	0.00069309
AB35792	AB11344	-0.72261	0.000066627
AB35792	AB12147	0.7913	4.1373E-06
AB35792	AB14788	-0.72696	0.000057276
AB35792	AB1696	-0.80435	2.1717E-06
AB35792	AB22204	0.7913	4.1373E-06
AB35792	AB29320	0.80609	1.9857E-06
AB35792	AB3071	-0.75913	0.000017017
AB35792	AB31424	0.64609	0.00064835
AB35792	AB31803	-0.72609	0.000059048
AB35792	AB34172	0.64401	0.00068376
AB35792	AB36667	0.76522	0.000013246
AB35792	AB38635	0.68	0.00025659
AB35792	AB5129	-0.75652	0.000018904
AB35792	AB8930	0.73652	0.000040653
AB35801	AB27167	-0.64348	0.00069309
AB35801	AB33847	-0.86348	5.5507E-08
AB35801	AB34386	-0.6313	0.00093889
AB35827	AB13425	-0.80783	1.8141E-06
AB35827	AB24131	0.79478	3.4995E-06
AB35827	AB26225	0.72348	0.000064657
AB35827	AB28961	-0.71478	0.000086874
AB35827	AB3253	0.75217	0.000022462
AB35827	AB4600	-0.65826	0.00047094
AB36000	AB2409	0.74957	0.00002487
AB36000	AB6558	0.88087	1.3489E-08
AB36266	AB1253	0.75478	0.000020263
AB36266	AB13425	0.65826	0.00047094
AB36266	AB19841	-0.84	2.835E-07
AB36266	AB21377	-0.66348	0.00040887
AB36266	AB21536	-0.71826	0.000077292
AB36266	AB22204	0.72087	0.000070727
AB36266	AB22852	0.90783	9.1332E-10
AB36266	AB25200	-0.73739	0.000039377
AB36266	AB29320	0.78957	4.4932E-06
AB36266	AB34172	0.84314	2.3152E-07
AB36266	AB35757	0.77652	8.1519E-06
AB36266	AB38635	0.64348	0.00069309
AB36266	AB5129	-0.65565	0.00050493
AB36266	AB6558	-0.82696	6.2951E-07
AB36980	AB14901	0.69652	0.00015626
AB36980	AB15257	0.6887	0.00019842
AB36980	AB16076	0.68174	0.0002439
AB36980	AB20940	-0.79565	3.3545E-06
AB36980	AB21377	-0.71391	0.000089426
AB36980	AB22668	-0.72522	0.000060868
AB36980	AB3282	-0.84609	1.9066E-07
AB36980	AB34451	0.68348	0.00023175
AB36980	AB35151	-0.63304	0.00089975
AB36980	AB37331	0.63739	0.00080797
AB36980	AB4262	0.66376	0.00040571
AB37331	AB3253	0.6687	0.00035401
AB37331	AB4035	0.65913	0.00046006
AB37554	AB10273	-0.66087	0.00043896
AB37554	AB11013	-0.71739	0.000079596
AB37554	AB11547	0.72609	0.000059048
AB37554	AB1253	0.6687	0.00035401
AB37554	AB12784	0.63826	0.00079062
AB37554	AB13162	-0.66	0.0004494
AB37554	AB14084	0.64261	0.00070859
AB37554	AB14283	-0.74	0.000035759
AB37554	AB14616	-0.82348	7.7019E-07
AB37554	AB16898	-0.81046	1.5792E-06
AB37554	AB17370	-0.90783	9.1332E-10
AB37554	AB17447	-0.69826	0.00014804
AB37554	AB19439	-0.72783	0.000055551
AB37554	AB19841	-0.89217	4.7572E-09
AB37554	AB21377	-0.73913	0.000036931
AB37554	AB21536	-0.85478	1.0493E-07
AB37554	AB21646	-0.81279	1.3947E-06
AB37554	AB22218	0.69739	0.0001521
AB37554	AB22647	0.74522	0.00002939
AB37554	AB22852	0.77739	7.8442E-06
AB37554	AB23287	0.79827	2.9492E-06
AB37554	AB2409	-0.6313	0.00093889
AB37554	AB2417	0.7313	0.000049098
AB37554	AB25200	-0.83913	2.9964E-07
AB37554	AB2526	-0.75304	0.000021707
AB37554	AB26668	0.8487	1.6001E-07
AB37554	AB28110	-0.89043	5.6254E-09
AB37554	AB28115	0.77913	7.2594E-06
AB37554	AB28385	-0.81304	1.3755E-06
AB37554	AB28610	0.7687	0.000011441
AB37554	AB29320	0.77652	8.1519E-06
AB37554	AB33467	0.81398	1.3075E-06
AB37554	AB34172	0.78824	4.7831E-06
AB37554	AB34896	-0.68435	0.00022588
AB37554	AB35757	0.8487	1.6001E-07
AB37554	AB36000	-0.76783	0.00001187
AB37554	AB36266	0.86	7.1974E-08
AB37554	AB38105	0.83478	3.9327E-07
AB37554	AB38191	-0.77304	9.4926E-06
AB37554	AB38635	0.72348	0.000064657
AB37554	AB5129	-0.67043	0.00033721
AB37554	AB5879	-0.73652	0.000040653
AB37554	AB6398	-0.66696	0.00037154
AB37554	AB643	-0.73217	0.000047592
AB37554	AB6558	-0.88783	7.1964E-09
AB37554	AB8595	-0.6513	0.00056629
AB37776	AB38320	0.74957	0.00002487
AB38105	AB10273	-0.76087	0.000015853
AB38105	AB11344	-0.6887	0.00019842
AB38105	AB1253	0.70957	0.0001032
AB38105	AB14283	-0.70261	0.0001291
AB38105	AB14901	0.74174	0.000033513
AB38105	AB17447	-0.81565	1.1939E-06
AB38105	AB19841	-0.88	1.4552E-08
AB38105	AB21377	-0.88609	8.4523E-09
AB38105	AB21536	-0.87304	2.6152E-08
AB38105	AB22647	0.78522	5.5048E-06

AB38105	AB22668	-0.63913	0.00077359
AB38105	AB22730	0.72609	0.000059048
AB38105	AB23201	-0.70087	0.00013641
AB38105	AB2417	0.71304	0.000092043
AB38105	AB25200	-0.7313	0.000049098
AB38105	AB28115	0.84522	2.0198E-07
AB38105	AB29320	0.78261	6.2044E-06
AB38105	AB34172	0.84314	2.3152E-07
AB38105	AB35757	0.95826	1.9022E-13
AB38105	AB35792	0.66174	0.00042872
AB38105	AB36266	0.73565	0.000041965
AB38105	AB38635	0.76174	0.000015298
AB38105	AB5129	-0.71826	0.000077292
AB38105	AB5879	-0.70522	0.00011879
AB38105	AB6558	-0.75826	0.000017626
AB38105	AB8595	-0.72	0.000072859
AB38191	AB11702	-0.71217	0.000094728
AB38191	AB1253	-0.75217	0.000022462
AB38191	AB14616	0.77913	7.2594E-06
AB38191	AB17447	0.84783	1.697E-07
AB38191	AB19841	0.67391	0.00030566
AB38191	AB22204	-0.69478	0.00016489
AB38191	AB22647	-0.62957	0.00097949
AB38191	AB22730	-0.76522	0.000013246
AB38191	AB22852	-0.72261	0.000066627
AB38191	AB25200	0.7887	4.6812E-06
AB38191	AB28115	-0.7687	0.000011441
AB38191	AB29320	-0.83043	5.1223E-07
AB38191	AB29888	-0.66609	0.00038058
AB38191	AB34172	-0.71242	0.000093961
AB38191	AB34896	0.82957	5.3956E-07
AB38191	AB35757	-0.72696	0.000057276
AB38191	AB35792	-0.71217	0.000094728
AB38191	AB36266	-0.6887	0.00019842
AB38191	AB38105	-0.7087	0.00010616
AB38191	AB38635	-0.69739	0.0001521
AB38191	AB4160	0.72	0.000072859
AB38191	AB5129	0.8287	5.6818E-07
AB38191	AB5879	0.81391	1.3124E-06
AB38191	AB643	0.70261	0.0001291
AB38191	AB6558	0.63304	0.00089975
AB38191	AB8595	0.73391	0.0000447
AB38249	AB10086	0.68261	0.00023776
AB38249	AB1126	0.72261	0.000066627
AB38249	AB14084	-0.69043	0.00018828
AB38249	AB175	0.7287	0.000053871
AB38249	AB18149	0.75043	0.000024043
AB38249	AB20981	-0.69913	0.00014407
AB38249	AB21204	0.8487	1.6001E-07
AB38249	AB30657	-0.73913	0.000036931
AB38249	AB30755	-0.71565	0.000084386
AB38249	AB37776	-0.69826	0.00014804
AB38249	AB38320	-0.76174	0.000015298
AB38249	AB7155	-0.78348	5.9629E-06
AB38249	AB8505	-0.82609	6.6234E-07
AB38613	AB15257	0.6713	0.00032907
AB38613	AB17305	0.75304	0.000021707
AB38613	AB2409	-0.71391	0.000089426
AB38613	AB34451	0.80261	0.000002373
AB38613	AB4035	0.63826	0.00079062
AB38613	AB6490	0.69217	0.0001786
AB38635	AB22204	0.73739	0.000039377
AB38635	AB29888	0.8113	1.5098E-06
AB38635	AB30418	0.77043	0.000010623
AB38635	AB6558	-0.68087	0.00025017
AB38744	AB10398	0.73826	0.000038137
AB38744	AB1126	0.85478	1.0493E-07
AB38744	AB14084	-0.77043	0.000010623
AB38744	AB15695	-0.86348	5.5507E-08
AB38744	AB18149	0.74783	0.000026598
AB38744	AB29432	-0.75391	0.000020974
AB38744	AB30657	-0.90261	1.6324E-09
AB38744	AB30755	-0.84696	1.799E-07
AB38744	AB31424	0.65217	0.00055353
AB38744	AB35151	0.65304	0.00054102
AB38744	AB37776	-0.72261	0.000066627
AB38744	AB38320	-0.65304	0.00054102
AB38744	AB6494	0.82435	7.3262E-07
AB38744	AB7155	-0.83478	3.9327E-07
AB38838	AB10086	0.69826	0.00014804
AB38838	AB1126	0.68957	0.0001933
AB38838	AB11547	-0.62957	0.00097949
AB38838	AB14084	-0.67652	0.00028371
AB38838	AB20981	-0.65304	0.00054102
AB38838	AB21204	0.74522	0.00002939
AB38838	AB28110	0.65304	0.00054102
AB38838	AB29432	-0.7	0.00014019
AB38838	AB37776	-0.65304	0.00054102
AB38882	AB10273	-0.80435	2.1717E-06
AB38882	AB11344	-0.78174	6.4545E-06
AB38882	AB1253	0.71913	0.000075047
AB38882	AB12784	0.81304	1.3755E-06
AB38882	AB12943	-0.66174	0.00042872
AB38882	AB13162	-0.67565	0.00029087
AB38882	AB14616	-0.87478	2.266E-08
AB38882	AB14975	-0.76	0.000016426
AB38882	AB16898	-0.81002	1.6161E-06
AB38882	AB17370	-0.80348	2.2704E-06
AB38882	AB17447	-0.71043	0.0001003
AB38882	AB18208	0.65478	0.00051672
AB38882	AB19439	-0.85043	1.4211E-07
AB38882	AB19841	-0.77739	7.8442E-06
AB38882	AB21377	-0.72609	0.000059048
AB38882	AB21536	-0.74261	0.000032437
AB38882	AB21646	-0.88802	7.0679E-09
AB38882	AB21903	-0.78261	6.2044E-06
AB38882	AB22204	0.70957	0.0001032
AB38882	AB22647	0.69478	0.00016489
AB38882	AB22852	0.72261	0.000066627
AB38882	AB23287	0.90386	1.4245E-09
AB38882	AB2417	0.71826	0.000077292
AB38882	AB24263	0.66	0.0004494
AB38882	AB25200	-0.81739	0.000001085
AB38882	AB2526	-0.74783	0.000026598
AB38882	AB26668	0.84609	1.9066E-07
AB38882	AB28110	-0.72609	0.000059048
AB38882	AB28115	0.80696	1.8982E-06
AB38882	AB28385	-0.86522	4.8615E-08
AB38882	AB28610	0.82087	8.9343E-07
AB38882	AB29320	0.8687	3.7086E-08
AB38882	AB29888	0.65565	0.00050493
AB38882	AB33467	0.88385	1.0355E-08
AB38882	AB33890	-0.6713	0.00032907
AB38882	AB34172	0.74771	0.000026713
AB38882	AB34896	-0.74261	0.000032437
AB38882	AB35757	0.82174	8.5053E-07
AB38882	AB35792	0.7313	0.000049098
AB38882	AB36000	-0.64348	0.00069309
AB38882	AB36266	0.79739	3.0802E-06
AB38882	AB37554	0.85652	9.2693E-08
AB38882	AB38105	0.80435	2.1717E-06
AB38882	AB38191	-0.73826	0.000038137
AB38882	AB38635	0.88348	1.0707E-08
AB38882	AB501	0.68783	0.00020367
AB38882	AB5129	-0.69739	0.0001521
AB38882	AB6398	-0.67565	0.00029087
AB38882	AB643	-0.68522	0.00022014
AB38882	AB6558	-0.77043	0.000010623
AB38882	AB9593	0.68348	0.00023175
AB4035	AB19623	0.66957	0.00034552
AB4035	AB34451	0.74174	0.000033513
AB4035	AB35801	0.66696	0.00037154
AB4160	AB20919	0.69043	0.00018828
AB4160	AB29888	-0.64609	0.00064835
AB4160	AB35208	0.67565	0.00029087
AB4262	AB10273	-0.76769	0.000011937
AB4262	AB11013	-0.75896	0.000017135
AB4262	AB12784	0.67861	0.00026715
AB4262	AB14084	0.64367	0.00068964
AB4262	AB14283	-0.6559	0.00050159
AB4262	AB14616	-0.81485	0.000001247
AB4262	AB14788	-0.64804	0.00061649
AB4262	AB14901	0.83669	3.494E-07
AB4262	AB16898	-0.8	2.7059E-06
AB4262	AB17370	-0.8166	1.1333E-06
AB4262	AB17447	-0.7083	0.00010752
AB4262	AB18149	-0.68123	0.00024756
AB4262	AB19439	-0.67075	0.00033425
AB4262	AB19740	-0.6297	0.00097628
AB4262	AB19841	-0.8166	1.1333E-06
AB4262	AB2057	0.66608	0.00038061
AB4262	AB21377	-0.83494	3.8942E-07
AB4262	AB21536	-0.88036	1.4106E-08
AB4262	AB21903	-0.63057	0.00095577
AB4262	AB22647	0.66551	0.0003867
AB4262	AB22668	-0.76595	0.000012848
AB4262	AB23201	-0.81049	1.5768E-06
AB4262	AB2526	-0.76507	0.000013326
AB4262	AB25552	-0.64193	0.00072093
AB4262	AB26668	0.88472	9.5702E-09
AB4262	AB27281	-0.67948	0.00026048
AB4262	AB28110	-0.86464	5.0824E-08
AB4262	AB28610	0.64979	0.00058916
AB4262	AB3282	-0.6952	0.00016276
AB4262	AB33467	0.65702	0.00048689
AB4262	AB34172	0.73129	0.000049118
AB4262	AB35151	-0.66726	0.00036848
AB4262	AB35757	0.87774	1.7676E-08
AB4262	AB36266	0.68996	0.00019099
AB4262	AB37554	0.74761	0.000026825
AB4262	AB37776	0.71267	0.000093186
AB4262	AB38105	0.8821	1.21E-08
AB4262	AB38882	0.65328	0.00053764
AB4262	AB8595	-0.73014	0.000051184
AB4600	AB26225	-0.64087	0.00074047
AB501	AB11277	0.7513	0.000023241
AB501	AB11344	-0.71652	0.00008196
AB501	AB1253	0.64174	0.00072438
AB501	AB12943	-0.66957	0.00034552
AB501	AB14616	-0.73304	0.000046126
AB501	AB16898	-0.69978	0.00014115
AB501	AB17447	-0.70783	0.0001092
AB501	AB20919	-0.72174	0.00006865
AB501	AB21377	-0.66522	0.00038982
AB501	AB22730	0.68609	0.00021453
AB501	AB26668	0.65043	0.00057931
AB501	AB28115	0.7113	0.000097481
AB501	AB28385	-0.68174	0.0002439
AB501	AB29320	0.79217	3.9689E-06
AB501	AB29888	0.70957	0.0001032
AB501	AB30418	0.66522	0.00038982
AB501	AB33467	0.84106	2.6496E-07
AB501	AB34172	0.62876	0.0009988
AB501	AB34896	-0.69826	0.00014804
AB501	AB35792	0.66957	0.00034552
AB501	AB38105	0.63826	0.00079062
AB501	AB38191	-0.64	0.00075688
AB501	AB38635	0.64609	0.00064835
AB501	AB4160	-0.71565	0.000084386
AB501	AB5129	-0.64261	0.00070859
AB501	AB6541	-0.74783	0.000026598
AB501	AB9593	0.8487	1.6001E-07
AB5054	AB12147	-0.6887	0.00019842
AB5054	AB20919	0.76609	0.000012772
AB5054	AB27392	0.64435	0.00067789
AB5054	AB31424	-0.71826	0.000077292
AB5129	AB11344	0.67217	0.0003211
AB5129	AB22204	-0.72348	0.000064657
AB5129	AB29888	-0.71478	0.000086874
AB5129	AB30418	-0.65217	0.00055353
AB5129	AB3071	0.70783	0.0001092
AB5129	AB31803	0.85043	1.4211E-07
AB5129	AB38635	-0.70522	0.00011879
AB5173	AB31928	-0.73391	0.0000447
AB5483	AB13425	-0.68174	0.0002439
AB5483	AB13660	-0.65043	0.00057931
AB5483	AB17145	0.74696	0.000027502
AB5483	AB1949	0.72348	0.000064657
AB5483	AB22612	-0.63043	0.00095901
AB5483	AB33847	-0.64087	0.00074047
AB5483	AB6895	0.73043	0.000050646
AB5483	AB8577	0.70435	0.00012214
AB5879	AB1253	-0.70348	0.00012558
AB5879	AB17447	0.79913	2.8261E-06
AB5879	AB19841	0.79391	3.6502E-06
AB5879	AB20008	0.73826	0.000038137
AB5879	AB21377	0.72087	0.000070727
AB5879	AB22647	-0.75304	0.000021707
AB5879	AB22730	-0.6887	0.00019842
AB5879	AB25200	0.74957	0.00002487
AB5879	AB28115	-0.74348	0.000031392
AB5879	AB34172	-0.65839	0.00046932
AB5879	AB35757	-0.74435	0.000030377
AB5879	AB36266	-0.68783	0.00020367
AB5879	AB4160	0.64435	0.00067789
AB5879	AB5129	0.65739	0.00048204
AB5879	AB6558	0.65217	0.00055353
AB5879	AB8595	0.75652	0.000018904
AB6398	AB1253	-0.72	0.000072859
AB6398	AB13425	-0.87652	1.9593E-08
AB6398	AB22852	-0.73913	0.000036931
AB6398	AB23797	0.86609	4.5466E-08
AB6398	AB2417	-0.88435	9.9019E-09
AB6398	AB24262	-0.67913	0.00026315

AB6398	AB25200	0.76957	0.000011025
AB6398	AB3253	0.69304	0.00017392
AB6398	AB34172	-0.72244	0.000067011
AB6398	AB35757	-0.64522	0.00066298
AB6398	AB35827	0.83478	3.9327E-07
AB6398	AB36266	-0.69826	0.00014804
AB6398	AB6558	0.72696	0.000057276
AB643	AB1253	-0.82174	8.5053E-07
AB643	AB13425	-0.64783	0.00061993
AB643	AB14160	0.7687	0.000011441
AB643	AB14283	0.69304	0.00017392
AB643	AB17447	0.7113	0.000097481
AB643	AB19841	0.74696	0.000027502
AB643	AB22204	-0.69478	0.00016489
AB643	AB22852	-0.83826	3.1658E-07
AB643	AB23797	0.64522	0.00066298
AB643	AB2417	-0.84174	2.5355E-07
AB643	AB24262	-0.63826	0.00079062
AB643	AB25200	0.77739	7.8442E-06
AB643	AB27638	0.65652	0.00049337
AB643	AB28110	0.68957	0.0001933
AB643	AB28115	-0.82087	8.9343E-07
AB643	AB29320	-0.81391	1.3124E-06
AB643	AB29888	-0.65652	0.00049337
AB643	AB34172	-0.85752	8.627E-08
AB643	AB34896	0.77391	9.1403E-06
AB643	AB35757	-0.72	0.000072859
AB643	AB36266	-0.75565	0.000019573
AB643	AB38105	-0.75739	0.000018255
AB643	AB38635	-0.71739	0.000079596
AB643	AB5129	0.69739	0.0001521
AB643	AB6398	0.70522	0.00011879
AB643	AB6558	0.75652	0.000018904
AB6490	AB15257	0.77304	9.4926E-06
AB6490	AB18208	0.74	0.000035759
AB6490	AB2409	-0.7313	0.000049098
AB6490	AB29888	0.65217	0.00055353
AB6490	AB30418	0.64783	0.00061993
AB6494	AB1126	0.76957	0.000011025
AB6494	AB14084	-0.62957	0.00097949
AB6494	AB18149	0.66522	0.00038982
AB6494	AB24131	0.6687	0.00035401
AB6494	AB30657	-0.8687	3.7086E-08
AB6494	AB31424	0.65391	0.00052875
AB6494	AB38320	-0.73217	0.000047592
AB6494	AB8577	0.72522	0.000060868
AB6541	AB20919	0.79304	3.8066E-06
AB6558	AB2409	0.67391	0.00030566
AB6639	AB14788	-0.67826	0.00026986
AB6639	AB1491	-0.64	0.00075688
AB6639	AB1696	-0.86522	4.8615E-08
AB6639	AB18669	-0.81565	1.1939E-06
AB6639	AB26225	0.69565	0.00016052
AB6639	AB27392	-0.66261	0.00041869
AB6639	AB3071	-0.68957	0.0001933
AB6639	AB31424	0.63652	0.00082565
AB6639	AB31528	-0.64174	0.00072438
AB6639	AB31803	-0.75217	0.000022462
AB6639	AB36667	0.84261	2.3966E-07
AB6639	AB7164	-0.64	0.00075688
AB6639	AB8930	0.8487	1.6001E-07
AB6639	AB9798	-0.69217	0.0001786
AB6895	AB1949	0.7887	4.6812E-06
AB7155	AB10398	-0.81826	0.000001034
AB7155	AB1126	-0.78435	5.7298E-06
AB7155	AB14084	0.84348	2.2646E-07
AB7155	AB15695	0.84696	1.799E-07
AB7155	AB18149	-0.90783	9.1332E-10
AB7155	AB29432	0.80435	2.1717E-06
AB7155	AB30657	0.84522	2.0198E-07
AB7155	AB30755	0.89826	2.5849E-09
AB7155	AB35151	-0.83739	3.3438E-07
AB7155	AB37776	0.85391	1.1158E-07
AB7155	AB38320	0.66522	0.00038982
AB7155	AB8505	0.70696	0.00011232
AB7164	AB26225	-0.66522	0.00038982
AB7164	AB28961	0.6887	0.00019842
AB7164	AB31528	0.68261	0.00023776
AB7381	AB1253	-0.64522	0.00066298
AB7381	AB14160	0.68783	0.00020367
AB7381	AB34896	0.65217	0.00055353
AB836	AB16227	0.75391	0.000020974
AB836	AB1696	0.74957	0.00002487
AB836	AB17447	0.66696	0.00037154
AB836	AB22730	-0.6913	0.00018339
AB836	AB3071	0.73043	0.000050646
AB836	AB31803	0.87652	1.9593E-08
AB836	AB35792	-0.68348	0.00023175
AB836	AB36667	-0.69391	0.00016935
AB836	AB5129	0.73913	0.000036931
AB836	AB6639	-0.73391	0.0000447
AB836	AB8930	-0.77391	9.1403E-06
AB8505	AB1126	-0.76783	0.00001187
AB8505	AB11547	0.66348	0.00040887
AB8505	AB14084	0.70783	0.0001092
AB8505	AB18149	-0.72261	0.000066627
AB8505	AB21536	-0.66174	0.00042872
AB8505	AB24262	0.71478	0.000086874
AB8505	AB29432	0.63913	0.00077359
AB8505	AB30657	0.77391	9.1403E-06
AB8505	AB30755	0.73652	0.000040653
AB8505	AB37776	0.7113	0.000097481
AB8505	AB38320	0.70957	0.0001032
AB8577	AB1949	0.63913	0.00077359
AB8577	AB28961	-0.63478	0.00086202
AB8577	AB31528	-0.65739	0.00048204
AB8577	AB38320	-0.68696	0.00020904
AB8577	AB6895	0.71826	0.000077292
AB8595	AB10273	0.67391	0.00030566
AB8595	AB12147	-0.69217	0.0001786
AB8595	AB14901	-0.63478	0.00086202
AB8595	AB17447	0.80348	2.2704E-06
AB8595	AB19841	0.70348	0.00012558
AB8595	AB20940	0.81652	1.1383E-06
AB8595	AB21377	0.73217	0.000047592
AB8595	AB22204	-0.63043	0.00095901
AB8595	AB22668	0.76957	0.000011025
AB8595	AB22730	-0.75913	0.000017017
AB8595	AB29320	-0.69478	0.00016489
AB8595	AB3071	0.74	0.000035759
AB8595	AB31803	0.63826	0.00079062
AB8595	AB34172	-0.63486	0.00086037
AB8595	AB35757	-0.69565	0.00016052
AB8595	AB35792	-0.72522	0.000060868
AB8595	AB36266	-0.66087	0.00043896
AB8595	AB36667	-0.69217	0.0001786
AB8595	AB5129	0.70957	0.0001032
AB8595	AB8930	-0.6713	0.00032907
AB8930	AB12147	0.73043	0.000050646
AB8930	AB1696	-0.85043	1.4211E-07
AB8930	AB27392	-0.64174	0.00072438
AB8930	AB3071	-0.7687	0.000011441
AB8930	AB31424	0.76435	0.000013735
AB8930	AB31803	-0.71826	0.000077292
AB8930	AB36667	0.87739	1.8204E-08
AB8930	AB5129	-0.63652	0.00082565
AB9015	AB12784	-0.77826	7.5467E-06
AB9015	AB13425	-0.77043	0.000010623
AB9015	AB13660	-0.69565	0.00016052
AB9015	AB14788	0.63043	0.00095901
AB9015	AB17370	0.64348	0.00069309
AB9015	AB19439	0.74087	0.00003462
AB9015	AB22204	-0.64957	0.00059259
AB9015	AB2417	-0.77826	7.5467E-06
AB9015	AB24262	-0.63391	0.00088071
AB9015	AB24263	-0.7313	0.000049098
AB9015	AB26668	-0.64957	0.00059259
AB9015	AB28610	-0.68174	0.0002439
AB9015	AB33890	0.7	0.00014019
AB9219	AB15695	0.68	0.00025659
AB9219	AB16227	0.73913	0.000036931
AB9219	AB1696	0.77217	9.8569E-06
AB9219	AB31424	-0.62957	0.00097949
AB9219	AB36667	-0.70174	0.00013271
AB9219	AB6639	-0.63739	0.00080797
AB9219	AB8930	-0.65652	0.00049337
AB928	AB10458	-0.82174	8.5053E-07
AB928	AB11344	-0.64957	0.00059259
AB928	AB12147	0.65304	0.00054102
AB928	AB14788	-0.66435	0.00039924
AB928	AB14975	-0.66957	0.00034552
AB928	AB15099	-0.73478	0.000043313
AB928	AB15695	-0.76957	0.000011025
AB928	AB16227	-0.85391	1.1158E-07
AB928	AB16638	0.74625	0.000028257
AB928	AB1696	-0.8687	3.7086E-08
AB928	AB17891	-0.87043	3.2298E-08
AB928	AB21658	0.68696	0.00020904
AB928	AB23737	0.70348	0.00012558
AB928	AB24263	0.76348	0.000014239
AB928	AB24846	-0.69217	0.0001786
AB928	AB29350	0.8	2.7061E-06
AB928	AB3071	-0.65043	0.00057931
AB928	AB30977	-0.67565	0.00029087
AB928	AB31424	0.70348	0.00012558
AB928	AB31803	-0.72348	0.000064657
AB928	AB35792	0.73391	0.0000447
AB928	AB36667	0.73478	0.000043313
AB928	AB38744	0.7287	0.000053871
AB928	AB5129	-0.6687	0.00035401
AB928	AB6639	0.76	0.000016426
AB928	AB836	-0.73826	0.000038137
AB928	AB8930	0.74696	0.000027502
AB928	AB9219	-0.77739	7.8442E-06
AB928	AB9593	0.67739	0.00027671
AB9309	AB13425	0.78174	6.4545E-06
AB9309	AB13660	0.67913	0.00026315
AB9309	AB15257	-0.66	0.0004494
AB9309	AB16076	-0.64174	0.00072438
AB9309	AB1949	-0.85652	9.2693E-08
AB9309	AB23797	-0.68783	0.00020367
AB9309	AB24002	-0.79043	0.000004312
AB9309	AB24131	-0.83043	5.1223E-07
AB9309	AB28961	0.86087	6.7492E-08
AB9309	AB35827	-0.75217	0.000022462
AB9309	AB38320	0.73913	0.000036931
AB9309	AB5483	-0.65043	0.00057931
AB9309	AB6398	-0.69826	0.00014804
AB9309	AB6494	-0.70522	0.00011879
AB9309	AB6895	-0.72261	0.000066627
AB9309	AB8577	-0.6713	0.00032907
AB9593	AB11277	0.72261	0.000066627
AB9593	AB11344	-0.63652	0.00082565
AB9593	AB14160	-0.64609	0.00064835
AB9593	AB14616	-0.67217	0.0003211
AB9593	AB16227	-0.68174	0.0002439
AB9593	AB17447	-0.70087	0.00013641
AB9593	AB20919	-0.65217	0.00055353
AB9593	AB22730	0.67565	0.00029087
AB9593	AB28115	0.64609	0.00064835
AB9593	AB29320	0.77217	9.8569E-06
AB9593	AB31456	0.66174	0.00042872
AB9593	AB34896	-0.67565	0.00029087
AB9593	AB35792	0.74783	0.000026598
AB9593	AB4160	-0.67478	0.00029819
AB9593	AB5129	-0.71391	0.000089426
AB9593	AB6541	-0.66609	0.00038058
AB9798	AB10273	0.7313	0.000049098
AB9798	AB12147	-0.65565	0.00050493
AB9798	AB14788	0.69391	0.00016935
AB9798	AB14901	-0.69565	0.00016052
AB9798	AB1696	0.80957	1.6557E-06
AB9798	AB35792	-0.73739	0.000039377
AB9798	AB36667	-0.72261	0.000066627
AB9798	AB8930	-0.74174	0.000033513
